# *Pyropia yezoensis* genome reveals diverse mechanisms of carbon acquisition in the intertidal environment

**DOI:** 10.1038/s41467-020-17689-1

**Published:** 2020-08-12

**Authors:** Dongmei Wang, Xinzi Yu, Kuipeng Xu, Guiqi Bi, Min Cao, Ehud Zelzion, Chunxiang Fu, Peipei Sun, Yang Liu, Fanna Kong, Guoying Du, Xianghai Tang, Ruijuan Yang, Junhao Wang, Lei Tang, Lu Wang, Yingjun Zhao, Yuan Ge, Yunyun Zhuang, Zhaolan Mo, Yu Chen, Tian Gao, Xiaowei Guan, Rui Chen, Weihua Qu, Bin Sun, Debashish Bhattacharya, Yunxiang Mao

**Affiliations:** 1 Key Laboratory of Marine Genetics and Breeding (OUC), Ministry of Education, 266100 Qingdao, China; 2 College of Marine Life Sciences, Ocean University of China, 266100 Qingdao, China; 3 Department of Biochemistry and Microbiology, Rutgers University, New Brunswick, NJ 08901 USA; 4 Shandong Provincial Key Laboratory of Energy Genetics, Key Laboratory of Biofuels, Qingdao Institute of BioEnergy and Bioprocess Technology, Chinese Academy of Sciences, 266101 Qingdao, China; 5 Key Laboratory of Marine Environment and Ecology, Ministry of Education, Ocean University of China, 266100 Qingdao, China; 6 Key Laboratory of Maricultural Organism Disease Control, Ministry of Agriculture and Rural Affairs, Yellow Sea Fisheries Research Institute, Chinese Academy of Fishery Sciences, 266071 Qingdao, China; 7 Key Laboratory of Utilization and Conservation for Tropical Marine Bioresources (Hainan Tropical Ocean University), Ministry of Education, 572022 Sanya, China; 8 The Laboratory for Marine Biology and Biotechnology, Qingdao National Laboratory for Marine Science and Technology, 266237 Qingdao, China

**Keywords:** Genome evolution, Ecological genetics, Evolutionary genetics, Plant evolution

## Abstract

Changes in atmospheric CO_2_ concentration have played a central role in algal and plant adaptation and evolution. The commercially important red algal genus, *Pyropia* (Bangiales) appears to have responded to inorganic carbon (C_i_) availability by evolving alternating heteromorphic generations that occupy distinct habitats. The leafy gametophyte inhabits the intertidal zone that undergoes frequent emersion, whereas the sporophyte conchocelis bores into mollusk shells. Here, we analyze a high-quality genome assembly of *Pyropia yezoensis* to elucidate the interplay between C_i_ availability and life cycle evolution. We find horizontal gene transfers from bacteria and expansion of gene families (e.g. carbonic anhydrase, anti-oxidative related genes), many of which show gametophyte-specific expression or significant up-regulation in gametophyte in response to dehydration. In conchocelis, the release of HCO_3_^-^ from shell promoted by carbonic anhydrase provides a source of C_i_. This hypothesis is supported by the incorporation of ^13^C isotope by conchocelis when co-cultured with ^13^C-labeled CaCO_3_.

## Abstract (web summary)

The nori producing seaweed *Pyropia yezoensis* has heteromorphic generations that occupy distinct habitats. Here, via genome assembly, transcriptome analysis, and 13 C isotope labeling, the authors show the interplay between inorganic carbon availability and life cycle evolution in the intertidal environment.

## Introduction

**Fig. 1:**
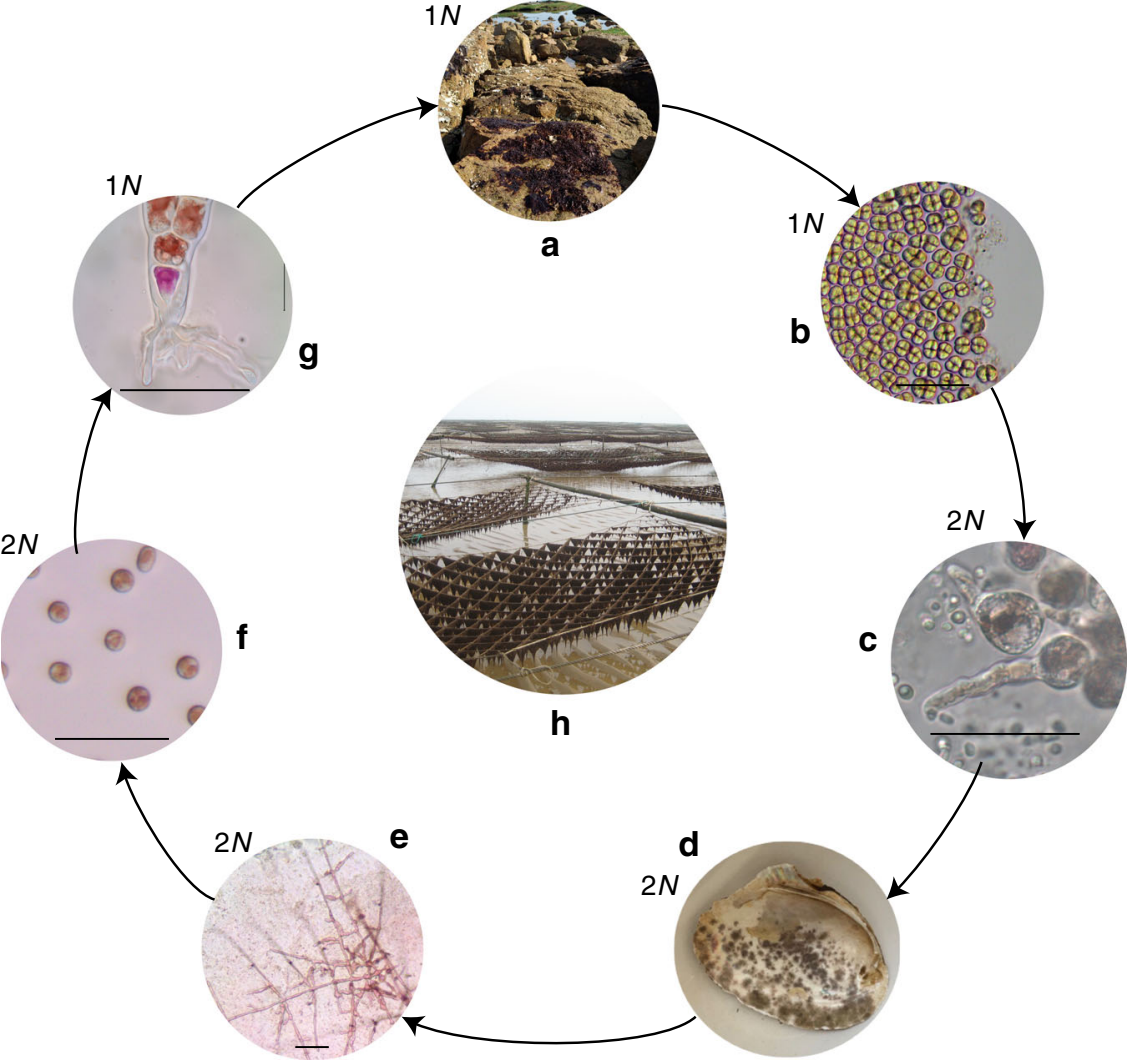
The life cycle of *P. yezoensis* showing the alternation of heteromorphic generations inhabiting distinctive niches. Ploidy was added as “1*N*” and “2*N*” in the upper left corner of each subgraph. **a** Gametophyte thalli adhered to rocks are exposed to air during low tide. **b** Carpospores and sperms form in mature thalli; **c** released zygotospores, showing one with a germ tube. **d** Purple patches on oyster shell are boring sporophyte conchocelis. **e** Swelling filamentous conchocelis that are going to produce conchosporangia. **f** Released conchospores. **g** Thallus seedling with a holdfast formed at the base. **h***P. yezoensis* nets in mariculture farms. Scale bars in **b**, **c**, and **e**–**g** 50 μm. Asexual reproduction is not shown here. Microscope observation in **b**, **c** and **e**, **g** were repeated independently on multiple *Pyropia* samples (*n* > 10) showing similar morphology.

The seaweed used most frequently to produce *nori* (sushi wrap), *Pyropia yezoensis*, has a high nutritional value, including up to 25–30% protein by dry weight, vitamins (in particular, B12), and oligosaccharides. The mariculture of *nori* in countries such as Korea, Japan, and China reaches an annual production of ca. 1.8 million tons^1^, making it, globally, one of the most economically important marine crops. *P. yezoensis* is a member of the order Bangiales (family Bangiophyceae) in the red algae (Rhodophyta). Molecular phylogenetic studies, which incorporate the fossil record, suggest that Bangiophyceae diverged from their sister group, the Florideophyceae, around 900 million years ago (Mya). More limited phylogenomic data indicate that the radiation of species within the Bangiophyceae occurred about ~250 Mya ago in the Permo-Carboniferous era, leading to formation of the order Bangiales^2,3^. Species in Bangiales, for instance, *Pyropia* spp. (e.g., *P. yezoensis*, *P. haitanensis*, *Porphyra umbilicalis*) and *Bangia* spp., typically have a life cycle characterized by the alternation of heteromorphic generations that live in distinctive habitats (Fig. 1). The edible, leafy thallus (gametophyte) inhabits the upper intertidal zone of exposed coasts. These seaweeds attach to rocky substrata using a rhizoidal holdfast (Fig. 1a, g) and are exposed to air during low tide. Many intertidal algae, including *P. yezoensis*, can utilize atmospheric CO_2_ as the carbon source for photosynthesis^4–6^. However, frequent emersion also leads to fluctuating environmental stresses, such as extreme drought, high ultraviolet (UV) irradiance, and high temperature. For this reason, the *P. yezoensis* thallus, which is capable of tolerating broad and extreme environmental stresses, is of interest as a model for studying molecular mechanisms of stress resistance. Photosynthesis in the thallus can recover rapidly from >80% water loss that results in the death of unwanted epiphytes^7^. This robustness facilitates commercial *nori* cultivation in the open sea.

In contrast to *P. haitanensis* (another edible *nori* species), *P. yezoensis* is monecious. Mature thalli release sperm to fertilize carpospores (Fig. 1b, c). The resulting diploid zygotospores germinate into the filamentous and branched conchocelis phase (the sporophyte phase). The conchocelis burrows into the inner layers of mollusk shells and lives within the calcareous matrix (Fig. 1d, e). The shell appears to provide protection from UV damage and wave exposure^8^. However, CO_2_ is scarce in the enclosed space that has reduced water exchange, which may limit growth of the conchocelis. Past work shows that increased atmospheric CO_2_ does not affect conchocelis photosynthetic performance, suggesting the existence of an alternative carbon source^6^. Conchosporangia develop in mature conchocelis and release conchospores that attach to rocky substrata or *P. yezoensis* nets in mariculture and develop into thalli, i.e., seedlings (Fig. 1f, g).

*Pyropia* species appear to have evolved two distinct strategies that are reflected in their life cycle: (1) the gametophyte thallus utilizes atmospheric CO_2_ while living in a harsh environment and (2) the sporophyte conchocelis is protected in the shell environment but faces C_i_ limitation. The genome-wide impact of this alternation of heteromorphic generations is poorly understood. The genomes of *P. umbilicalis* and *P. haitanensis* have recently been published^9,10^. Here we present the genome of *P. yezoensis* and analyze Bangiales genome and transcriptomic data to gain insights into the evolutionary mechanisms that allow these species to thrive in their stressful habitats vis-à-vis their dimorphic life cycle.

## Results

### Genome assembly

Assembly of the *P. yezoensis* genome using PacBio long-read sequencing data followed by removal of bacterial contaminant sequences resulted in 660 contigs with N50 = 340.8 kbp (Supplementary Tables 1 and 2; Supplementary Figs. 1 and 2). The genome size was estimated to be ca. 108 Mbp, which is bigger than *P. umbilicalis* albeit comparable to the florideophyte *Chondrus crispus*. Hi-C and BioNano data linked the contigs into three scaffolds, S1 = 29.4 Mbp, S2 = 34.3 Mbp, and S3 = 43.6 Mbp, equivalent to the three chromosomes observed in haploid *P. yezoensis*^11^ (Supplementary Tables 1–3, Supplementary Figs. 3 and 4). These scaffolds included 95% of the genome data with the remaining 524 contigs comprising 5.5 Mbp. Nearly one-half of the *P. yezoensis* genome (48.0%) is predicted to be repeat elements, of which long terminal repeat (LTR) retrotransposons are predominant (Supplementary Note 1, Supplementary Table 4). The numbers of intact LTR is positively correlated with genome size in red algae (*R*^2^ = 0.9437), explaining genome size growth in *Pyropia* (Supplementary Fig. 5) and *Gracilariopsis* genome^12^.

Combining ab initio prediction with PacBio transcriptome data, 12,855 protein-coding genes were identified in the *P. yezoensis* genome (Supplementary Table 3). These contain 84% of the core eukaryotic single-copy genes in the BUSCO database^13^, supporting the completeness of the assembly (Supplementary Fig. 6). To elucidate the evolution of genome structure in Bangiophyceae, we compared gene synteny in *P. yezoensis*, *P. haitanensis*, and *P. umbilicalis*. Long syntenic regions were identified in the two *Pyropia* genomes, whereas only a limited amount of synteny was found between *P. yezoensis* and *P. umbilicalis* (Supplementary Note 2, Supplementary Fig. 7). These results suggest that multiple genome rearrangements occurred after the divergence of these two genera.

### Expanded orthogroups (OGs) and their phase-specific transcription

**Fig. 2:**
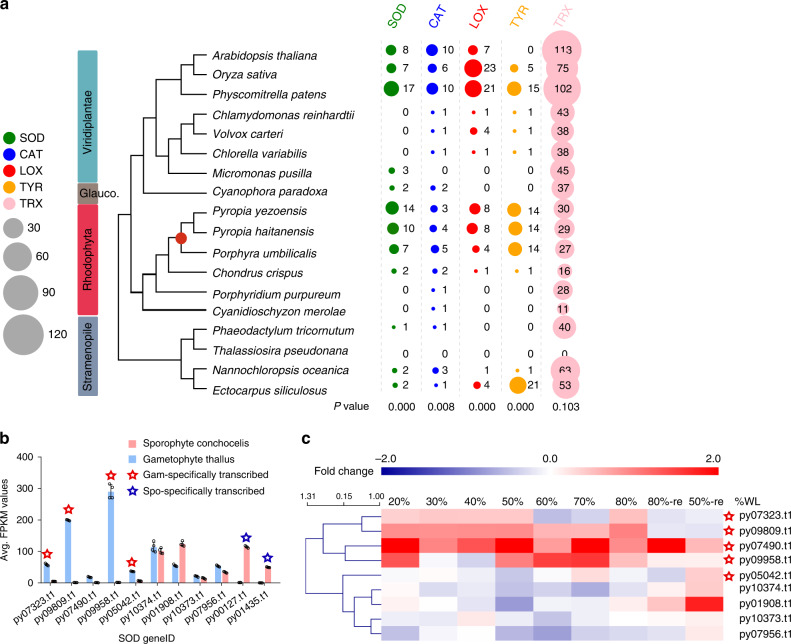
The copy numbers of genes related to antioxidative functions and the transcriptional pattern of SOD genes. **a** Copy numbers of SOD, CAT, LOX, TYR, and TRX genes in algal genomes. The dendrogram was generated based on the phylogenetic tree constructed based on the 424 single-copy orthologous gene sets identified from *P. yezoensis* and the other 17 published algal genomes. The expanded orthogroups related to antioxidative functions are shown in different colors. The size of the circle represents gene copy number. *P* values are listed at the bottom of each column. **b** Transcriptional level of SOD paralogs in the two life phases. Columns and vertical bars represent the average FPKM values of SOD genes in individual life phase and SD (blue: gametophyte; red: sporophyte; *n* = 4 biological independent samples), respectively. Red and blue stars indicate the gam-specifically transcribed and spo-specifically transcribed genes, respectively. **c** Heatmap showing the fold change of SOD paralog transcripts in response to osmotic stress. Fold change was calculated as log_2_(FPKM(*x*% of water loss or rehydration)/FPKM (ctrl)). Percentages above the heatmap denote the degree of water loss and 80%-re and 50%-re denote the rehydrated samples after 80 and 50% of water loss, respectively. WL water loss.

To gain insights into the origin of stress tolerance pathways in the *P. yezoensis* genome, we inferred orthologous relationships between the three available Bangiales genomes and 15 other genomes representing Florideophyceae, green algae, heterokonts, and plants. A total of 10,327 OGs were found, of which 424 single-copy OGs were used to build a concatenated gene phylogeny (Supplementary Fig. 8). These data were used to estimate the divergence time of Bangiales, which was found to be during the Permo-Carboniferous glaciation (300–225 Mya, Supplementary Note 3, Supplementary Fig. 8), consistent with previous studies^2,3^. A total of 578 OGs were significantly expanded in Bangiales when compared to Florideophyceae and include the following Pfam annotations: antioxidative systems (e.g., thioredoxin (TRX), catalase (CAT), Cu/Zn-superoxide dismutase (SOD), carboxymuconolactone decarboxylase, D-arabinono-1,4-lactone oxidase), stress resistance (e.g., lipoxygenase, HSP20, bacterial low temperature requirement A protein, subtilase, beta-lactamase), detoxification, carbohydrate metabolism (GH17, GH27, GH36, GH53, GH70, GH85, GH97, GT2, GT21, CMB, CMB53), transport, and tyrosine metabolism (Fig. 2a and Supplementary Fig. 9).

To determine whether a link exists between OG expansion and life history stage, we studied the differential expression of genes (DEGs) in *P. yezoensis* when comparing the gametophyte thallus (gam) and the sporophyte conchocelis (spo). Dually expressed genes with fragments per kilobase of transcript per million mapped reads (FPKM) <5 in gam and >25 in spo and unique genes with >10-fold FPKM in spo were defined as spo-specifically transcribed genes. Gam-specifically transcribed genes were defined in the same way in the opposite direction. Genes with FPKM > 5 in both generations and fold change <10 were considered to be constitutively expressed. Because desiccation is one of the most important environmental stresses faced by *P. yezoensis* thalli in the intertidal zone, we studied gene expression patterns during a time course of desiccation and rehydration. In this regard, SOD catalyzes the dismutation of two molecules of the superoxide radical into one molecule of oxygen and one molecule of hydrogen peroxide (H_2_O_2_), which can be degraded into water and oxygen in the presence of catalase. Thus the antioxidant activity of SODs is of particular importance to the amelioration of cell damage induced by reactive oxygen species (ROS) and maintenance of cell homeostasis. In plants, the SOD family consists of three classes of metalloenzymes, Fe-SOD, Mn-SOD, and Cu/Zn-SOD. In the *P. yezoensis* genome, we found 14 SOD genes, whereas this number is 7, 3, and 3 in *C. crispus*, *Porphyridium purpureum*, and *Cyanidioschyzon merolae*, respectively (Fig. 2a). DEG analysis showed that SODs in the conchocelis and thallus, as well as under dehydration-stressed conditions, can be categorized into three groups: (1) spo-specifically transcribed SODs that include two genes exhibiting high relative transcriptional levels in sporophyte (py00127.t1, py01435.t1); (2) five gam-specifically transcribed SOD genes; (3) the remaining four genes show constitutive expression in the two stages (Fig. 2b). Interestingly, 4/5 gam-specifically transcribed SOD genes exhibit significant upregulation under dehydration, whereas the other genes show minimal expression variation (Fig. 2c). The correlation between SOD gene expansion, specific expression in the gametophyte thallus, and burst of transcriptional activity after water loss suggest that SODs played a significant role in the origin and maintenance of the heteromorphic life cycle of *P. yezoensis* as a response to life in the intertidal zone. In addition to SOD, several other oxidative stress-related OGs, including lipoxygenase (LOX), catalase, and tyrosinase (TYR) have expanded gene numbers in *Pyropia* species (Fig. 2a). Members of these gene families exhibit similar transcriptional patterns to SODs, with upregulation in a specific life history stage and high expression of gam-specifically transcribed genes under osmotic stress (Supplementary Note 4, Supplementary Figs. 10 and 11). The conserved response across these different gene families hints at the evolution of a regulatory mechanism in Bangiales for life in the intertidal zone.

### C_i_ assimilation

**Fig. 3:**
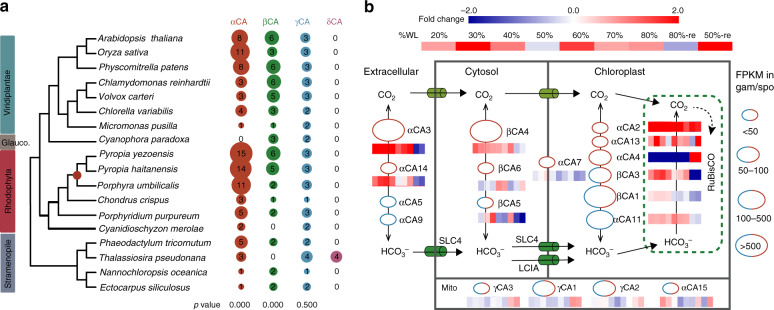
Analysis of carbonic anhydrase analysis. **a** Carbonic anhydrase genes in model photosynthetic organisms. The dendrogram was constructed as described in Fig. 2. The copy number of genes for each CA subfamily is represented by the size of each circle. The red circle in the phylogeny indicates the divergence point of Bangiales. **b** The proposed schematic diagram showing the possible organization of CA isoforms in the CCM of *Pyropia*. CA isoforms are represented by ellipses with red and blue colors standing for their transcription preference in gametophyte and sporophyte, respectively. Ellipses with both colors are CAs that are transcribed in both stages. The size of each ellipse is determined by its transcriptional level (the region of avg. FPKM value) in corresponding stage. The subcellular localization of CA isoforms and HCO_3_^−^ transporters are based on PredAlgo prediction and transient expression of GFP fusion proteins in tobacco. α-CA7 that are predicted to harbor transmembrane helixes by TMHMM are placed across the chloroplast membrane. Plastid-targeting CAs might function in either chloroplast stroma or thylakoid (green dashed square) where RuBisCO is localized. Heatmap beside or under each CA isoform shows the transcriptional variation in response to osmotic stress. Fold changes were calculated as in Fig. 2. No heatmaps are shown for α-CA5 and α-CA9 because they are exclusively transcribed in sporophyte (blue ellipses), except for α-CA11 which is transcribed in gametophytes at a much lower level than in the sporophyte.

Beyond environmental stresses, pathways of C_i_ acquisition are common targets for selection in algae and plants. Therefore, it is significant that one of the most prominent cases of gene expansion in *P. yezoensis* is for carbonic anhydrase (CA) encoding sequences. As a key component of the carbon-concentrating mechanism (CCM) in photosynthetic organisms, intracellular CA catalyzes the reversible conversion of carbon dioxide to bicarbonate, preventing the escape of CO_2_ through cellular membranes. Bicarbonate is stored in the pyrenoid and subsequently converted to CO_2_ by CA near the active site of RuBisCO to allow efficient carbon fixation. CA genes are well characterized in *Chlamydomonas reinhardtii*, diatoms, and plants^14–18^. Both internal and external CA are required for the uptake of HCO_3_^−^ in *Porphyra*^19,20^. There are six CA subfamilies (α, β, γ, δ, ε, η) that exhibit high protein sequence divergence and different subcellular localizations. We found 24, 22, and 16 putative CA homologs in *P. yezoensis*, *P. haitanensis*, and *P. umbilicalis*, respectively, which greatly exceeds the number in Florideophyceae, green algae, and algae with secondary plastids, such as diatoms. CA genes are distributed across the three *P. yezoensis* chromosomes. A total of 16 out of the 24 CAs formed conserved orthologous groups with counterparts in *P. haitanensis* in collinear blocks that are shared by these two species (Supplementary Note 2, Supplementary Fig. 12). Gene expansion in *P. yezoensis* is primarily explained by αCAs (15 copies) and βCAs (6 copies). The number of γCAs did not show significant variation among photosynthetic organisms (Fig. 3a).

The αCAs in *P. yezoensis* show relatively low protein identity (pairwise values ranging from 37 to 87%) and are distributed in seven subgroups based on protein sequence similarity. Eleven of these *P. yezoensis* genes are grouped with the corresponding orthologs from *P. umbilicalis* (Supplementary Fig. 13). The lack of *P. umbilicalis* orthologs of the other four αCAs in *P. yezoensis* may be attributed to gene loss or missing data. Most αCAs harbor one typical CA domain, however, in the longest protein αCA15 (and Pu-αCA11) that is of length 1337 amino acids, four CA domains are tandemly repeated (Supplementary Fig. 14). This multi-domain CA was limited to *P. yezoensis*. Although most α-CAs are not considered to form multimers, several studies with fungi^21^, *C. reinhardtii*^22^, and animals^23^ report dimer organization of α-CA. Dimers, tetramers, and octamers are often found for βCAs that form multiple active sites^24^. Therefore, the four CA domains in αCA15 likely enhance enzyme stability and activity.

Based on signal peptide prediction using PredAlgo, nine, four, five, and six CA isoforms may be localized in plastid, mitochondria, extracellular matrix, and other regions in the cytoplasm, respectively (Fig. 3b, Supplementary Fig. 13). The extracellular localization of αCA5 and αCA9, as well as the cytoplasmic localization of βCA4, were confirmed using transient expression of green fluorescent protein (GFP) fusion proteins in tobacco (Supplementary Table 5, Supplementary Fig. 15). Although αCA14 was predicted to be a cytosolic proteins, it was clearly localized to the extracellular matrix in tobacco. The following CAs αCA2, αCA4, αCA7, αCA13, αCA11, βCA1 and βCA3 were plastid-targeted CAs, although their suborganellar localization were unclear. These proteins might be involved in the conversion of CO_2_ and HCO_3_^−^ in the plastid stroma or the thylakoid. The αCA7 harbors nine trans-membrane helices, suggesting that it might be associated with the plastid envelope (Fig. 3b). We determined whether these CA isoforms were transcriptionally active in the leafy thallus and conchocelis. Of these, αCA2, αCA3, αCA4, αCA7, αCA13, βCA4, and βCA6 were exclusively expressed in the thallus (gam-specifically transcribed CAs) and α-CA14 had significantly higher expression (>3-fold) in the thallus than in the conchocelis (Supplementary Fig. 16). We investigated their gene expression patterns during dehydration. Apart from the plastidal membrane-anchoring αCA7, all of the gam-specifically transcribed CAs exhibited elevated transcription in at least one stage of osmotic stress, yet the expression patterns of the individual CAs were markedly different. The extracellular αCA3, with a relatively high expression level, showed increased transcription during dehydration from 20 to 80% water loss, followed by a decrease when rehydrated. Similar transcriptional patterns were observed with the other extracellular αCA13, the cytoplasmic βCA4, as well as the plastidal αCA13 and βCA3. Plastidal αCA4 exhibited an opposite pattern, with low expression under dehydration and a 13-fold increase when immersed after 80% water loss. Transcription of αCA2, another gam-specifically transcribed isoform, was upregulated during both dehydration and rehydration, and βCA6 was upregulated during the later stages of desiccation (Fig. 3b). Overall, the enhanced expression of *P. yezoensis* CAs at different stages of osmotic stress, as well as their likely localization in diverse subcellular compartments, suggests an essential role in CO_2_ provision in the two distinct life history stages of this red seaweed. One and three genes encoding the putative bicarbonate transporter LCIA and SLC4, respectively, were also identified in the *P. yezoensis* genome and all of them were constitutively expressed in the two life stages (Supplementary Note 5, Supplementary Fig. 17), facilitating the import of HCO_3_^−^ in the CCM.

**Fig. 4:**
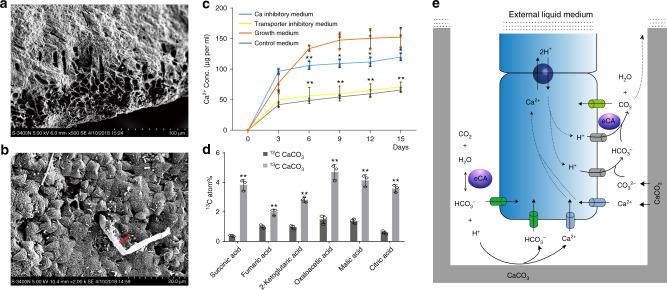
The digestion of shell CaCO_3_ by the conchocelis and the function of extracellular carbonic anhydrase. **a** Boreholes in the calcareous matrix of mollusk shells occupied by *Pyropia* conchocelis. **b** Microscopic image of conchocelis living in mollusk shells. The red arrow indicates the conchocelis in the borehole in shell calcareous matrix. Microscopic observation in **a** and **b** were repeated independently on multiple shell conchocelis (*n* > 10) showing similar structure. **c** The variation of Ca^2+^ release from conchocelis-dwelling shell w/o inhibitors of carbonic anhydrase or P-type transporter. Triplets were done for each condition. Ca^2+^ concentrations in control medium, growth medium, CA inhibitory medium, and transporter inhibitory medium were plotted in gray, red, blue, and yellow, respectively. Time refers to the duration (in days) since the addition of inhibitors. The following comparisons were done: growth vs control, growth vs CA inhibitory or P-type ATPase inhibitory medium. Vertical bars represent the SD (*n* = 3 biological independent samples). *P* value in each comparison was calculated by two-sided independent *t* test in SPSS. Variations with *P* < 0.05 were considered to be significant as indicated by asterisks. One asterisk: 0.01 < *P* < 0.05; two asterisks: *P* < 0.01. **d** The abundance of ^13^C isotope in organic acids of TCA cycle in conchocelis co-cultured with ^12^C-CaCO_3_ or ^13^C-CaCO_3_. Columns and vertical bars represent the mean and SD, respectively (*n* = 3 biological independent samples). Statistical analysis was done by two-sided independent *t* test in SPSS. Two asterisks indicated *P* < 0.01. **e** A working model for the dissolution of shell CaCO_3_ and utilization of generated carbon flux in conchocelis. Only the apical conchocelis cell and part of its adjacent cell are shown in this model. Extracellular CA was represented by purple ovens. Transporters or channels of bicarbonate, CO_2_, Ca^2+^, and protons are indicated by green, light green, blue, and gray cylinders, respectively, across the cell membrane. The P-type calcium transporter is indicated by the dark blue circle. Source data underlying Fig. 4c, d are provided as a Source data file.

Interestingly, we also found three spo-specifically transcribed CAs and another three that exhibit higher expression in the conchocelis than in the thallus (Supplementary Fig. 16). Two of them (αCA5 and αCA9) were experimentally validated to be secreted proteins (Supplementary Fig. 13, Supplementary Fig. 15). The external CA enzymatic activity in free conchocelis was 7.86 Units per g fresh weight, slightly higher than the value previously reported in the *Pyropia* thallus^25^. Extracellular CAs in microbes can accelerate calcium carbonate dissolution in the presence of H^+^ that is produced during the hydration of CO_2_^26,27^. Conchocelis live en masse in boreholes in the calcareous matrix of mollusk shells (Fig. 4a, b). The nature of evolutionary forces driving conchocelis burrowing into shell and how these cells acquire sufficient C_i_ to support photosynthetic growth are open questions. Given that shells are composed primarily of calcium carbonate, we tested whether the conchocelis utilizes this substrate as a carbon source with the aid of extracellular CAs. To do this, we determined the Ca^2+^ concentration and pH near the shell in control medium (alga-free shell), growth medium (conchocelis-dwelling shell), and CA inhibitory medium (conchocelis-dwelling shell with the CA inhibitor acetazolamide). The Ca^2+^ concentration in the growth medium increased rapidly and continuously, until reaching a plateau on the 15th day when the conchocelis covered all of the shell. These values in the control and inhibitory media showed mild increases in the initial 3 days and went up gradually at a similar rate, possibly due to the natural release of Ca^2+^ from the shell matrix (Fig. 4c). The increasing rates over the first 3 days in the inhibitory and control medium were slightly different, possibly attributed to the incomplete inhibition of CAs because it may have taken some time for the acetazolamide to enter the shell boreholes. A similar result was found with respect to variation of pH values (Supplementary Fig. 18). Previous studies in euendolithic cyanobacterium *Mastigocoleus testarum*^28^ showed that the released Ca^2+^ from the carbocite passively diffused into the apical cell, moved thereafter from cell to cell, and was finally pumped into the liquid medium by the distal cell through a P-type calcium ATPase. In *Pyropia*, two genes encoding P-type calcium ATPase were identified and both of them exhibited high expression in the conchocelis. We added vanadate, the specific inhibitor of P-type cation-transporting ATPase, into the growth medium and found that Ca^2+^ extrusion was strongly inhibited, implying a pivotal role for the transporters in this process (Fig. 4c). These results suggest that the extracellular CAs produced by the conchocelis were capable of converting shell CaCO_3_ into HCO_3_^−^, which was subsequently taken up by the alga to support photosynthetic growth.

To corroborate this hypothesis, we grew free conchocelis with ^13^C-labeled CaCO_3_ and analyzed the abundance of ^13^C in organic acids in the tricarboxylic acid (TCA) cycle. A total of 39% of total carbon was labeled with ^13^C isotopes in this experiment (LABEL), whereas the abundance in conchocelis cells co-cultured with ^12^C-CaCO_3_ (CTRL) was 1%. Specifically, CTRL cells produced 0.4–1.7% of ^13^C isotopologs of succinic acid, fumaric acid, 2-ketoglutaric acid, oxaloacetic acid, malic acid, and citric acid. Between 2.1 and 4.9% of ^13^C was detected in the LABEL cells, which is far greater than the natural abundance of ^13^C (Fig. 4d). These results demonstrate that conchocelis cells assimilate available ^13^C-labeled CaCO_3_ that may be made available by excreted CAs that transform carbonate into bicarbonate.

### Role of horizontal gene transfer (HGT) in *P. yezoensis* evolution

**Fig. 5:**
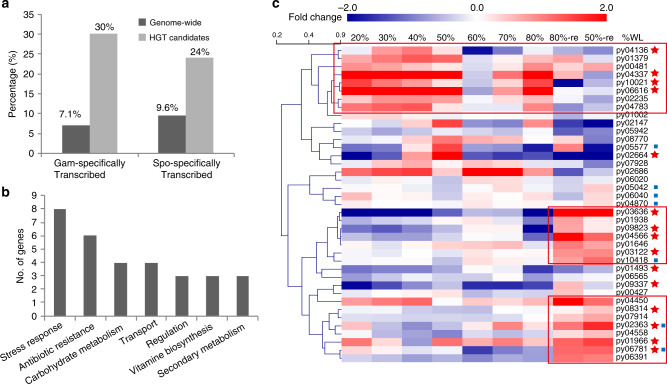
HGT candidates identified in the *P. yezoensis* nuclear genome. **a** The percentage of differentially expressed gam-specifically transcribed and spo-specifically transcribed genes that are HGT candidates when compared to overall gene expression values. **b** Functional categorization of HGT candidates. Biological functions were defined by the associated Gene Ontology terms in biological process followed by manual curation. **c** The transcriptional patterns of HGT candidates under dehydration and rehydration stress. Heatmap was made as described in Fig. 2. Gam-specifically transcribed HGT genes are highlighted with red stars and genes involved in stress response are indicated as blue squares. Clusters that exhibited upregulation under dehydration or rehydration are in red lines.

To elucidate the potential impact of HGT on *P. yezoensis* gene origin and the evolution of its distinct life history, we did a comprehensive phylogenomic analysis of the predicted gene inventory. This approach identified 51 genes (ca. 0.04% of the inventory) that were acquired specifically by the Bangiales ancestor via HGT from prokaryotic donors (Dataset S2). These genes all formed well-supported monophyletic clades with various prokaryotic lineages. The most common roles played by HGT-derived genes include stress response (including antioxidant functions), antibiotic resistance, carbohydrate-related metabolism, and transport (Fig. 5a). Among them, seven genes formed expanded gene families, including SOD, CA, and *N*-acyl-D-glucosamine 2-epimerase genes, glycine *N*-methyltransferase gene, carbohydrate-active enzyme, and peroxiredoxin encoding genes. Interestingly, 52.9% of HGTs exhibited specific transcription in either of the two generations (29.4% in gametophyte and 23.5% in sporophyte), whereas the percentage of genome-wide expression was 16.7% (Fig. 5b). The enrichment of phase-specifically transcribed gene expression among HGT-derived genes suggests that these foreign sequences provide adaptive functions to *Pyropia* species. This hypothesis is supported by the significant variation in transcriptional level under osmotic stresses resulting from dehydration or rehydration. In total, 13/15 gam-specifically transcribed HGT-derived genes exhibited more than twofold change in at least two time points under stress (Fig. 5c).

HGT is an important mechanism for gaining access to novel genes and functions. Among eukaryotes, this principle is exemplified by the red algal genus *Galdieria* that has about 1% of the gene inventory in species arising through HGT^29^. Many of these novel genes confer an adaptive function in the hot spring habitats where these species live. The HGT origin of the SOD gene^30^ in *Pyropia* and the LOX gene in the kelp *Saccharina japonica*^31^ have previously been reported. Among the HGT candidates we found in *P. yezonensis*, eight genes are involved in stress responses. Combining the identification of HGT-derived CA gene for CCM, this indicates the important role of HGT in allowing *Pyropia* to thrive in its stressful habitat and overcome C_i_ limitation. This hypothesis is supported by the thallus-specific gene expression patterns of HGT-derived genes.

### High GC content in Bangiales genomes

An interesting feature of the *P. yezoensis* genome is the high overall GC content of 65%, which reaches 71% in coding regions. This attribute is shared with other Bangiophyceae including *P. haitanensis*^10^ (67.9%), *Bangia fuscopurpurea*^32^ (64%), and *P. umbilicalis*^9^ (66%), whereas the GC content ranges from 37 to 57% in other red algae (e.g., *C. crispus*^33^, *P. purpureum*^34^, *Galdieria sulphuraria*^29^, and *C. merolae*^35^; Supplementary Fig. 19a). No significant bias in GC content was observed in biological functions encoded by the *P. yezoensis* genome (Supplementary Fig. 19b). Comparison of the GC content between the two clades indicated an expansion of AT–GC transitions in the ancestor of Bangiophyceae. Among the 61 codons, 20 were enriched in Bangiales when compared to Florideophyceae (Supplementary Fig. 19c). Their GC content, on average was 80%; moreover, all of them had G or C at the third codon position.

To examine whether selective pressure is driving GC evolution in Bangiophyceae, we calculated the evolutionary rate of GC content change based on the 424 single-copy orthologous gene families identified by Orthofinder in Bangiales genomes and the other 15 genomes of photosynthetic eukaryotes. We classified amino acids into three groups based on GC content at the first two sites of a codon as high GC, low GC, or neutral. The substitution rate within groups (*d*_within_) and between groups (*d*_between_) in the two clades was compared. In both clades, mean *d*_within_ was greater than mean *d*_between,_ suggesting that amino acid changes trended toward a similar GC content_._ However, the mean ratio *d*_between_/*d*_within_ was significantly greater in the comparisons involving the Bangiales clade (0.789 ± 0.003) than those in Florideophyceae clades (0.646 ± 0.002; paired *t* test; *P* < 0.01). The different pattern of codon replacement for Bangiales suggested an increased rate of substitution toward GC-rich codons in this clade. For each single copy orthologous gene family in *Bangiales* and Florideophyceae, the ancestral sequence was constructed through an optimized non-homogeneous model. Their average GC content was estimated as 53.85% (Supplementary Fig. 19a). A significantly greater frequency of A/T to G/C changes was found in Bangiales (126,126) compared with the Florideophyceae (27,585; paired *t* test: *P* < 0.05) while the frequency of G/C to A/T changes (42,760) was nearly equal in the two clades. The number of A/T to G/C changes in third nucleotide of the codons was about fourfold greater in Bangiales (67,313) than in Florideophyceae (17,809). These results demonstrate an excess of A/T → G/C substitutions, in particular GC_3_, among Bangiales.

## Discussion

A variety of evidence from physiology, evolutionary biology, paleontology, geosciences, and molecular genetics provides insights into the co-evolution of the Earth’s atmosphere, geosphere, and biology. The advent of oxygenic photosynthesis around 2.4 Ga increased atmospheric and surface ocean O_2_ levels and reduced atmospheric CO_2_^36,37^. Atmospheric CO_2_ concentrations reached a maximum of 0.5% around 500 Mya and then experienced a long downward trend, reaching a level that is unable to saturate RuBisCO about ~286 Mya. This long-term decline in CO_2_ concentration has shaped the evolutionary history of photosynthetic eukaryotes, resulting in several fundamental adaptations that impact kinetic properties of RuBisCO and the CCM^38–40^. The timescale of Bangiales evolution suggests that its ancestor experienced long-term CO_2_ limitation, which may have been a major driving force in this lineage. Bangiales displayed an alternation of two heteromorphic generations in distinctive habitats. The morphological differences and the resulting ecological advantages in their alternative life cycles might be a secondary adaptation to environmental stresses in the evolutionary history of photosynthetic organisms^41^.

Analyses of the *P. yezoensis* genome reveal the important role of carbon limitation in Bangiales evolution. The *Pyropia* lineage has adapted to C_i_ limitation through the large-scale duplication of CA genes and enhancement of its antioxidation system with increased gene copies of SOD, LOX, TRX, and TYR. HGTs from bacteria and local gene duplication (indicated by αCA8 and αCA9) partially explain gene expansion in Bangiales. These gene families tend to show phase-specific expression, implying that they may be an adaptive response to the different growth conditions in the heteromorphic life history. For the thallus phase, inhabiting the upper intertidal zone, fluctuating stresses during emersion lead to intracellular over-production of ROS, which would then damage cellular structures and macromolecules^42^. The expanded gene repertoire of the antioxidative system and their upregulation in the thallus phase strongly support the essential roles of these genes in dealing with environmental stresses during emersion.

Based on the gene expression patterns of CA isoforms and related bicarbonate transporters in the two life history phases, as well as their distinct subcellular localizations, we propose a model for the CCM in *Pyropia*. Multiple gam-specifically transcribed CAs and related transporters were likely to contribute to carbon acquisition in the thallus. Their potential functions in response to CO_2_ fluctuation during emersion in the upper intertidal zone is supported by elevated transcription during dehydration. Moreover, the transcription of CAs is apparently induced at different time points of dehydration as well as rehydration, indicating diverse roles of individual CAs. Genetic manipulation is needed for each CA isoform to determine their physiological function, as well as greater knowledge about their subcellular localization, to confirm these observations.

The conchocelis utilizes the carbon available in mollusk shell by promoting dissolution of CaCO_3_. This phenomenon of mineral-sourced autotrophy has been previously documented in the cyanobacterium *Mastigocoleus testarum*^28^. In *Mastigocoleus*, the dissolution of CaCO_3_ starts from a passive diffusion of Ca^2+^ into the apical cell. The lowered of Ca^2+^ concentration in the interstitial microenvironment shifts the ionic equilibrium toward dissolution. Our observation of increased Ca^2+^ concentration and pH in the external medium in cultures of *Pyropia* conchocelis, as well as the central role of P-type cation transporters in transporting Ca^2+^ into distal cells, support this model. Moreover, we found that the extracellular CAs, in particular the two specifically transcribed in the conchocelis, play essential roles in this process. Therefore, we speculate that, in addition to the spontaneous Ca^2+^ ionization as proposed in the *Mastigocoleus* model, CA excreted by the conchocelis cell may promote the dissolution of Ca^2+^ through generating protons that attack CaCO_3_ (Fig. 4e), as proposed in some limestone-dissolving bacteria^26,27^. Two bicarbonates are produced in this reaction, one from CO_2_ and one from CaCO_3_. These might immediately enter the cell through bicarbonate transporters and their rapid elimination in the microenvironment would push the chemical reaction in this direction. The import of Ca^2+^ is coupled with the excretion of two protons to maintain charge. One proton would combine with ionized CO_3_^2−^ to form bicarbonate. The fate of bicarbonate could either be entering the cell and being converted to CO_2_ by intracellular CA or being converted directly into CO_2_ with another excreted proton by the way of extracellular CA. The dissolution of CaCO_3_ might also play a role in conchocelis penetration into the shell matrix. Based on our results, the utilization of shell CaCO_3_ as a carbon source, with the participation of extracellular CA, P-type ATPase, and the subsequent intracellular CCM components expressed in the conchocelis, is strongly supported. However, the contributions of CaCO_3_ to the total C_i_ uptake in the conchocelis, the physiological function of CAs, and calcium transporters remain to be determined.

In marine bacteria with divergent GC content, carbon limitation may be an important factor driving GC content evolution because the GC pair requires one less carbon atom than AT^43–45^. In the evolutionary history of the Bangiales, their common ancestor has experienced long-term carbon limitation resulting from the sharp decline in atmospheric CO_2_ in the Permo-Carboniferous era. Therefore, we speculate that, as in marine bacteria, the preference to use carbon-saving GC bases might have been an adaptive strategy responding to ancient carbon limitation. Carbon limitation also has left imprints on amino acid usage in Bangiales. We calculated the average content of each amino acid in the single copy orthologous genes of Bangiales and Florideophyceae genomes. Among the 19 amino acids with carbon in side chains, Gly and Ala containing the least carbon atoms (2 and 3, respectively) were enriched in Bangiales when compared with Florideophyceae (paired *t* test; *P* < 0.01), whereas the content of amino acids with 5, 6, and even more carbons in the two clades were equivalent (Supplementary Table 6). The strong bias toward a reduced frequency of carbon-containing amino acids in Bangiales genomes is consistent with (but does not prove) the hypothesis of carbon limitation driving the evolution of Bangiales. It is clear that other factors may impact GC content such as genome size increase driven by transposon expansion (e.g., in grasses^46^) and high levels of intra-genic recombination (i.e., GC-based gene conversion) that can act independently from selection to create codon usage bias^47^.

## Methods

### Genome sequencing and assembly

To avoid contamination of surface bacteria and organelle DNA, we first isolated nuclei from de-contaminated homozygous *Pyropia* thalli and then extracted genomic DNA to construct sequencing libraries (Supplementary Methods 1–3, Supplementary Figs. 20 and 21). Long read sequencing data from Pac-Bio RSII platform were collected and used in initial assembly via RS_HGAP_Assembly.3 protocol. Mate pair Illumina reads with insert size at 5 kbp were also used to generate scaffolds using SSPACE^48^. Gap-closing through PBJelly2^49^ and genome polishing through Quiver algorithm from SMRT Analysis v2.3.0^50^ were then done (Supplementary Method 4). Scaffolds in the primary assembly were then subject to a five-step de-contamination pipeline to detect potential bacterial sequences (Supplementary Method 5). Twenty scaffolds were filtered out in this way. To improve the assembly, optical maps of the BioNano system were generated and used for scaffolding, followed by a proximity-guided assembly based on Hi-C data. Bacterial sequences were manually investigated and removed from the final assembly.

For transcriptome sequencing, full-length cDNA sequencing was first done with the Pac-Bio SMART sequencing platform using an RNA mixture from multiple development stages and stress treatments to cover as many transcripts as possible (Supplementary Method 6). Differential gene expression sequencing for gametophyte thalli and sporophyte conchocelis, as well as thalli, under desiccated and re-hydrated osmotic stresses were performed on the Illumina GAIIx platform (Supplementary Methods 7 and 8). The relative transcriptional abundances of each gene model under various conditions were calculated as FPKM values and normalized using the cufflink program^51^.

### Genome annotation

Repeat elements were identified using Repeatmodeler and Tandem Repeats Finder^52,53^. De novo prediction via AUGUSTUS^54^, homology searches, and RNA-aided prediction via PASA^55^ followed by EVM integration was used to predict protein-coding gene models from the repeat-masked genome. The gene models were then functionally annotated using the NR, InterPro, GO, KOG, KEGG, CAZyme, and CDD databases (Supplementary Methods 9–11). Subcellular localization of *Pyropia* proteins were predicted using PredAlgo, which is a multi-subcellular localization prediction tool designed for algae^56^. Trans-membrane helixes were predicted using TMHMM (v2.0)^57^ available at http://www.cbs.dtu.dk/services/TMHMM/.

### Evolutionary analysis of nucleotide composition in red algae

We implemented the strategy described in Luo et al.^43^ to infer the evolution of nucleotide composition in red algal genomes. Based on the phylogenetic tree built from single-copy orthologous genes, we first estimated substitution parameters of the alignment sequence with the Bio++ maximum-likelihood algorithm using the non-homogeneous model as implemented in the BppML programs from Bio++ suite to infer ancestral states of a continuous character^58^. Then the ancestral sequences for each node of the tree were computed using the BppAncestor program^58^. Conservative and radical amino acid replacements were defined as amino acid substitutions within and between groups, respectively. Rates of conservative (dC) and radical (dR) replacements, that is, the number of conservative nonsynonymous nucleotide substitutions per conservative nonsynonymous site and the number of radical nonsynonymous nucleotide substitutions per radical nonsynonymous site, between two orthologous genes were calculated using the HON-new software^59^. The dR and dC substitution of each node were computed by classifying amino acids into three groups according to GC content at the first two sites of a codon (most codons encoding one amino acid may differ in the third site): high G+C, low G+C, and neutral.

### Gene expanding and contraction analysis

To examine genome divergence among red algae, we carried out a phylogenetic analysis using the *P*. *yezoensis* and five other red algal genomes as well as model algal genomes to identify orthologous genes using OrthoFinder^60^. Single-copy orthologous groups were identified and used to construct a phylogenetic tree with MrBayes3.2^61^. Divergence time was calculated using the Bayesian relaxed molecular clock approach with the divergence time of the Bangiaceae and Florideophyceae set as the standard^62,63^. The number of genes in each OG were statistically compared among red algal genomes using the CAFE program^64^ (Supplementary Method 12). HGT candidates were inferred following phylogenetic analysis described for the genome of *Paulinella chromatophora*^65^, with detailed information included in Supplementary Method 13.

### Detection of the variation of Ca^2+^ concentration and pH value

Three conchocelis-dwelling shells with similar sizes were cultured in a sterilized flask containing 500 ml artificial sea water (without Ca^2+^) and Provasoli’s enrichment medium (growth medium) without aeration. A 500× stock solution of acetazolamide was prepared in boiled water. One milliliter of the stock was added into culture medium to inhibit the activity of external CA (final Conc. = 100 μM, CA inhibitory medium). P-type ATPase inhibitory medium was prepared in the same way with sodium vanadate (final Conc. = 100 μM). Three biological replicates were done for each group. Provasoli’s enrichment medium was refreshed every 6 days. For Ca^2**+**^ and pH detection, 30–50 μl medium in the vicinity of shells was aspirated using a syringe with minimal disturbance to the medium on the 0th, 3rd, 6th, 9th, 12th, and 15th day. Ca^2+^ concentration was tested by an atomic absorption spectrophotometer. pH values were measured at the same time points by a pH meter.

### Stable isotope-labeling experiment

For the ^13^C-labeling experiment, 0.3 g free conchocelis was cultured in 200 ml artificial sea water with 0.3 g ^13^C-labeled CaCO_3_ (99 atom%, Sigma-Aldrich), in 20 ± 1 °C under 50–60 μmol photons m^−2^ s^−1^ (12:12 light:dark cycle) with three rounds of gentle shaking every day. Control samples of equal weight were co-cultured with ^12^C-CaCO_3_. Three biological replicates were done with these experiments. After 20 days of cultivation, the abundance of ^13^C atom in the organic acids related to the TCA cycle were quantitatively analyzed using gas chromatography–mass spectrometry based on the retention time, electron ionization mass spectrum of corresponding standards, and matching to the NIST library, as described in Mulat et al.^66^.

Information about the methodology used in transient expression of CA::GFP fusion proteins in tobacco and enzymatic activity assay of extracellular CA in conchocelis are included in Supplementary Methods 14 and 15, respectively.

### Reporting summary

Further information on research design is available in the Nature Research Reporting Summary linked to this article.

## Supplementary information

Supplementary Information

Peer Review

Reporting Summary

Source Data file

## Data Availability

Data supporting the findings of this work are available within the paper and its Supplementary Information files. A reporting summary for this article is available as a Supplementary Information file. The datasets generated and analyzed during the current study are available from the corresponding author upon request. Whole-genome sequencing data of *P. yezoensis* were deposited in NCBI under the BioProject PRJNA589917. Genomic sequencing raw data were deposited under SRR10480798 (Illumina platform), SRR10484745 (Hi-C platform), and SRR10489006–SRR10489010 (PacBio platform). The final assembly is available at DDBJ/ENA/GenBank under the accession number WMLA00000000. The version described in this paper is version WMLA01000000. The transcriptome sequencing data were deposited under SRR10502194–SRR10502223 (osmotic stresses), SRR10527930–SRR10527937 (two life cycle stages), and SRR10502264–SRR10502266 (full-length cDNA sequencing using the PacBio platform). The source data underlying Fig. 4c, d, as well as Supplementary Fig. 18 are provided as a Source data file.
