# Supplementary material for: Pyropia yezoensis genome reveals diverse mechanisms of carbon acquisition in the intertidal environment
Source: Nat Commun. 2020 Aug 12;11:4028. doi: 10.1038/s41467-020-17689-1 (PMC7423979; doi:10.1038/s41467-020-17689-1)
Supplement: Supplementary file 1 — Supplementary Information [file 41467_2020_17689_MOESM1_ESM.pdf]

Pyropia yezoensis genome reveals diverse mechanisms of carbon acquisition in the intertidal environment

Wang *et al.*

Supplementary Note 1. Repeat elements in *P. yezoensis* genome

Nearly one-half of the *P. yezoensis* genome (48.0%) is predicted to be repeat elements (Supplementary Table 4). The most prominent repeat element is interspersed repeats (90.6%, mainly are Class I and LTR retrotransposons), whereas the remaining 9.4% are tandem repeats (included microsatellite, minisatellite and satellite). We used LTRharvest to predict intact LTR sequences in the two *Pyropia* genomes as well as the other four red algal genomes. Apparently, *P. yezoensis* harbors much more intact LTR sequences (1641) while the number in *Porphyridium purpureum*, *Cyanidioschyzon merolae*, *Galdieria sulphuraria*, *Chondrus crispus*, and *Porphyra umbilicalis* genomes are only 0, 36, 3, 1275, and 862, respectively. Moreover, the number of intact LTR correlates well to genome size (the correlation coefficient R^2 was 0.9437), suggesting the composition of LTRs accounting mostly for genome size amplification in *Pyropia* (Supplementary Fig. 5), similar to the investigation in the *Gracilariopsis* genome[1]. Host genomes developed two mechanisms to control the TE activities and thus attenuate the negative effects of TEs on the structure and function by causing DNA double-strand breaks, the post-transcriptional gene silencing (PTGS) and transcriptional gene silencing (TGS). However, in contrast to *Gracilariopsis,* the near absence of DNA methylation of cytosines excludes the potential involvement of DNA methylation in TE regulation.

Supplementary Note 2. Genome synteny in Bangiales genome

To infer the evolution of genomic structure in Bangiales genomes, we used MCScanX [2] software to scan the genomes of *Pyropia yezoensis*, *Pyropia haitanensis* [3] and *Porphyra umbilicalis* [4] in order to identify putative homologous chromosomal regions (Supplementary Fig. 7). Large amounts of long syntenic regions were identified between the two *Pyropia* species whereas only few and short between *P. yezoensis* and *Po. umbilicalis*. The syntenic difference between *Pyropia* and *Porphyra* implied that large amounts of structural rearrangement happened after the divergence of the two genera, although we didn't exclude the possibility of incomplete assembly of *Po. umbilicalis* genome. We then investigated the synteny between CA homologs.

CA genes were apparently dispersed in the three chromosomes in *P. yezoensis*, yet exhibited high conservation with their *P. haitanensis* counterparts in both protein sequences and synteny. A total of 16 *P.yezoensis* CA genes showed syntenic relationship with those in *P. haitanensis* (Supplementary Fig. 12). Interestingly, αCA8 and αCA9 are adjacent to each other in scaffold S2, with a 3.7kb interval. Their protein sequences were highly similar (Identity= 95%), except for the beginning 70 AAs. Orthologs of the two CA genes were also found localized closely in *P. haitanenesis genome*. However, *Po. umbilicalis* orthologs of αCA8 and αCA9 were localized in two contigs, KV919297.1 and KV918987.1 respectively. Each locus was at the end of corresponding contig. Therefore it's possible that the two contigs should be assembled together. Moreover, αCA9 was transcriptionally active in *Pyropia* conchocelis, while transcripts of αCA8 was not detected. Thus we postulate that αCA8 and αCA9 might originate from a gene duplication event happening in the ancestor of the two genera, following with transcriptional silence of αCA8 in *P. yezoensis* due to unknown reasons.

Supplementary Note 3. Expanded OGs in the *P. yezoensis* genome

Among the orthologous relationships built in red lineage, 169 OGs containing 1337 genes in total were specifically expanded in the *Pyropia yezoensis* genome. They mostly encode proteins involved in macromolecular modification, particularly in protein modification by ubiquitin and small protein conjugation/removal, as well as the protein folding. The gene number of HSP70 in the *Pyropia* genome has a sharp increase compared to the other red algae. Protein modification and folding are related to the regulation of protein recycling and activity. The expanded genome apparatus in protein-level regulation might result in immediate adjustment of physiological activities in responding to extracellular stimulation.

Supplementary Note 4. Expanded gene families that are related to anti-oxidant functions

In addition to SODs, generation-specific transcription and co-expression of the gam-specifically-transcribed counterparts under osmotic stresses were also observed in other expanded gene families. Eight lipoxygenase (LOX) genes were identified in *P. yezoensis* genome, while there's only one in *Chondrus* and none in unicellular red algae (Fig. 2a). Besides of the one with specific transcription in conchocelis and two with very low expression in both generations, all of the other five have particularly higher transcriptional level in thallus than in conchocelis (Supplementary Fig. 10a). When under osmotic stress, they synchronously have up-regulated expression when rehydrated after extreme water loss (Supplementary Fig. 10b).

Tyrosinases are a type of polyphenol oxidases (PPOs) catalyzing the hydroxylation and oxidation of monophenols (e.g., tyrosine) to *o*-diphenols and the corresponding *o*-quinones. They are widespread among plants, fungi, and bacteria and involved in plural physiological functions, such as the resistance to wound, pathogens and herbivore in plants, as well as the pigment formation in response to UV radiation in animals (e.g. melanin)[5]. Though rarely found in some chlorophyte (including green algae and several plants) and other red algae, 12 tyrosinase genes were identified in *P. yezoensis*, and 14 in the *P. umbilicalis* genome (Fig. 2a). A phylogenetic reconstruction was generated with plant PPO proteins as well as bacterial and animal tyrosinases. Interestingly, red algal tyrosinase proteins formed a monophyletic group. *Pyropia* paralogs shared high similarity in protein sequences (50-72%). In addition to the one spo-specifically-transcribed tyrosinase and five constitute ones, there are six gam-specificall-transcribed tyrosinase genes and five out of them have synchronous up-regulation at the recovering stage with re-hydration stage despite of their slight and multidirectional variation under dehydration conditions (Supplementary Fig. 11).

Another expanded gene family with anti-oxidative function in the *Pyropia* genome was catalase. There are three catalase genes in *Pyropia* while 2, 1, and 1 found in *Chondrus*, *Porphyridium*, and *Cyanidioschyzon* respectively. In addition to the one

spo-specifically-transcribed CAT gene, the other two have transcriptional activity in both generations and exhibited up-regulation under osmotic stresses.

Supplementary Note 5. C_i transporters in *P. yezoensis* genome

Unlike terrestrial plants, the dominant source of C_i in aquatic algae is HCO_3^- because the diffusion of CO_2 into the cell is extremely slow [6, 7]. Therefore, HCO_3^- uptake, as well as intracellular transport determines the efficiency of the CCM. Among the proteins involved in C_i transport in *C. reinhardtii* [8], including HLA3, LCI1, CCP1, CCP2, only the formate-nitrite transporter LCIA has a putative homolog in the *nori* genome which contains a putative plastid targeting sequence. We searched for LCIA homologs in algal genomes and studied their evolutionary history using phylogenetics. Red algal LCIAs are clustered together and associated with homologs from algae contain secondarily derived red algal plastids (e.g., diatoms, Supplementary Fig. 17a). In addition to LCIA, we find three genes encoding HCO_3^- transporters that are also shared with diatoms (Supplementary Fig. 17b). All of these contain multiple transmembrane helixes facilitating their integration into the plasma or plastid membrane. Two of these proteins contain plastid targeting peptides indicating their localization to the inner layer of the plastid to allow uptake of cytosolic HCO_3^-. The last one showed high phylogenetic affinity to diatom SLC4 proteins that is experimentally confirmed to transport C_i[9]), thus may anchor in the cell membrane to transport extracellular HCO_3^- into the cytosol. All of the LCIA and SLC4 isoforms were expressed in both gametophyte and sporophyte (Supplementary Fig. 17c) The close evolutionary relationship of LCIAs homologs in red algae and

diatom, as well as the co-existence of multiple SLC4-type bicarbonate transporters compared to green algae, suggested the cognate origin of red algal and diatom CCM and the diatom might have acquired the CCM apparatus from the engulfed red alga during secondary endosymbiosis.

Supplementary Method 1. Preparing of homozygous *P. yezoensis* and cultivation

Gametophytes of *P. yezoensis* were originally collected in 2010 in Rizhao, Shandong Province, China. After rinsing the material with sterilized seawater several times to remove impurities, it was cultured at 10 ± 1 °C under 50-60 μmol photons m^{-2} s^{-1} (12:12 light:dark cycle) with constant aeration, by renewing the sterilized seawater supplemented with Provasoli's enrichment medium [10] every 3 days. One single somatic cell was enzymatically isolated from the gametophyte and developed into a thallus which subsequently self-fertilized into the pure line RZ.

Free conchocelis was cultured with the same medium to thallus, albeit at 20 ± 1 °C under 20-30 μmol photons m^{-2} s^{-1}, without any aeration. To make shell-dwelling conchocelis, 0.1g free conchocelis in 1L medium were smashed in a blender for five seconds and then incubated with 100 mollusk shells in dark condition at 20 ± 1 °C. 24 hours later, the shell-dwelling conchocelis were cultivated at the same condition with free conchocelis, with medium refreshed every two weeks.

Supplementary Method 2. DNA sample preparation for sequencing

To remove surface bacteria, *P.yezoensis* thallus were harvested and mixed with quartz sands. A physical vibration was performed in homogenizer (Precellys 24), following with several times of washing to remove the polysaccharide and bacteria from the surface of gametophytes.

Nucleis were isolated from the above clean blades as described in Zhang *et al.* and then dyed with Carbol fuchsin and DAPI to check the intactness [11]. To remove the contamination of plastid DNA, we carried out pulsed-field gel electrophoresis to isolate nuclear DNA as follows. The *P. yezoensis* nucleis were mixed with 1% low melting-point agarose (Sigma) at 50°C, and aliquoted into the plug molds and solidified 4°C for 1h. The plugs were incubated with digestion bufer (0.5 mol/L EDTA buffer with pH at 9.0-9.3, 1% sodium lauryl sateosirte and 1g/L proteinase K) at 50 °C for 48 hours. After a series wash by 0.5 mol/L (pH=9.0) and 0.05 mol/L

(pH=8.0) EDTA buffers, the plugs were loaded into 1% low melting-point agarose to perform PFGE. The upper main band representing nuclear DNA were excised and the DNA were then retrieved through digestion using low melting point agarose enzymes.

Supplementary Method 3. DNA libraries construction and sequencing

For Illumina library construction and sequencing, paired-end library with insert size of 500 bp was constructed following the standard Illumina protocol and then loaded on Illumina HiSeq 2000 platform for sequencing. In addition, a mate pair library with insert size of 5kb was further constructed and sequenced. After filtering out low quality reads and adaptor sequences, reads that successfully aligned to plastid (KC517072) and mitochondrial (NC017837.1) genomes of *P. yezoensis* were also removed. The resulted clean reads were then used to calculate genome size and heterozygosity through 17-mer distribution.

For SMRT library construction and sequencing, SMRTbell genomic library (>20 kbp in length) was constructed using 5μg of high-quality nuclear DNA and loaded on RSII platform (P6C4 chemistry) for sequencing. The raw reads were then trimmed for low quality sequences and the organelles sequences were removed by BLASR [12]. Subsequently, clean data (~76 X genome depth) was used for following assembly.

Additionally, 4 μg purified high molecular weight genome DNA was digested by BspQI, then was labeled by fluorescence and counterstained for constructing Bionano librariy. For the Hi-C library, chromatin was fixed in placewith form aldehyde in the nucleus, and then the fixed DNA was extracted and digested with MboI. Sticky ends were biotinylated and proximity ligated to form chimeric junctions that were enriched for and then physically sheared to a size of 300-500 bp. Chimeric fragments representing the original cross-linked long-distance physical interactions were then processed into paired-end sequencing libraries.

Supplementary Method 4. Genome assembly and assessment

For genome assembly, RS_HGAP_Assembly.3 protocol in SMRT analysis v2.3.0 [13] was used to assemble the nuclear genome of *P. yezoensis* based on the subreads from PacBio [14]. For HGAP, the following main parameters were used: minimum subread length = 500 bp, minimum polymerase read quality = 0.80, minimum polymerase read length = 500 bp, minimum seed read length = 6,000 bp, estimated genome size = 100 Mb, target genome coverage = 25, overlap error rate = 0.06, minimum overlap = 40 bp, overlap k-mer = 14. Other parameters were used as default settings. Subsequently, the mate pair reads were used to construct scaffolds using SSPACE [15]. Besides, PacBio long reads were mapped to the scaffolds using BLASR. Thereafter, PBJelly2 was used to fill up the gaps among the scaffolds with default parameters [16]. Then, Quiver was used to polish the accurate the whole genome at base level. Scaffolding of the contigs with optical mapping was performed using the Irys optical mapping technology (BioNano Genomics). Labeled single molecules above 150 kb in size were used to produce single-molecule maps and assemble maps into a genome map by the IrysView (BioNano Genomics, https://bionanogenomics.com/support/software-downloads/) software package. Scaffolding was further performed using Hi-C technology. The paired-end reads were uniquely mapped onto the draft assembly contigs. Three chromosome clusters were generated. Further scaffolding within chromosome cluster each was done using 3D-DNA software (editor_repeat_coverage=50).

The quality of assembly was further assessed through the following steps. *First*, the genome size is similar to the size calculated by K-mer using Illumina PE reads (10.4 Gb); *Second*, the Illumina PE reads were used to aligne to the current assembly with 2 mismatches allowed; *Third*, Pac-Bio isoforms were aligned to current assembly by bowtie2 and BLAT respectively; Forth, the number of predicted genes (see below) are comparable to the numbers in published genomes of both unicellular and multicellular algae (ex. *Chondrus*, *Chlamydomonas* etc.). The gene models predicted from the genome assembly was further mapped to the the single copy orthologs of Eukaryota in BUSCO database [17]. Similar analysis was done for the other 15

published algal genomes and three plant genomes. The percentage of mapped orthologs Eukaryota in each genome was calculated to further verify the completeness.

Supplementary Method 5. Remove potential bacterial contamination

To remove the potential contamination of bacterial sequences in the current assembly, we employed a five-step filtering pipeline. 1) Scaffolds were blasted against NT database. Then, MEGAN 6 [18] was used to analyze the distribution of the aligned species and search for bacterial sequences. 56 scaffolds were found to be contaminated bacterial sequences from this "nt" step. 2) We cut each scaffold into 100bp overlapping 1Kb windows and blasted them against the NT database using blastn. The blast results were further analyzed using MEGAN [18] to search for bacterial hits. If >60% of windows in a scaffold had best hits as bacterial sequences with identity >70%, this scaffold was picked out as possible bacterial contamination. In this "nt-split" step, we got totally 26 scaffolds. 3) The 1Kb windows were blasted against NR database using blastx. 35 scaffolds having >60% of windows mapped on bacterial proteins were searched out in this "nr-split" step. 4) The transcript data generated from both Illumina and Pacbio platforms were mapped genome using Blat[19]. 85 scaffolds didn't have any cDNA support. 5) Sequencing depth of each scaffold was calculated using the data generated from Illumina platform. 149 scaffolds had a sequencing depth less than 5. In the end, 20 scaffolds that were searched out in all of the above five steps were considered to be bacterial contaminations and removed from the final genome (Supplementary Fig. 1).

Supplementary Method 6. Constructing the full-length transcriptome sequencing library

P.yezoensis thallus that was subjected to different stress treatment (osmotic pressure, temperature, illumination etc) was collected in order to cover as many genes as possible. Total RNAs were isolated from each treatment by RNesay ® Plant Mini (Cot.No.74904, Qiagen) following the manufacturer’s protocols and then treated with

RNAase free DNase I (Omega) to remove genomic DNA contamination. The purity and concentration of the RNAs were detected using the NanoPhotometer® spectrophotometer (IMPLEN, CA, USA) and the integrity was determined by the Agilent 2100 Bioanalyzer (Agilent, Santa Clara, CA). Equal amounts of RNAs from each treatment were mixed together to construct the full length cDNA library for SMART sequencing. First-strand cDNA was synthesized using the SMARTer PCR cDNA Synthesis Kit (#634926; Clontech, http://www.clontech.com). The tailing by SMARTScribe™ Reverse Transcriptase could switch the same adaptor primer on the 3' and 5' ends of the poly(A) RNA using CDS Primer IIA (5' -AAGCAGTGGTATCAACGCAGAGTACT (30) N–1N-3') and SMARTer IIA Oligonucleotide (5' -AAGCAGTGGTATCAACGCAGAGTACXXXXX-3'). Next, second-strand cDNA synthesis was performed using Phusion High-Fidelity DNA Polymerase (#M0530; NEB, http://www.neb.com) with 5' PCR Primer IIA (5' -AAGCAGTGGTATCAACGCAGAGTAC-3'). To avoid over-amplification of small fragments, we optimized the amplification cycle as 14 by a preliminary test. Purified cDNA was normalized using the Trimmer-2 cDNA Normalization Kit (#NK003; Evrogen, http://www.evrogen.com)and then amplified using the 5' PCR Primer IIA with 18 cycles. Agarose gel-based size selection was performed using the SYBR Safe DNA Gel Stain and blue light system to avoid DNA damage. Then, three gel fractions, containing fragments >3, 2-3, 1-2kb, were collected and purified using the QIAquick Gel Extraction Kit. The extracted products were amplified using the 5' Primer IIAand purified using 0.5×AMPure beads (#A63880; Beckman, http://www.beckmancoulter.com).

Supplementary Method 7. Quantify the transcriptional variations *Pyropia* genes in two life phases

Gametophyte thalli and sporophyte conchocelis were collected and used to isolate total RNAs as described above. Four biological replicates were done for each life phase. The mRNA was first purified from total RNA by Dynabeads® Oligo (dT)

(Life，America) and then was fragmented. The double-stranded cDNAs were then synthesized using transcriptase and random hexamer primers. The sequencing library was constructed using the NEBNext® UltraTM RNA Library Prep Kit (NEB, America). Subsequently, the library prepared was loaded on Illumina HiSeq2000 platform for a PE 2×100bp sequencing.

After sequencing on an Illumina Hiseq 2000 platform, the generated raw data were filtered to remove low quality reads, adapter sequences and reads containing N. Then, the clean data were aligned to the assembly genome of using TopHat (v2.0.12) (http://ccb.jhu.edu/software/tophat/index.shtml). The mRNAs expression was calculated as Reads Per Kilobase per Millions reads (RPKM). The differential expression of mRNAs was performed using the DESeq2 R package (1.10.1) [20]. The criterion of adjusted p-value <0.05 was used to identify differentially expressed genes. The transcriptome sequencing library for Illumina GAIIx was prepared strictly following Illumina's instructions.

Supplementary Method 8. Monitoring transcriptional dynamics during the full course of dehydration and rehydration in *Pyropia*

Algal thalli were subjected to the desiccation treatment in the incubator with constant temperature and light, same as the above culture condition: First, the algal thalli were taken out of the medium and the surface water was removed using an autoclaved gauze. Each thallus was weighted, spread on a glass dish and then exposed to air in the incubator. The sample was collected when its water loss based on weight was 20%, 30%, 40%, 50%, 60%, 70%, or 80%, as described in [21]. Some thalli with 50% or 80% water loss were put back into the culture medium for 30 minutes of rehydration and then collected as rehydrated samples. Each treatment had three biological replicates. Collected algal samples were frozen immediately in liquid nitrogen. Fresh thalli collected directly from medium culture were designated control samples for the desiccated ones. Samples with 50% and 80% of water loss were used as control for the 50%- and 80%-rehydrated samples respectively. RNA isolation and library construction, Illumina sequencing and FPKM calling were done for each

sample, as described above.

Supplementary Method 9. Prediction of protein-coding genes

A combination of *de novo* prediction, homology searches and RNA-aided prediction was used. *De novo* prediction was performed using AUGUSTUS with the full-length transcriptome sequencing data as training set [22]. For homology searches, a database containing protein sequences from 10 organisms (*Arabidopsis thaliana*, *Chlamydomonas reinhardtii*, *Chondrus crispus*, *Cyanidioschyzon merolae*, *Cyanophora paradoxa*, *Ectocarpus siliculosus*, *Phaeodactylum tricornutum*, *Porphyridium purpureum*, *P.yezoensis*, *Saccharina japonica*) was used. Unigene sequences of *P. yezoensis* were also mapped back to the genome using BLAST and then assembled by PASA [23]. Finally, EVM was used to integrate these gene models from the above methods [23].

Supplementary Method 10. Functional annotation of protein-coding genes

To further detect the function of the protein-coding genes in *P. yezoensis*, the predicted protein sequences were annotated against several public databases (NR, Swiss-Prot, InterPro, GO, KOG, KEGG, CAZyme and Conserved Domains Database [24, 25, 26, 27, 28, 29, 30, 31, 32]. Sub-cellular localization of *Pyropia* proteins were predicted using PredAlgo program [33] which is a multi-subcellular localization prediction tool dedicated to algae. Trans-membrane helixes were predicted by TMHMM (v2.0) [34] available at http://www.cbs.dtu.dk/services/TMHMM/.

Supplementary Method 11. Predicting repeat element and non-coding RNAs

Repeatmodeler (http://www.repeatmasker.org/) was used to *de novo* identify repeat elements. The database generated was then loaded into Repeatmasker (version 3.2.7) [35]to mask repeat elements of *P. yezoensis* genome. We used LTR harvest to predict intact LTR sequences in the two *Pyropia* genomes as well as the other four red algal genomes. The tRNA sequences were detected using tRNAscan-SE-1.3 [36]. Rfam was used to predict rRNA, miRNA and snRNA [37].

Supplementary Method 12. Gene family expansion and contraction

To further examine the genome divergence and conservation among red algae, we carried out a phylogenetic analysis based on single-copy orthologous groups using the *P. yezoensis* genome and the other five red algal genomes as well as model algal genomes to build orthologous genes using OrthoFinder[38]. *Cyanophora paradoxa* was selected as the outgroup species. Single-copy orthologous groups were identified and were then used for to construct phylogenetic tree by MrBayes3.256 [39]. Four incrementally heated Markov chains were run for 10,000,000 metropolis- coupled Markov chain Monte Carlo (MCMC) generations for the concatenated data set, and runs were sampled every 1000th generation. The initial 10% were discarded as burn-in. A time-calibrated phylogeny was inferred using a relaxed molecular clock method as implemented in beast v.1.8.3 [40]. Besides, the number of genes in each OrthogGroup, combined with Pfam annotations were statistically compared among *C. merolae, P. purpureum, C.crispus, P.umbilicalis, P. haitensis, P.yezoensis* genomes using the CAFE program [41].

Supplementary Method 13: Identification of HGT candidates

Protein-coding genes in *P. yezoensis* genomes were each blasted against NCBI NR database by BlastP [42]. The e-value cut-off was set as 1e-5. All hits were recorded and pooled into a gene cluster. If more than 50 hits were found, the top 50 hits with the smallest e-values were kept. Amino acid sequences of these hits were extracted. A phylogenetic tree for each gene cluster was generated using MUSCLE [43] and PhyML [44]. The NCBI taxonomy database (http://www.ncbi.nlm.nih.gov/Taxonomy/) was used to assign taxonomy to each of the genes in the phylogenetic tree. For each phylogenetic tree, if more than 90% of hits, as well as the best hit, had different superkingdom taxonomies from that of *Pyropia*, then the query sequence was marked as a candidate HGT gene. In the end, we identified 343 HGT candidates. They were then subjected to the following validation.

Validation of the 343 HGT candidates in *P. yezoensis* was done using phylogenomics, as follows. Using BLASTp (*e*-value ≤ $1x10^{-10}$) [42] each sequence was queried against an in-house curated protein database comprising NCBI RefSeq release 59 together with predicted proteins of sequenced eukaryote genomes from the DOE Joint Genome Institute (jgi.doe.gov/), TBestDB [45], predicted proteins from the Marine Microbial Eukaryote Transcriptome Sequencing Project [46], and six-frame translations of eukaryote EST sequences from NCBI dbEST [47]. A maximum of 20 species from each phylum were selected in descending order of blast bitscore (to a maximum of 180 total species) from the results, and the respective protein sequences were aligned using MAFFT v. 7.2 ([48]; linsi option). Alignments were then subjected to phylogenetic analysis using IQ-TREE [49] with 2,000 ultrafast bootstrap replicates and automatic model selection. Genes of potential bacterial origin were manually inspected to identify the final list of 51 candidates as described in Nowack *et al.* [50], whereby we screened the data for trees with *P. yezoensis* + prokaryote monophyly with bootstrap support ≥70%.

Supplementary Method 14. Transient expression in tobacco

0.5g thalli and 0.5g conchocelis were wiped and mixed for RNA extraction. Total RNA was extracted following the instruction of the M5 HiPer Plant RNeasy kit (Mei5 Bioservices) and reversely transcribed by HiperScript III reverse Transcriptase (Mei5 Bioservices) using oligo (dT) as primer. The generated cDNA was then used as template to amplify CA genes. Specific primers for each CA gene were listed in Supplementary Table 6. For each CA gene, the coding sequence was amplified without the stop codon and fused in frame to the upstream of GFP in the modified binary vector pCAMBIA 1300-35S-GPF vector using M5 Magic Seamless Assembly and Cloning Mix (Mei5 Bioservices). The resulting constructs, αCA5::GFP, αCA9::GFP, αCA14::GFP and βCA4::GFP, were each transformed into *Agrobacterium* strain EHA105 and then introduced into *Nicotiana benthamiana* by an injection method as previously reported [51]. After 48h of injection, protein expression in tobacco leaves was observed under an Olympus FV-1000 microscope (Olympus,

Janpan). Tobacco leaves were infiltrated with the plasma membrane marker FM4-64 (Molecular Probes, Life Technologies) and Cyto Trace^{TM} (Red Fluorescent Probes-YeSen) at a concentration of 10 mM 10 min prior to imaging. The GFP fluorescence was excited at 488 nm and detected at 500 nm. FM4-64 was excited at 514 nm and detected at 640-760 nm. Cyto Trace^{TM} Red Fluorescent Probe was excited at 577 nm and detected at 602 nm.

Supplementary Method 15. Enzymatic activity assay of extracellular CA in conchocelis

Extracelular CA enzyme activity in conchocelis was measured according to the description in Ye *et al.* with minor modifications [52]. 0.1g (fresh weight) wiped conchocelis were added into 10ml of assay buffer (25mM phosphate buffer (pH8.36) and 10uM $ZnCl_2$, final pH was 8.2). The reaction was initiated by the addition of 2 ml distilled water which was saturated with CO_2 for 1h at 4 °C. Changes of pH values were monitored by a pH meter and the time taken for the pH to fall from 8.2 to 7.5 was recorded. Assay buffer without any conchocelis samples was used as control. The extracellular CA activity was calculated as: Activity unit= 10((tc/ts)-1), where tc and ts were the times for the pH to fall from 8.2 to 7.5 for the control and samples respectively. Three biological replications were done.

Supplementary Table 1. The genome and transcriptome sequencing.

Sequencing Library	Sequencing platform	Library size	Data size (Gb)	Depth
**DNA library**	Illunina	500 bp	10.4	100x
	Illunina	5kb	5.4	50x
	Pacbio	20 kb	7.6	76x
	Bionano		131.8	1,310x
	Hi-C	100-300 bp	12.7	120x
**RNA library**	Pacbio	1-2kb	0.9	
		2-3kb	1.0	
		>3 kb	1.0	

Supplementary Table 2. The step-by-step genome assembly of *P.yezoensis*

	HGAP	FinishSC	SSPACE	PBJelly	Quiver	IrysView	3d-dna-master
**No. of Scaffold**	1,444	933	754	680	680	510	527
**Assembly Size (Mb)**	112.3	107.4	107.6	108.6	108.6	124.3	112.8
**N50 (Kb)**	201.4	256.1	336.9	337.7	337.7	4.32 Mb	34.3Mb
**GC content**	64.75 %	64.88 %	64.79 %	64.79 %	64.80 %	64.88 %	64.75 %
**Max length (Mb)**	1.0	1.2	1.9	1.9	1.9	9.8	43.6
**Ns**	0.00 %	0.00 %	0.13 %	0.03 %	0.02 %	13.21 %	0.43 %

Supplementary Table 3. General information about the *P. yezoensis* genome.

**Genome size (Mb)**	108.4
**No. of scaffolds**	527
**Superscaffold 1(Mb)**	29.4
**Superscaffold 2(Mb)**	34.3
**Superscaffold 3(Mb)**	43.6
**Average GC%**	64.9 %
**Coding potential**	22.0%
**Number of genes**	12,855
**Mean gene length**	2,580
**Mean intron number**	0.56
**Mean exon number/each gene**	1.63
**Mean exon length**	1,136
**Mean intron length**	1,152

Supplementary Table 4. The composition of repeat elements in the *P. yezoensis* genome.

**Repeat class and family**	**Masked (bp)**	**Percentage of genome**
**DNA**	85,255	0.08%
**DNA/CMC-Chapaev**	271,780	0.25%
**DNA/CMC-EnSpm**	369,164	0.34%
**DNA/hAT-Ac**	74,027	0.07%
**DNA/hAT-Tag1**	47,275	0.04%
**DNA/PIF-Harbinger**	1,103,908	1.03%
**DNA/TcMar-Ant1**	525,282	0.49%
**DNA/TcMar-Fot1**	84,934	0.08%
**DNA/TcMar-m44**	112,556	0.10%
**DNA/TcMar-Pogo**	75,779	0.07%
**DNA/TcMar-Tc1**	182,462	0.17%
**DNA/TcMar-Tigger**	163,628	0.15%
**LINE**	104,591	0.10%
**LTR**	177,000	0.16%
**LTR/Copia**	495,015	0.46%
**LTR/DIRS**	450,884	0.42%
**LTR/Gypsy**	19,094,385	17.75%
**LTR/Gypsy-Cigr**	13,305,910	12.37%
**RC/Helitron**	287,601	0.27%
**Simple_repeat**	3,974,839	3.69%
**Unknown**	10,599,684	9.85%
**TOTAL**	51,585,959	47.95%

Supplementary Table 5. The primer sequences used in cloning CA genes.

αCA14 (py06603.t1) F	5' CGACTCTAGAAAGCTTATGGCGAGCGCCAGCGCCTC 3'
αCA14 (py06603.t1) R	5' CGGGCCCCTGCAGAAGCTTCTGCGACGCGTCCGCTGCTGCG 3'
βCA4 (py00618.t1) F	5' CGACTCTAGAAAGCTTATGGCTGCCGTCGCGTCC 3'
βCA4 (py00618.t1) R	5' CGGGCCCCTGCAGAAGCTTGCATTCCTCCACAACCCCCG 3'
αCA5 (py01276.t1) F	5' CGACTCTAGAAAGCTTATGGGCATCCATCGTACGATTGC 3'
αCA5 (py01276.t1) R	5' CGGGCCCCTGCAGAAGCTTGTGCAGGAGACCCGCCTTG 3'
αCA9 (py03023.t1) F	5' CGACTCTAGAAAGCTTATGGCCATCCTTGCCCGTC 3'
αCA9 (py03023.t1) R	5' CGGGCCCCTGCAGAAGCTTCAAGCTCACCTCAAGCCACTCG 3'

Supplementary Table 6. The relative abundance of amino acids with less carbon atoms as encoded in Bangiophyceae genomes compared to Florideophyceae.

No. of carbon atoms	AA
2C	**Gly**
3C	**Ala** **Ser** **Cys**
4C	**Thr** **Asn** **Asp**
5C	**Val** **Gln** **Glu** **Met** **Pro**
6C	**Arg** **Lys** **Ile** **His** **Leu**
9C	**Phe** **Tyr**
11C	**Trp**

The Green and red colors indicated the higher and lower content in Bangiophyceae genomes than that in Florideophyceae genomes. The font size of amino acids represents its average content in Bangiophyceae genomes.

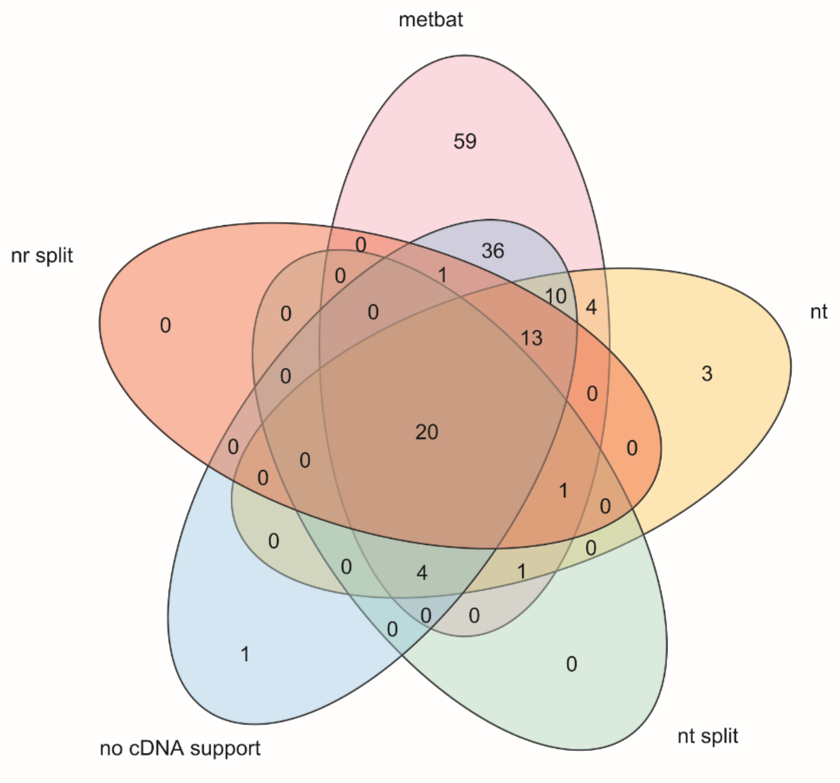

Supplementary Fig. 1. Venn diagram showing the number of contigs that were detected to be potential bacterial sequences by the five methods. Names of each method was listed beside of the diagram component.

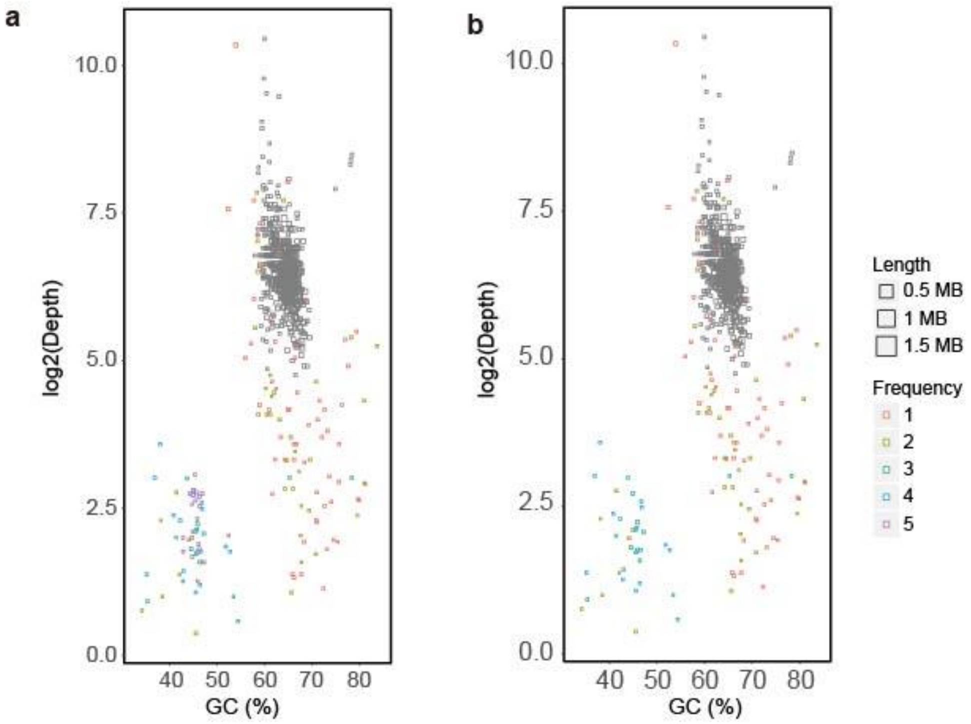

Supplementary Fig. 2. The distribution of GC content in primarily assembled scaffolds before (a) and after (b) filtering out possible bacterial contamination reads. Each scaffold was indicated by a square. Size of the square indicated the length of corresponding scaffold. The square color indicated frequency of being detected as bacterial sequences by the five-step de-contamination pipeline. X-axis stands for the GC contents of scaffolds and Y-axis stands for the sequencing depth.

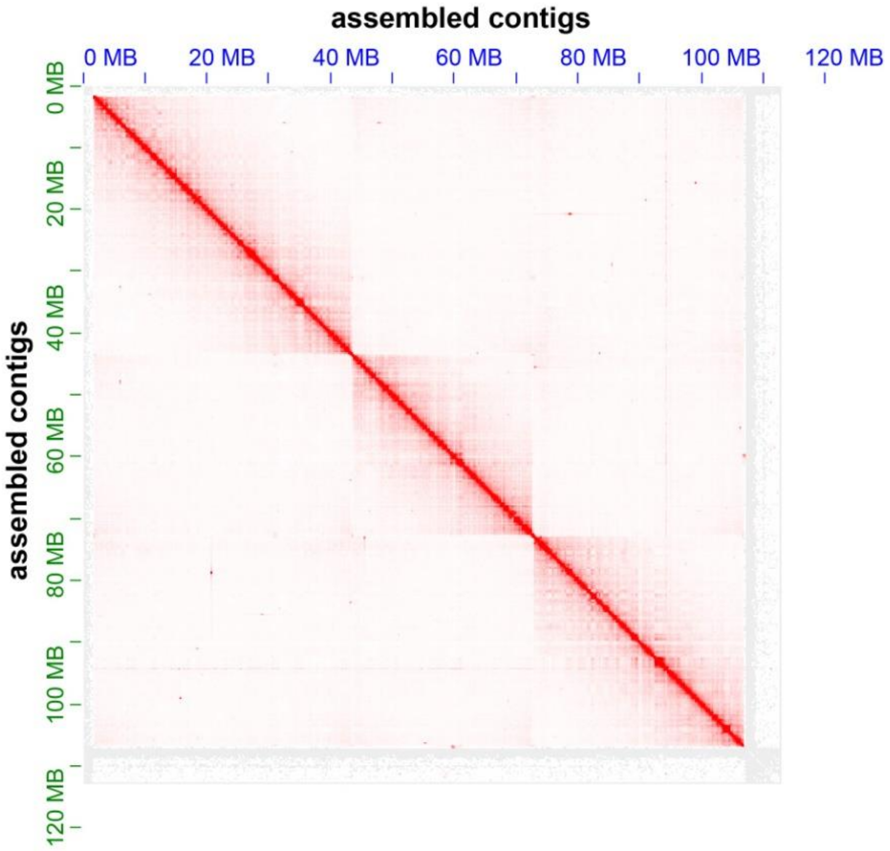

Supplementary Fig. 3. Interactive distribution of Hi-C links among assembled contigs. The two ends of each link were mapped to assembled contigs, and red dots represented the intersection of their corresponding loci in contigs.

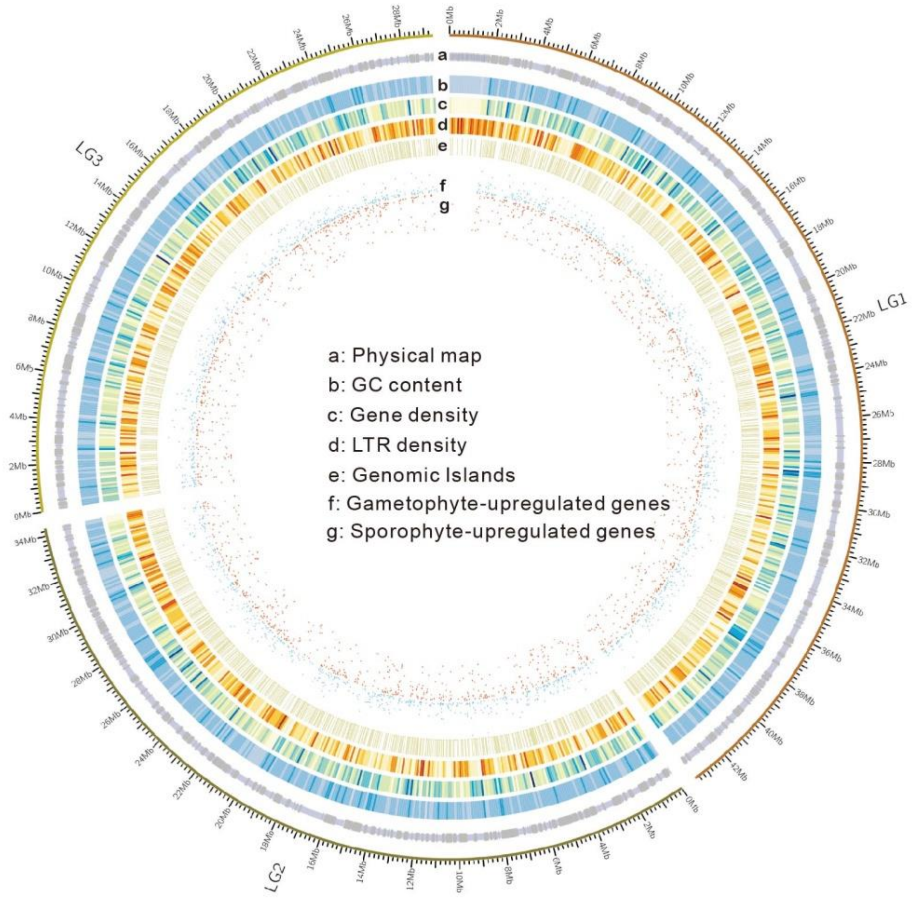

Supplementary Fig. 4. Structural features of the *P. yezoensis* genome. The three chromosomes were presented as LG1, LG2 and LG3. **(a)** Physical map generated from Hi-C data; **(b)** The genome-wide distribution of GC content calculated by 1000-bp windows; **(c)** Gene density; **(d)** The distribution of LTR; **(e)** Genomic islands; **(f)** Gam-specifically transcribed genes; **(g)** Spo-specifically transcribed genes.

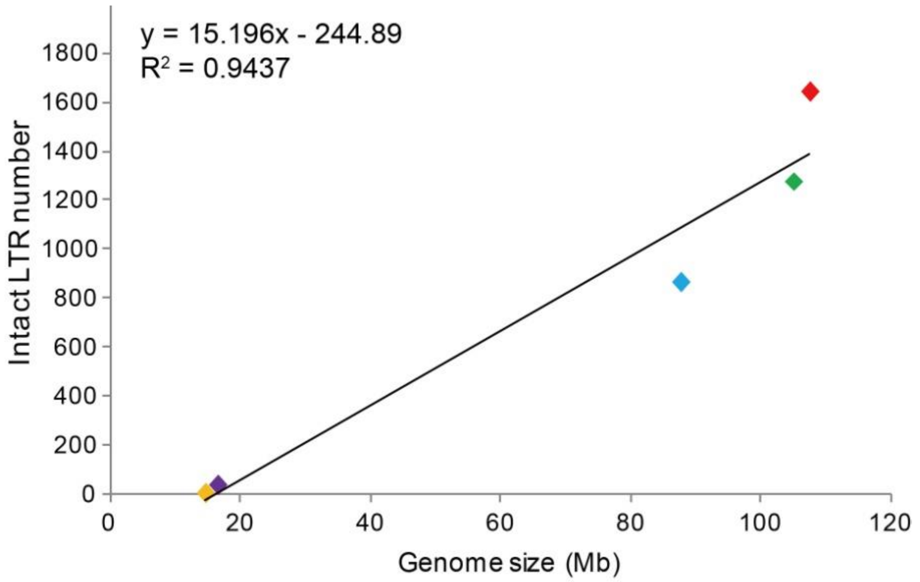

Supplementary Fig. 5. The correlation relationship of intact LTR numbers and genome sizes in red algae. X-axis stands for genome sizes of red algal genomes as indicated by squares with different colors (red: *P. yezoensis*; green: *Chondrus crispus*; blue: *Porphyra umbilicalis*; purple: *Cyanidioschyzon merolae*; orange: *Galderia sulphuraria*). Y-axis stands for the number of intact LTR elements predicted in individual genome by LTRharvest. The linear regression equation for the relationship was placed on the upper left corner.

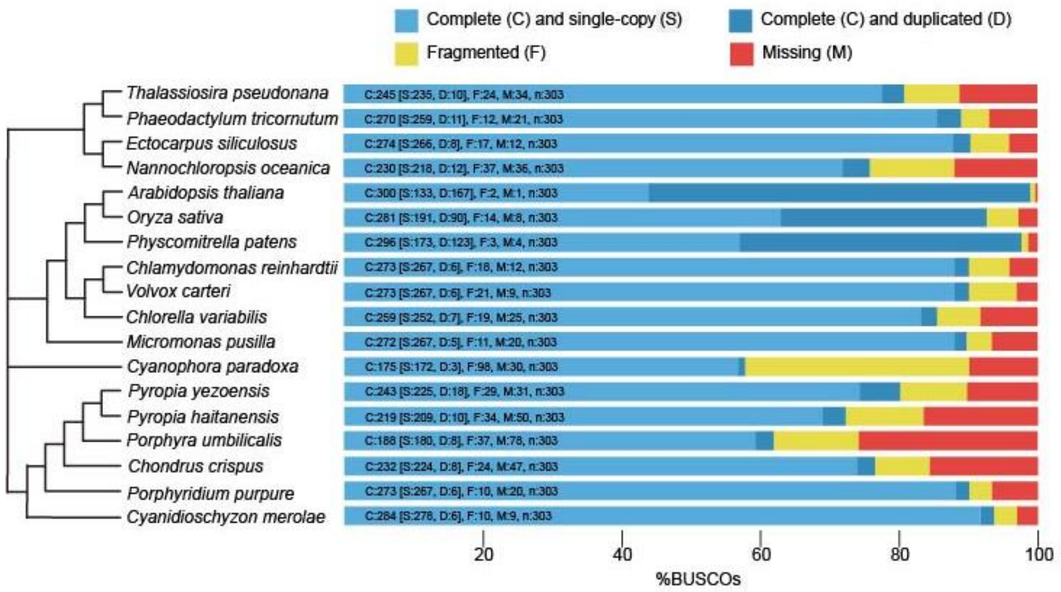

Supplementary Fig. 6. BUSCO analysis of *P.yezoensis* and other published algal genomes to assess the completeness of genome. Protein sequences of gene models in *P.yezoensis*, 15 published algal genomes and three plant genomes were subjected to the BUSCO analysis, using the single copy orthologs of Eukaryota in BUSCO database was used as reference. BUSCO reference orthologs were grouped into four categories: complete and single-copy (blue), complete and duplicate (dark blue), fragmented (yellow) and missing (red) according to BUSCO pipeline. X-axis stands for the percentage of BUSCO orthologs in each category.

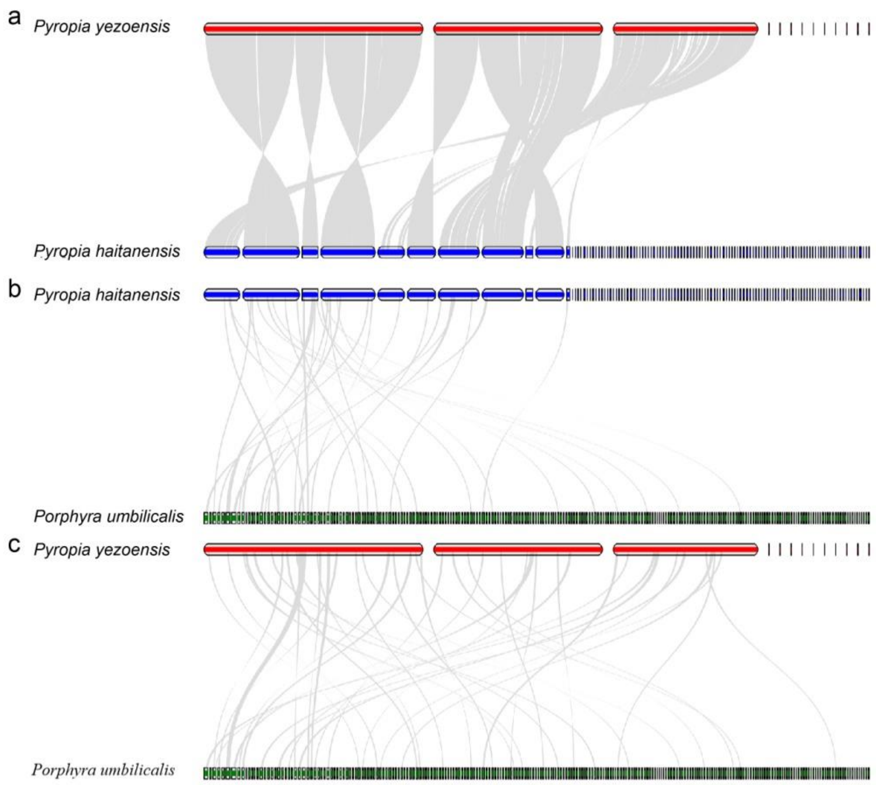

Supplementary Fig. 7. Syntenic regions in Bangiales genomes. The wide horizontal lines in red, blue, and green represent the assembled scaffolds in *P. yezoensis*, *P. haitanensis* and *Po. umbilicalis.* Gray areas represent collinear blocks identified by MCScanX between any two genomes. (**a**) Collinear blocks between *P. yezoensis* and *P. haitanensis*. (**b**) Collinear blocks between *P. haitanensis* and *Po. umbilicalis.* (**c**) Collinear blocks between *P. yezoensis* and *Po. umbilicalis*.

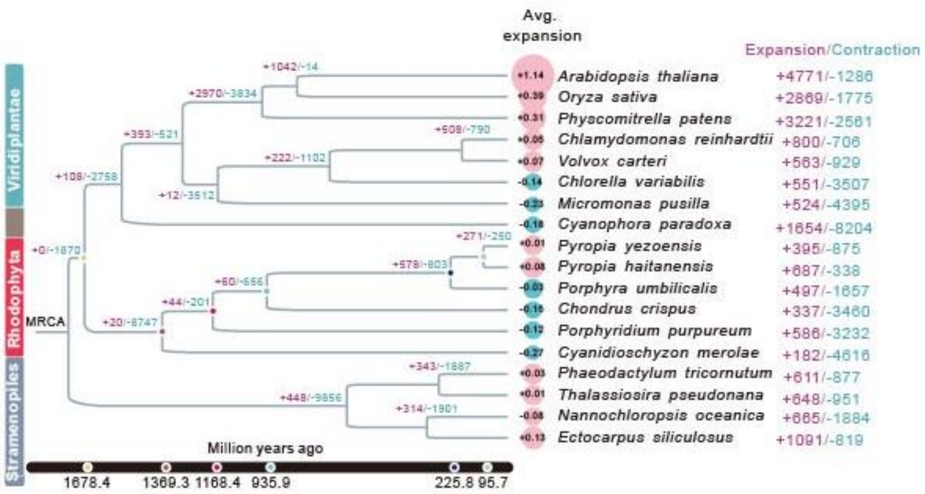

Supplementary Fig. 8. Numbers of contracted or expanded paralogous groups in each evolutionary node. The phylogeny was constructed based on single-copy orthologous genesets identified from *P. yezoensis* and the other published algal and plant genomes. The divergence times are dated and displayed below the phylogenetic tree. The number of expanded (plus sign in pink) and contracted (minus sign in blue) gene families are calculated by CAFE and shown along branches and nodes. MRCA stands for most recent common ancestor. The average gene family expansions along each branch are displayed as pies (pink: expansion; blue: contraction).

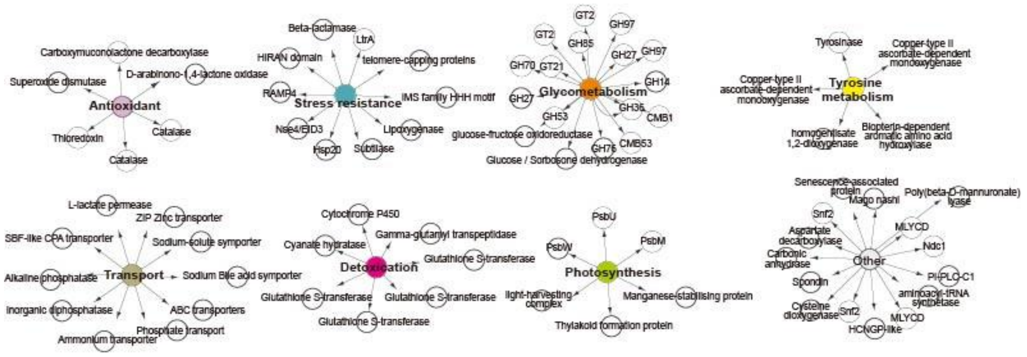

Supplementary Fig. 9. Biological functions encoded by the expanded gene families in *Bangiales* genomes, as annotated by Pfam. Eight categories of biological functions are shown (pink: antioxidant; blue: stress resistence; orange: glycometabolism; yellow: tyrosine metabolism; grey: transport; dark pink: detoxication; green: photosynthesis; white: other functions). The Pfam annotated function of expanded gene families are radially listed in corresponding categories.

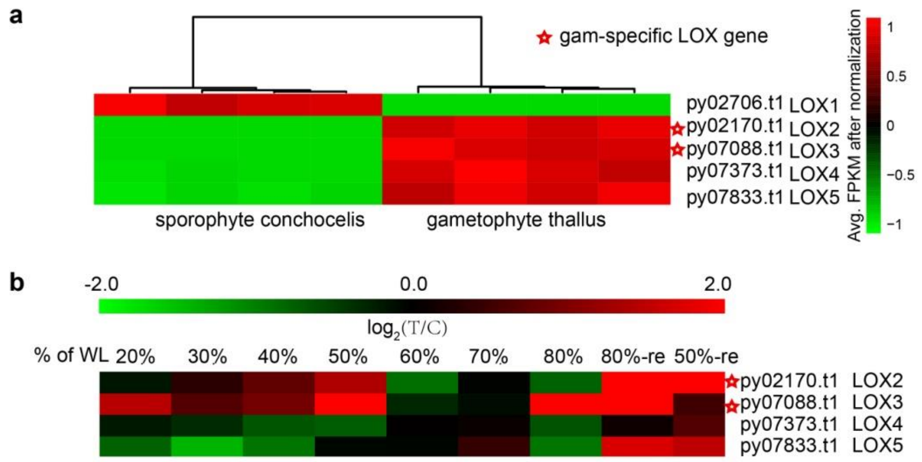

Supplementary Fig. 10. Transcription of tyrosinase genes in two life phases and during osmotic stress. (a) Transcriptional level of LOX paralogs in gametophyte and sporophyte. FPKM values of LOX genes (n=4) in gametophyte and sporophyte were used to draw heat map after a z-score normalization. Gam-transcribed genes were marked by red stars. Sample cluster was done by HCL clustering in Mev 4. Gene IDs were listed at the right side of the heat map. **(b)** Heat map showing the the transcriptional fold changes of LOX paralogs in response to osmotic stresses. Fold change was calculated as stated in Fig. 2. Percentage above each column strands for degree of water loss (WL: water loss). 80%-re and 50%-re are rehydrated samples after 80% and 50% of water loss respectively.

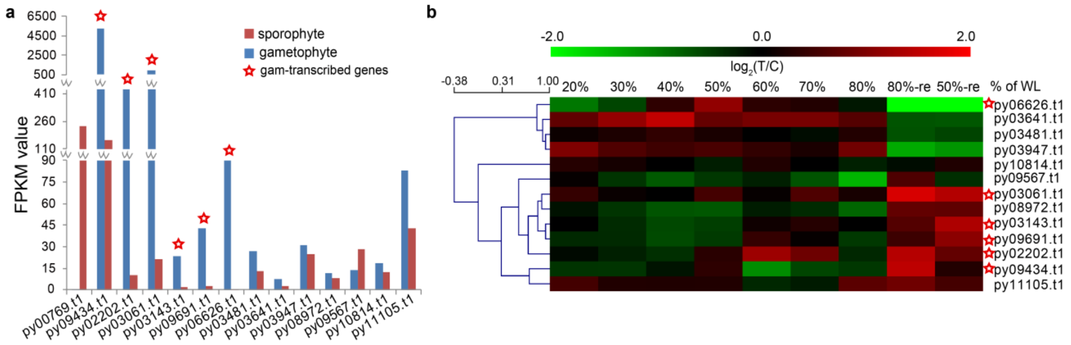

Supplementary Fig. 11. Transcription of tyrosinase genes in two life phases and during osmotic stress. (a) Transcriptional level of tyrosinase paralogs in life cycle. X-axis stands for gene IDs of tyrosinase. Y-axis stands for the average FPKM values in each life phase with blue column indicating gametophyte and red indicating sporophyte. Gam-transcribed genes were marked by red stars. **(b)** Heat map showing the fold changes of tryrosinase paralogs in response to osmotic stresses. Fold change was calculated as stated in Fig. 2. Gene clustering was done by HCL algorithm in Mev 4. Percentage above each column strands for degree of water loss (WL: water loss). 80%-re and 50%-re are rehydrated samples after 80% and 50% of water loss respectively.

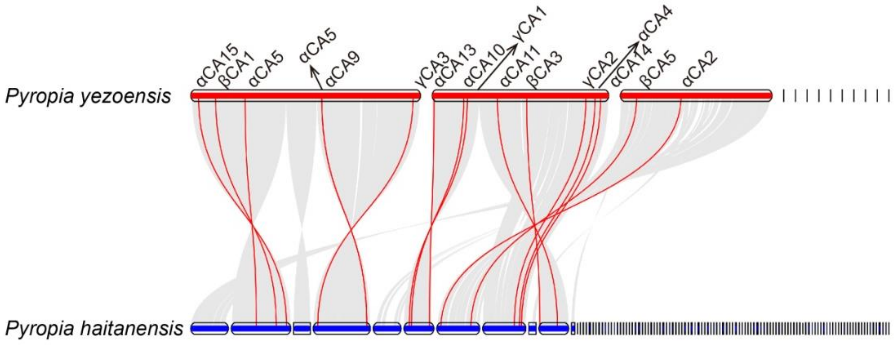

Supplementary Fig. 12. Synteny analysis of CA genes between *P.yezoensis* and *P. haitanensis*. Gray lines in the background indicate the collinear blocks within the two species. Red lines highlight the syntenic CA gene pairs inside of corresponding collinear blocks. αCA5 and αCA9 localize closely to each other, thus lines of them are overlapped.

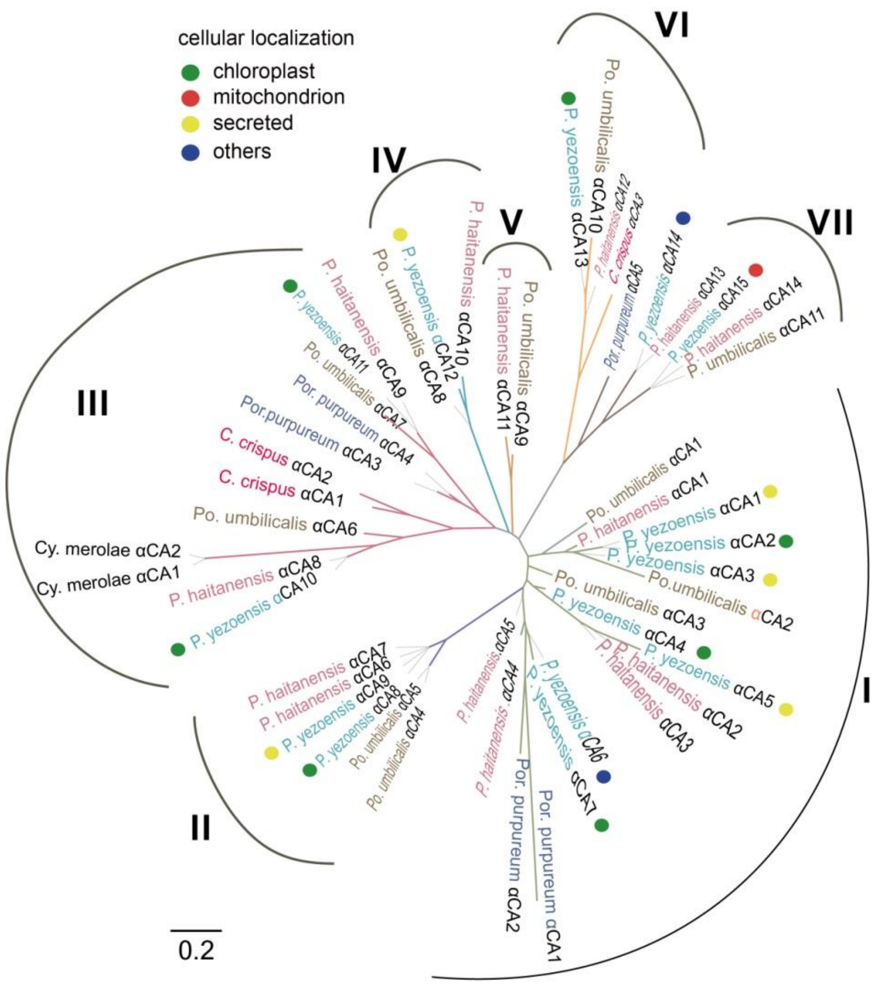

Supplementary Fig. 13. Protein sequence similarity network analysis of red algal αCAs generated seven sub-groups. Sub-groups I-VIII were labeled separately. The five red algal species were color labeled. Periphery color dots indicated sub-cellular localization as predicted via PredAlgo. Green: chloroplast, red: mitochondria; yellow: secreted proteins; blue: other compartments.

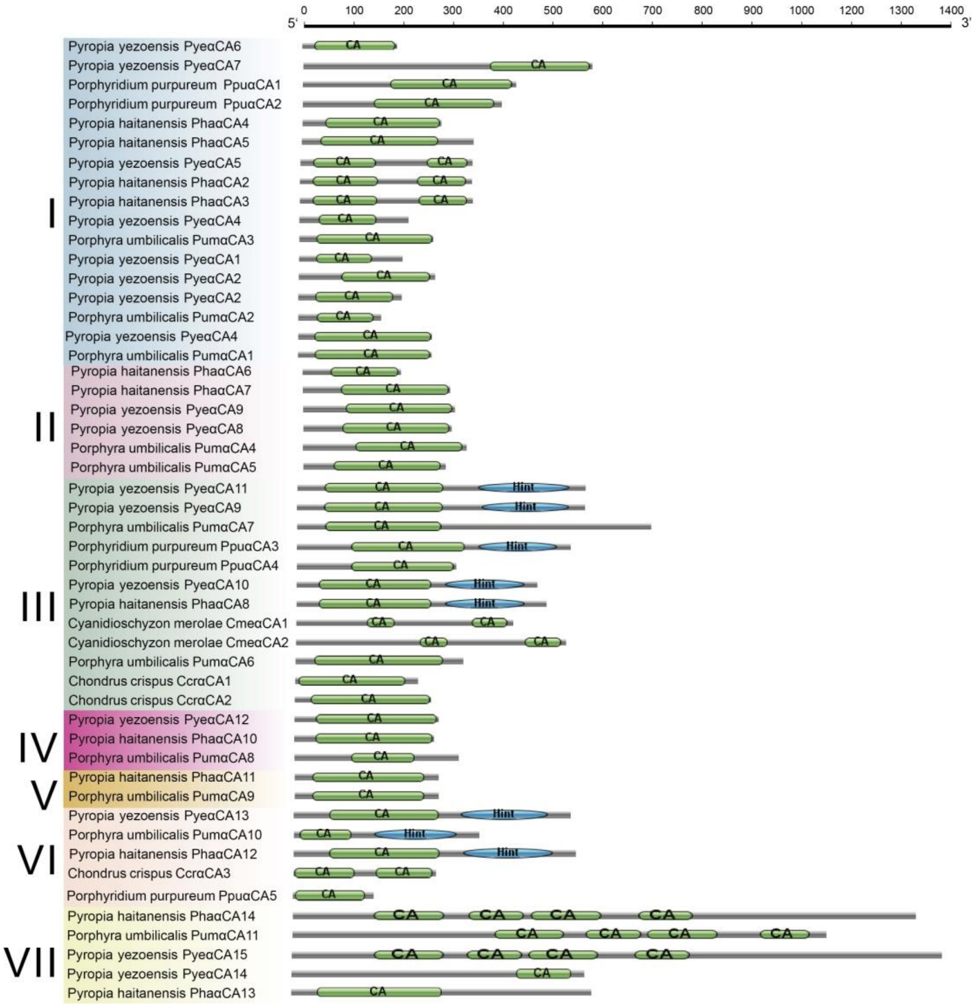

Supplementary Fig. 14. The distribution of conserved domains in αCA isoforms in red algal genomes. The seven sub-groups of αCA isoforms were represented by different colors. Conserved domains were distributed along the length of each CA gene indicated by the scale bar at the top of this figure. Green bar: CA domain; Blue bar: Hint domain.

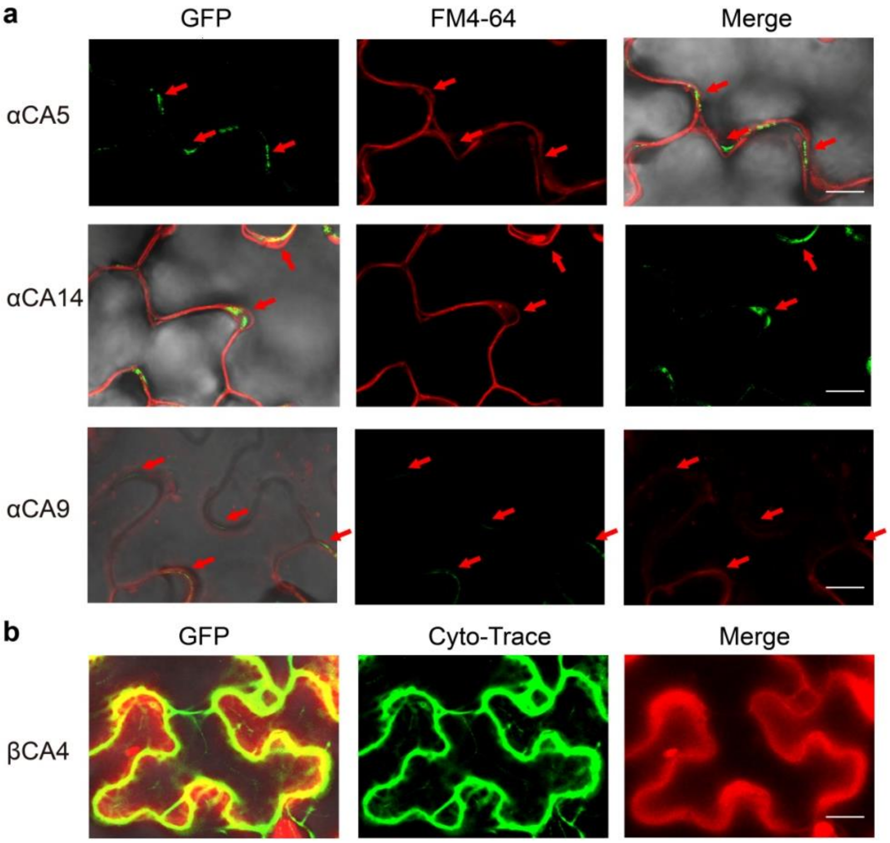

Supplementary Fig. 15. Determining the sub-cellular localization of Pyropia CA isoforms in tobacco. Localizations of the CA:GFP fusion protein were probed by fluorescence microscopy. (a) Extracellular localization of GFP fusion protein for αCA5, αCA9 and αCA14 in tobacco. Green and red signals are CA:GFP and FM4-64 (plasma membrane-specific stain) fluorescence respectively. Merged images of the two signals are show. Red arrows show the extracellular localization of CA: GFP fusion proteins, between the two cell membranes. (b) The cytosolic localization of βCA4:GFP fusion in tobacco. Green and red signals are CA:GFP and Cyto-Trace (cytosol-specific stain) fluorescence respectively. Overlapped signals as indicated by yellow in the merged image suggest βCA4 is cytosolic. Scale bar = 10 μm.

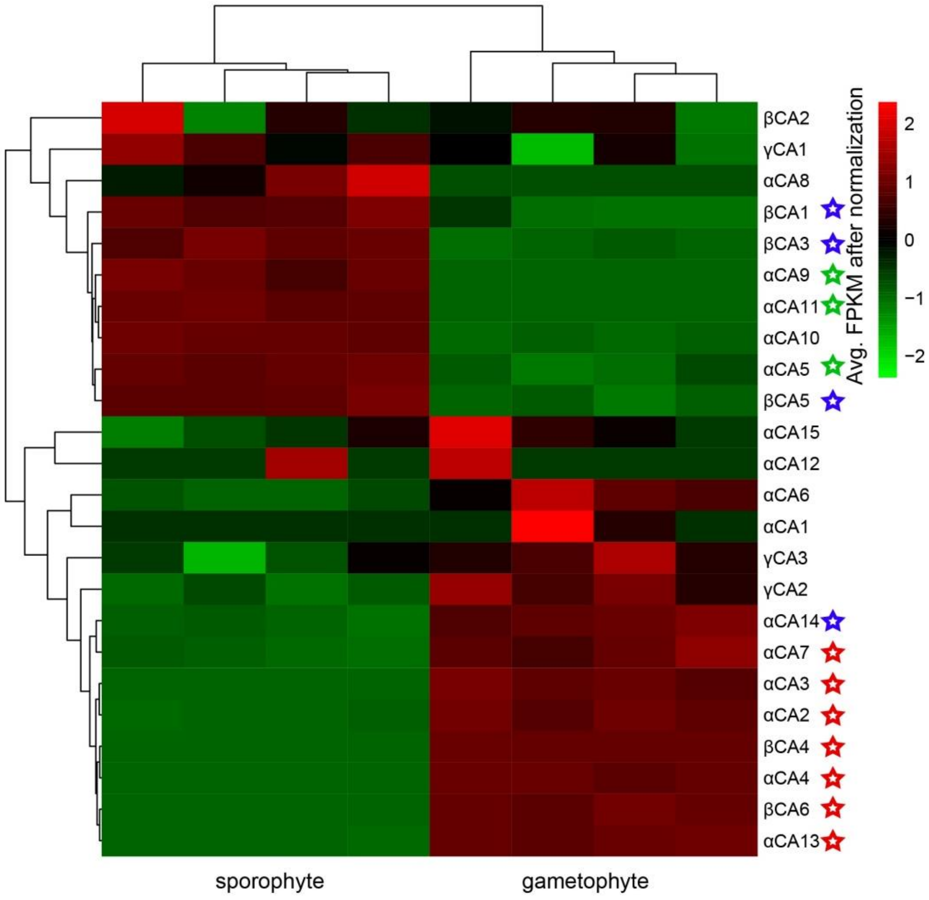

Supplementary Fig. 16. The transcriptional patterns of CA genes in two life phases in *P.yezonensis*. FPKM values of CA genes (n=4) in gametophyte and sporophyte were used to draw heat map after a z-score normalization. CA genes exhibiting specific transcription in gametophyte stage (defined as "gam-specifically-transcribed" genes in main text) are indicated by red stars and spo-specifically-transcribed CAs are indicated by green stars. Those with 2-fold higher of transcriptional level in one stage than the other stage were indicated as blue stars.

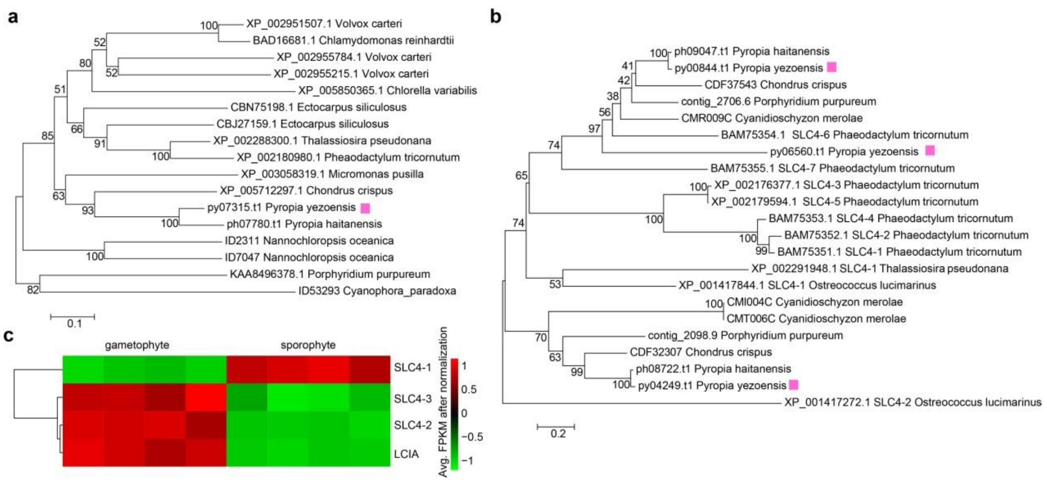

Supplementary Fig. 17. Bicarbonate transporter genes identified in *P.yezoensis* genome. (**a**) Phylogeny of *Pyropia* LCIA genes constructed using Maximal likelihood algorithm in MEGA5 with bootstrap test (100 replicates)**.** Pyropia LCIA gene was indicated by pink square. Accession numbers of LCIA homologous protein sequences in other species are placed beside each evolutionary branch. (**b**) Phylogenetic tree of the three SLC4 genes (indicated by pink square), constructed by the same method as in (**a**). (**c**) The transcriptional patterns of LCIA and SLC4 genes in two life phases in *P.yezonensis*. FPKM values of CA genes (n=4) in gametophyte and sporophyte were used to draw heat map after a z-score normalization.

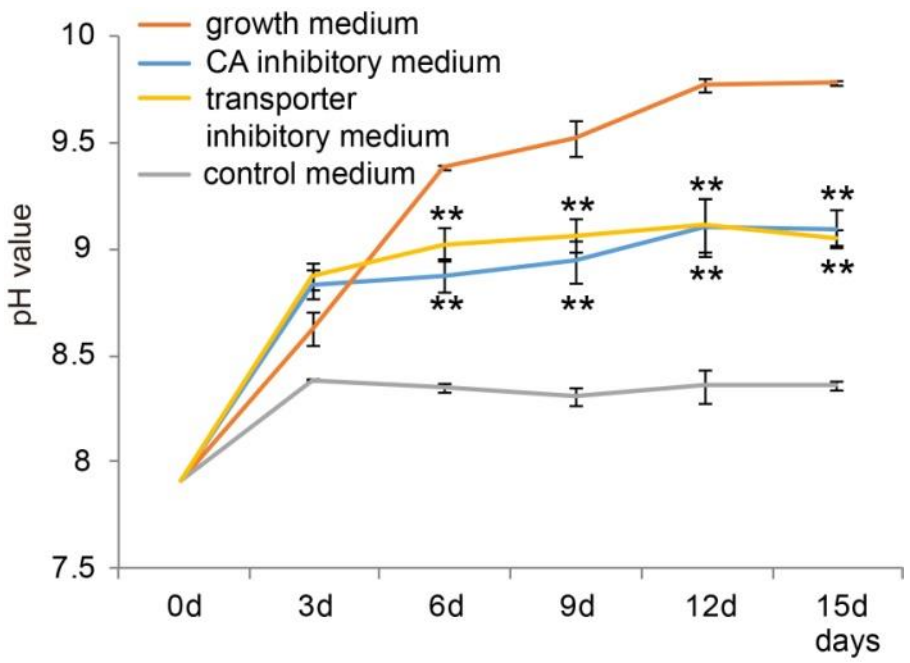

Supplementary Fig. 18. PH values in the vicinity of conchocelis-dwelling shells w/o CA or P-type ATPase inhibited. X-axis stands for the duration of time (days) after shells were placed into the medium. Y-axis stands for the pH values. The 0 time value was 7.9. Control medium (with blank shell), growth medium (with conchocelis-dwelling shells), CA inhibitory medium (acetazolamide was added into growth medium) and transport inhibitory medium (vanadate was added into growth medium) are plotted in grey, red, blue and yellow. The error bars show the standard deviations (n = 3). P values were calculated as described in Supplementary Table7. The two asterisks indicating $p<0.01$ for CA inhibitory medium were placed below the blue plot, while the ones for transporter inhibitory medium were placed above the yellow plot. Source data are provided as a Source Data file.

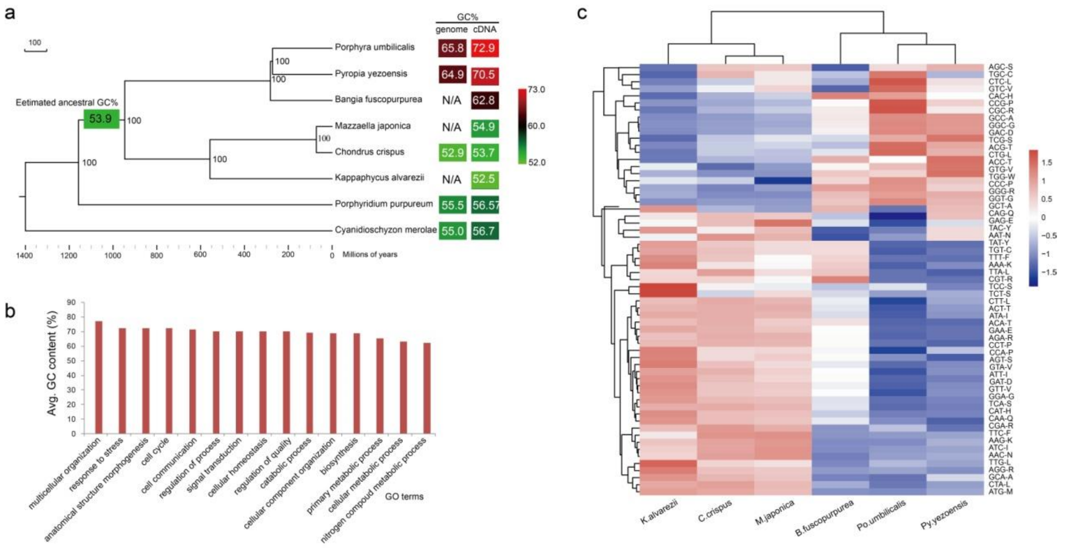

Supplementary Fig. 19. Nucleotide usage in red algal genomes. (a) The variation in GC content in red algal genomes. A maximum likelihood (consensus) tree were built whole-genome based phylogeny of red algae were built. The time was deduced as described in Methods. Average GC content of each genome were placed at the right side of species name; (**b**) Average GC content of functional groups in *P. yezoensis* genome. GC contents of genes related to the corresponding Gene Ontology Slim terms in biological process were calculated. The top 15 functional groups (including the most number of genes) were shown. (**c**) Enrichment of GC-rich codons. Frequency of 61 codons from Bangiophyceae and Florideophyceae species was plotted in heat map and clustered by Euclidean distance and complete linkage clustering module. The datasets was normalized by row.

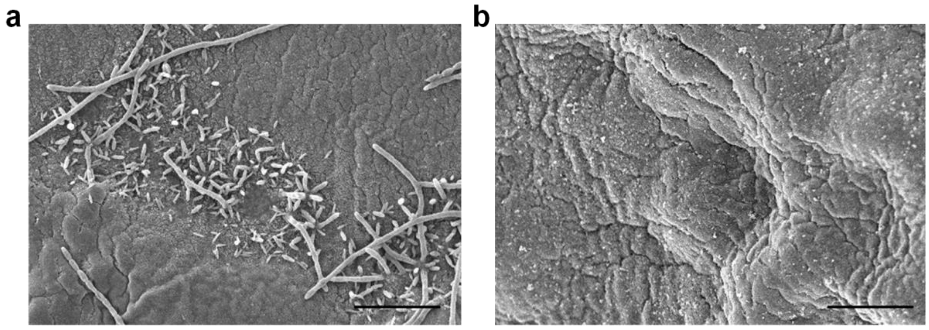

Supplementary Fig. 20. *P. yezoensis* thallus before (a) and after (b) removal of bacterial contamination. Thallus were observed under an electric microscope. The magnification was 2500x. Scale bar = 10 μm.

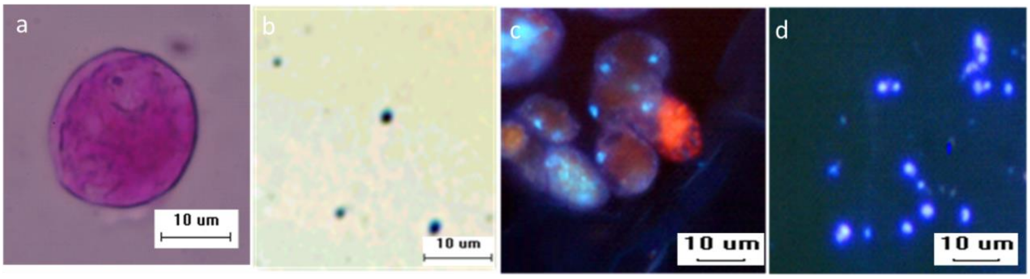

Supplementary Fig. 21. The protoplasts and nuclei in *P. yezoensis* with Carbol fuchsin and DAPI. (**a**) Protoplast chromosome staining with Carbol fuchsin in the protoplasts. (**b**) Isolated nuclear staining with Carbol fuchsin. (**c**) Protoplasts chromosome staining with DAPI. (**d**) Isolated nuclear staining with DAPI.

Supplementary References

1. Lee J*, et al.* Analysis of the draft genome of the red seaweed *Gracilariopsis chorda* provides insights into genome size evolution in Rhodophyta. *Mol Biol Evol* **35**, 1869-1886 (2018).

2. Wang Y*, et al.* MCScanX: a toolkit for detection and evolutionary analysis of gene synteny and collinearity. *Nucleic Acids Research* **40**, e49-e49 (2012).

3. Cao M*, et al.* A chromosome-level genome assembly of *Pyropia haitanensis* (Bangiales, Rhodophyta). *Molecular Ecology Resources* **20**, 216-227 (2020).

4. Brawley SH*, et al.* Insights into the red algae and eukaryotic evolution from the genome of *Porphyra umbilicalis* (Bangiophyceae, Rhodophyta). **114**, E6361 (2017).

5. Tran LT, Taylor JS, Constabel CP. The polyphenol oxidase gene family in land plants: Lineage-specific duplication and expansion. *BMC Genomics* **13**, (2012).

6. Stepien CC. Impacts of geography, taxonomy and functional group on inorganic carbon use patterns in marine macrophytes. *J Ecol* **103**, 1372-1383 (2015).

7. Raven JA, Ball LA, Beardall J, Giordano M, Maberly SC. Algae lacking carbon-concentrating mechanisms. *Can J Bot* **83**, 879-890 (2005).

8. Yamano T, Sato E, Iguchi H, Fukuda Y, Fukuzawa H. Characterization of cooperative bicarbonate uptake into chloroplast stroma in the green alga *Chlamydomonas reinhardtii*. *Proc Natl Acad Sci USA* **112**, 7315-7320 (2015).

9. Nakajima K, Tanaka A, Matsuda Y. SLC4 family transporters in a marine diatom directly pump bicarbonate from seawater. *Proc Natl Acad Sci USA* **110**, 1767-1772 (2013).

10. West JA, McBride DL. Long-term and diurnal carpospore discharge patterns in the Ceramiaceae Rhodomelaceae and Delesseriaceae (Rhodophyta). In: *Sixteenth International Seaweed Symposium* (eds Kain JM, Brown MT, Lahaye M). Springer Netherlands (1999).

11. Zhang H-B, Zhao X, Ding X, Paterson AH, Wing RA. Preparation of megabase-size DNA from plant nuclei. *Plant Journal* **7**, 175-184 (1995).

12. Chaisson MJ, Tesler G. Mapping single molecule sequencing reads using basic local alignment with successive refinement (BLASR): application and theory. *BMC Bioinformatics* **13**, 238 (2012).

13. Bashir A*, et al.* A hybrid approach for the automated finishing of bacterial genomes. *Nature Biotechnology* **30**, 701-707 (2012).

14. Chin C-S*, et al.* Nonhybrid, finished microbial genome assemblies from long-read SMRT sequencing data. *Nature Methods* **10**, 563-569 (2013).

15. Boetzer M, Henkel CV, Jansen HJ, Butler D, Pirovano W. Scaffolding pre-assembled contigs using SSPACE. *Bioinformatics* **27**, 578-579 (2010).

16. English AC*, et al.* Mind the Gap: Upgrading genomes with pacific biosciences RS long-read sequencing technology. *PLoS ONE* **7**, e47768 (2012).

17. Simão FA, Waterhouse RM, Ioannidis P, Kriventseva EV, Zdobnov EM. BUSCO: Assessing genome assembly and annotation completeness with single-copy orthologs. *Bioinformatics* **31**, 3210-3212 (2015).

18. Huson DH*, et al.* MEGAN community edition - interactive exploration and analysis of large-scale microbiome sequencing data. *PLoS Computational Biology* **12**, e1004957 (2016).

19. Kent WJ. BLAT-the BLAST-like alignment tool. *Genome Research* **12**, 656-664 (2002).

20. Love MI, Anders S, Huber W. Moderated estimation of fold change and dispersion for RNA-seq data with DESeq2. *Genome Biology* **15**, 550 (2014).

21. Mao YX, Chen NC, Cao M, Chen R, Guan XW, Wang DM. Functional characterization and evolutionary analysis of glycine-betaine biosynthesis pathway in red seaweed *Pyropia yezoensis*. *Mar Drugs* **17**, (2019).

22. Stanke M, Keller O, Gunduz I, Hayes A, Waack S, Morgenstern B. AUGUSTUS: *ab initio* prediction of alternative transcripts. *Nucleic Acids Research* **34**, W435-W439 (2006).

23. Haas BJ*, et al.* Automated eukaryotic gene structure annotation using EVidenceModeler and the program to assemble spliced alignments. *Genome Biology* **9**, R7 (2008).

24. Deng YY, Li JQ, Wu SF, Zhu YP, He FC. Integrated nr database in protein annotation system and its localization. *Computer Engineering* **32**, 71-72 (2006).

25. Bairoch A, Apweiler R. The SWISS-PROT protein sequence database and its supplement TrEMBL in 2000. *Nucleic Acids Research* **28**, 45-48 (2000).

26. Tatusov RL, Galperin MY, Natale DA, Koonin EV. The COG database: A tool for genome-scale analysis of protein functions and evolution. *Nucleic Acids Research* **28**, 33-36 (2000).

27. Marchler-Bauer A*, et al.* CDD: NCBI's conserved domain database. *Nucleic Acids Research* **43**, D222-D226 (2014).

28. Lombard V, Golaconda Ramulu H, Drula E, Coutinho PM, Henrissat B. The carbohydrate-active enzymes database (CAZy) in 2013. *Nucleic Acids Research* **42**, D490-D495 (2013).

29. Conesa A, Götz S, García-Gómez JM, Terol J, Talón M, Robles M. Blast2GO: A universal tool for annotation, visualization and analysis in functional genomics research. *Bioinformatics* **21**, 3674-3676 (2005).

30. Zdobnov EM, Apweiler R. InterProScan – an integration platform for the signature-recognition methods in InterPro. *Bioinformatics* **17**, 847-848 (2001).

31. Consortium TI*, et al.* InterPro: An integrated documentation resource for protein families, domains and functional sites. *Briefings in Bioinformatics* **3**, 225-235 (2002).

32. Ogata H, Goto S, Sato K, Fujibuchi W, Bono H, Kanehisa M. KEGG: Kyoto Encyclopedia of Genes and Genomes. *Nucleic Acids Research* **27**, 29-34 (1999).

33. Tardif M*, et al.* PredAlgo: A new subcellular localization prediction tool dedicated to green algae. *Mol Biol Evol* **29**, 3625-3639 (2012).

34. Krogh A, Larsson B, von Heijne G, Sonnhammer ELL. Predicting transmembrane protein topology with a hidden Markov model: Application to complete genomes. *J Mol Biol* **305**, 567-580 (2001).

35. Tarailo-Graovac M, Chen N. Using RepeatMasker to identify repetitive elements in genomic sequences. *Current Protocols in Bioinformatics* **25**, 4.10.11-14.10.14 (2009).

36. Lowe TM, Eddy SR. tRNAscan-SE: A program for improved detection of transfer RNA genes in genomic sequence. *Nucleic Acids Research* **25**, 955-964 (1997).

37. Griffiths-Jones S, Moxon S, Marshall M, Khanna A, Eddy SR, Bateman A. Rfam: Annotating non-coding RNAs in complete genomes. *Nucleic Acids Research* **33**, D121-D124 (2005).

38. Emms DM, Kelly S. OrthoFinder: Solving fundamental biases in whole genome comparisons dramatically improves orthogroup inference accuracy. *Genome Biology* **16**, 157 (2015).

39. Huelsenbeck JP, Ronquist F. MRBAYES: Bayesian inference of phylogenetic trees. *Bioinformatics* **17**, 754-755 (2001).

40. Rambaut A, Drummond AJ, Xie D, Baele G, Suchard MA. Posterior summarization in Bayesian phylogenetics using Tracer 1.7. *Systematic Biology* **67**, 901-904 (2018).

41. De Bie T, Cristianini N, Demuth JP, Hahn MW. CAFE: A computational tool for the study of gene family evolution. *Bioinformatics* **22**, 1269-1271 (2006).

42. Altschul SF, Gish W, Miller W, Myers EW, Lipman DJ. Basic local alignment search tool. *J Mol Biol* **215**, 403-410 (1990).

43. Edgar RC. MUSCLE: A multiple sequence alignment method with reduced time and space complexity. *BMC Bioinformatics* **5**, 1-19 (2004).

44. Guindon S, Dufayard JF, Lefort V, Anisimova M, Hordijk W, Gascuel O. New algorithms and methods to estimate maximum-likelihood phylogenies: Assessing the performance of PhyML 3.0. *Systematic Biology* **59**, 307-321 (2010).

45. O'Brien EA*, et al.* TBestDB: A taxonomically broad database of expressed sequence tags (ESTs). *Nucleic Acids Research* **35**, D445-D451 (2007).

46. Keeling PJ*, et al.* The marine microbial eukaryote transcriptome sequencing project (MMETSP): Illuminating the functional diversity of eukaryotic lLife in the oceans through transcriptome sequencing. *PLoS Biol* **12**, (2014).

47. Boguski MS, Lowe TMJ, Tolstoshev CM. Dbest - Database for Expressed Sequence Tags. *Nat Genet* **4**, 332-333 (1993).

48. Katoh K, Asimenos G, Toh H. Multiple alignment of DNA sequences with MAFFT. In: *Bioinformatics for DNA Sequence Analysis* (ed. Posada D). Humana Press (2009).

49. Nguyen L-T, Schmidt HA, von Haeseler A, Minh BQ. IQ-TREE: A fast and effective stochastic algorithm for estimating maximum-likelihood phylogenies. *Mol Biol Evol* **32**, 268-274 (2014).

50. Nowack EC, Price DC, Bhattacharya D, Singer A, Melkonian M, Grossman AR. Gene transfers from diverse bacteria compensate for reductive genome evolution in the chromatophore of Paulinella chromatophora. *P Proc Natl Acad Sci USA* **113**, 12214-12219 (2016).

51. Zhao HM, Tan ZL, Wen XJ, Wang YC. An improved syringe agroinfiltration protocol to enhance transformation efficiency by combinative use of 5-Azacytidine, ascorbate acid and Tween-20. *Plants-Basel* **6**, (2017).

52. Ye C, Gao K, Giordano M. The odd behaviour of carbonic anhydrase in the terrestrial cyanobacterium *Nostoc flagelliforme* during hydration-dehydration cycles. *Environ Microbiol* **10**, 1018-1023 (2008).
