# Supplementary material for: Pyropia yezoensis genome reveals diverse mechanisms of carbon acquisition in the intertidal environment
Source: Nat Commun. 2020 Aug 12;11:4028. doi: 10.1038/s41467-020-17689-1 (PMC7423979; doi:10.1038/s41467-020-17689-1)
Supplement: Supplementary file 3 — Reporting Summary [file 41467_2020_17689_MOESM3_ESM.pdf]

nature research

Corresponding author(s): Yunxiang Mao, Debashish Bhattacharya

Last updated by author(s): Jun 30, 2020

Reporting Summary

Nature Research wishes to improve the reproducibility of the work that we publish. This form provides structure for consistency and transparency in reporting. For further information on Nature Research policies, see Authors & Referees and the Editorial Policy Checklist .

Statistics

For all statistical analyses, confirm that the following items are present in the figure legend, table legend, main text, or Methods section.

n/a	Confirmed	
☐	☒	The exact sample size (*n*) for each experimental group/condition, given as a discrete number and unit of measurement
☐	☒	A statement on whether measurements were taken from distinct samples or whether the same sample was measured repeatedly
☐	☒	The statistical test(s) used AND whether they are one- or two-sided<br>*Only common tests should be described solely by name; describe more complex techniques in the Methods section.*
☒	☐	A description of all covariates tested
☒	☐	A description of any assumptions or corrections, such as tests of normality and adjustment for multiple comparisons
☐	☒	A full description of the statistical parameters including central tendency (e.g. means) or other basic estimates (e.g. regression coefficient) AND variation (e.g. standard deviation) or associated estimates of uncertainty (e.g. confidence intervals)
☐	☒	For null hypothesis testing, the test statistic (e.g. *F*, *t*, *r*) with confidence intervals, effect sizes, degrees of freedom and *P* value noted<br>*Give P values as exact values whenever suitable.*
☐	☒	For Bayesian analysis, information on the choice of priors and Markov chain Monte Carlo settings
☒	☐	For hierarchical and complex designs, identification of the appropriate level for tests and full reporting of outcomes
☒	☐	Estimates of effect sizes (e.g. Cohen's *d*, Pearson's *r*), indicating how they were calculated

Our web collection on statistics for biologists contains articles on many of the points above.

Software and code

Policy information about availability of computer code

Data collection	No software was used for data collection.
Data analysis	SMRT Analysis v2.3.0, RS_HGAP_Assembly.3, SSPACE, PBJelly, IrysView, 3D-DNA software, Repeatmodeler, Tandem Repeats Finder, AUGUSTUS, PASA, EVM, PredAlgo, TMHMM (v2.0) software, etc., were referenced in the manuscript.

For manuscripts utilizing custom algorithms or software that are central to the research but not yet described in published literature, software must be made available to editors/reviewers. We strongly encourage code deposition in a community repository (e.g. GitHub). See the Nature Research guidelines for submitting code & software for further information.

Data

Policy information about availability of data

All manuscripts must include a data availability statement. This statement should provide the following information, where applicable:

- Accession codes, unique identifiers, or web links for publicly available datasets
- A list of figures that have associated raw data
- A description of any restrictions on data availability

Whole genome sequencing data from P. yezoensis were deposited in NCBI under the BioProject PRJNA589917. Genomic sequencing raw data were deposited under SRR10480798 (Illumina platform), SRR10484745 (Hi-C platform) and SRR10489006- SRR10489010 (PacBio platform). The final assembly is available at DDBJ/ ENA/ GenBank under the accession number WMLA00000000. The version described in this paper is version WMLA01000000. The transcriptome sequencing data were deposited under SRR10502194-SRR10502223 (osmotic stresses), SRR10527930- SRR10527937 (two life cycle stages) and SRR10502264- SRR10502266 (full-length cDNA sequencing using the PacBio platform). These accession numbers are released upon this submission. The genome assembly as well as predicted gene models were available at http://mgb.ouc.edu.cn/dongmei/. NR, InterPro, GO, KOG, KEGG, etc., were referenced in the manuscript.

Field-specific reporting

Please select the one below that is the best fit for your research. If you are not sure, read the appropriate sections before making your selection.

- [x] Life sciences
- [ ] Behavioural & social sciences
- [ ] Ecological, evolutionary & environmental sciences

For a reference copy of the document with all sections, see nature.com/documents/nr-reporting-summary-flat.pdf

Life sciences study design

All studies must disclose on these points even when the disclosure is negative.

Sample size	No sample size calculation was performed. The sample sizes were determined according to the required amounts of DNA and RNA to construct the sequencing library. For each genome sequencing library, we collected 4-5g Pyropia thalli (400-500 individuals) and ground together to isolate genomic DNA. For transcriptome sequencing, 5-6 thalli under osmotic stresses were collected together as an individual biological replicate. For conchocelis transcriptome sequencing, 0.2g conchocelis were ground together to isolate total RNA.
Data exclusions	No data were excluded from the analyses.
Replication	We applied four biological replicates for each sample in transcriptome sequencing. Three biological replicates were done in Ca2+ releasing assay in Pyropia conchocelis. Microscope observation of Pyropia life cycle in Fig.1 and conchocelis in Fig. 4a-b, were repeated independently on multiple Pyropia samples (n>10) showing similar morphology.
Randomization	Pyropia samples were randomized during sample processing but otherwise randomization was not applicable in this study.
Blinding	Blinding test were not performed as it was not relevant to this study.

Reporting for specific materials, systems and methods

We require information from authors about some types of materials, experimental systems and methods used in many studies. Here, indicate whether each material, system or method listed is relevant to your study. If you are not sure if a list item applies to your research, read the appropriate section before selecting a response.

Materials & experimental systems

n/a	Involved in the study	
x		Antibodies
x		Eukaryotic cell lines
x		Palaeontology
x		Animals and other organisms
x		Human research participants
x		Clinical data

Methods

n/a	Involved in the study	
x		ChIP-seq
x		Flow cytometry
x		MRI-based neuroimaging
