# Supplementary material for: Pyropia yezoensis genome reveals diverse mechanisms of carbon acquisition in the intertidal environment
Source: Nat Commun. 2020 Aug 12;11:4028. doi: 10.1038/s41467-020-17689-1 (PMC7423979; doi:10.1038/s41467-020-17689-1)
Supplement: Supplementary file 2 — Peer Review [file 41467_2020_17689_MOESM2_ESM.pdf]

Reviewers' comments:

Reviewer #1 (Remarks to the Author):

The authors report the sequencing of the genome of the red alga Pyropia yezoensis. The quality of the genome assembly at chromosomal level seems to be better than published genomes of red alga. The inorganic carbon availability of the Pyropia is analyzed by the high-quality genome. They tested whether the conchocelis Pyropia utilize calcium carbonate of shells as a carbon source by the aid of extracellular carbonic anhydrase (CA). The important data by the isotope supports their hypothesis but the function of CA is not analyzed. Expanded gene families and HGT candidates are analyzed with differential gene expressions and seem to be related to the adaptation to dehydration stress. Those genes that were supported by reliable expressions are interesting and likely include novel findings. Are they clustered into the specific region of chromosomes such as subtelomeres or high GC content? The paper is well written but I have several concerns.

Detailed remarks:
Line 42: The inorganic carbon of shells is extracellular. What is extracellular release?
Line 43: CA is carbonic anhydrase?
Line 43: I think that "This result" should be changed to "this hypothesis".
Line 49: South Korean?
Line 71: "nori" is used for "Pyropia". In other sentences, "nori" means Pyropia yezoensis.
Line 78: ot?
Line 95: Is the method for the removal of bacterial sequences indicated?
Line 98: Do the long scaffolds have telomeric sequences?
Line 182: Is the protein sequence with the four CA domains supported by transcriptome data?
Line 200: Are the two CAs highly expressed in the chonchocelis?
Line 237: How many gene families in the 51 genes?
Line 239: What kinds of bacteria are likely to be related the HGT? Does it include cyanobacteria?
Line 259: Are the eight genes marked in Fig. 5?
Line 265: Are found CpG islands in the genome of the high GC content?
Line 351: How were the samples de-contaminated?
Line 400: Are the pH values shown?
Fig. 4b: What does the red arrow indicate?

Reviewer #2 (Remarks to the Author):

The authors have sequenced, assembled and analyzed the genome of the red algae P. yezoensis, a member of Bangiophyceae. This species is an important cultivated marine crop, also named Nori, and is consumed worldwide.

This alga presents several interesting evolutionary, physiological and ecological characteristics related to its life cycle that involves different types of morphology. These characteristics include tolerance to emersion stress. For example, the authors have investigated the acquisition of inorganic carbon from conchocelis that live in shells, an open and important question. They obtained suggestive evidence that the origin of carbon used in photosynthesis would be the shell through several reactions, the result of several adaptations including HGTs from bacteria, gene expansion of certain families, and a reduction of carbon in DNA and proteins.
As mentioned by the authors, two related genomes, P. umbilicalis and P. haitanensis have been sequenced and published.

One of the main issues with this manuscript is that it is difficult to follow and understand certain parts of the work that has been done and the results, especially because of the figures. Since the

manuscript contains results that are potentially important in genomics, evolutionary science and molecular ecology, I recommend that the authors get help in professional scientific illustration and writing especially for the figures and supplementary text.

This manuscript presents a longterm effort, including many bioinformatic and physiological analyses. This large amount of work deserves a better presentation with clearer organization. Despite a reasonable number of pages, important data acquisition and analysis must be better explained, many points are too superficial and many important technical details are missing. For example, in Supplementary Information, most Supplementary Figures have very incomplete legends preventing a clear understanding of the analysis. In particular, in Fig S15 which seems to be important, there is no description of the experiment that has been done, no description of the three curves.
Also, there is no reference to the supplementary text from the main text. Some chapters of the supplementary text look like entire chapters that were extracted from the main text which therefore looks unbalanced in some places. A clear connection between main and supplementary text is really missing.
Apparently, the authors have generated a high quality assembly at the chromosome level. This is a very important effort for the community, and I hope this assembly will be integrated into international bioinformatics platforms. It is a bit surprising that the authors did not profit from this important achievement for deeper evolutionary and comparative analysis of genome structures. Most, or even all, of the analyses presented in this manuscript are based on gene sequences and are not linked to the structure of the genome. Maybe leverage the genome structure could help to better illuminate some questions raised by the authors.
The discussion about high GC as a relic of an ancient adaptation to carbon limitation in the Permo-Carboniferous era is interesting but not convincing. The rate of GC percent rapidly evolves in genomes, especially on third bases of course. For example, in other eukaryotes where recurrent HGT has been demonstrated, the GC% of more ancient HGT genes are closer to the GC% of the host genome compared to more recent HGTs.
Overall, I am inclined to follow the authors in their conclusions but they need to significantly improve figures, methods and text. There is ample matter for a clear and logical presentation for this manuscript. The abstract must be reshaped to better connect results into clear messages.
In summary, this is a potentially interesting contribution. My major criticism is that the presentation (text, figures, logical flow) is of insufficient quality and must be greatly improved before publication.

Detailed points:
• The subtitle "Orthogroup analysis" is too technical.
• Genome Assembly: did authors estimate sequence quality at the nucleotide level as it is not clear if they used Illumina reads to correct errors?
• There is not enough information about gene calling methodology.
• Line 116: "significantly expanded": is there any statistics supporting this affirmation ?
• Line 129: the terms "spo-specific" and "gam-specific" are not appropriate since the corresponding genes are not specific of these life history stages.
• Line 137: ROS-induced-cell is not defined.
• Line 144: The 2 genes that are highly expressed in Sporophyte are not shown in figure 2b (py00127.t1, py01435.T1). This must be clarified.
• Line 180-181. References are needed.
• Line 185: The authors list 6 genes with high or specific expression in the thallus, but Fig S14 that is referred to in this context shows more than 6 genes (ex AlphaC13). Why? This is puzzling.
• Line 193 and Fig 3b: There is no explanation of the cellular compartment indicated in the figure and no methodology explained to predict "N terminal plastid targeting sequence".
• Line 194- 195: As explained previously, the Fig S15 misses all information required to understand how it supports the sentence.
• Lines 205-212: this experiment is really interesting. But in Fig 4c, authors must at least use the same terms they used in the main text. Do not name the curves as "acetazolamide", "growing conchocelis" and "blank shell", this is not relevant. For example use "control" , "inhibitory"….

• Line 229: Fig 4d instead of 5d I suppose.
• The HGT analysis is really interesting. Is it possible to estimate the number of different events of transfer and to date them?
• Statistical tests are needed to support comparisons of frequencies of G/C to A/T.
• The section about HGT requires a conclusion. How is the pattern of high GC related to the biology of the species here? Is it related to the adaptation of Ci acquisition adaptation?
• Fig S4 indicates a physical map from Hi-C. How was it produced and used in the assembly? The authors must provide more technical information. The algorithm to integrate the optical map and Hi-C is not described.
• Line 365: FPKM is not introduced and there is no reference for the cufflink program.
• Line 367: Calibration of Augustus is not explained.
• Fig S1, S2, S5 to S9, S13 to S16: Legends are too short. More explanation and description are needed.
• Line 395: define OG
• Fig 1: It would be interesting to indicate ploidy of each state, where meiosis occurs.
• Fig 2: This figure is central to the main results but poorly explained, the important information is not underlined. Importantly, there is no statistics provided to support the figure. In panel a, authors should mention the origin of the tree, it would be interesting to annotate the nodes in the tree by genomic, biological or physiological information. Also, it would be useful to add in this panel the expression folding in addition to gene number expansion. Fig 2b is meaningless. The unit of the Y axis is not explained, it is not clear that the X axis represents genes. This figure does not show the expression over the life cycle as written in the title and in the legend since only 2 stages are shown: sporophyte conchocelis and gametophyte thallus.
• Fig 3: Stramenopile, not "Stramenophiles" I suppose. In panel b the dendrogram is not explained. In panel a, the number of alpha CA does appears higher in Pyropia and Porphyra than in most branches but not significantly higher than in rice for example.
• Fig 4: Panel c: The 3 curves must be explained in the legend, time is not mentioned (X axis). Panel d needs more explanations. How many replicates were done? Standard deviation is missing.
• Fig 5: This figure shows the importance of HGT genes in the repertoire of gam-specific and spo-specific genes. Functions of these genes are indeed biased, but panel c is not demonstrative. In panel c, HGT genes display a range of types of expression for different stress conditions, but GAM specific genes do not seem to hold any peculiarity. Maybe a comparison of HGT versus non HGT genes would be more demonstrative.
• More methodological information about HGT detection must be provided as it is an important analysis.
• Expansion of CA: did the authors investigate chromosomal locations of these copies in comparison to other species? Is there interesting information allowing an hypothesis about evolution of this function?
• The analysis about SOD expansion is very interesting however figures should be improved and more conclusive. It is curious that the authors do not comment at all in the main text their analysis of lipoxygenase and tyrosinases.

Reviewer #3 (Remarks to the Author):

The manuscript uses a genomic approach to investigate mechanisms of carbon acquisition of an economically and ecologically important marine macroalga, with emphasis on the intriguing problem of how and why the diploid phase in the life cycle bores into calcium carbonate shells. The economic and environmental, as well as basic biological, significance of the work will make the manuscript of interest to a wide readership.

Several points need clarification.

Lines 134-135. ‘alternately catalyses the dismutation or partitioning of the superoxide radical into either molecular oxygen or hydrogen peroxide’. I think it is more precise to say ‘catalyses the dismutation of two molecules of the superoxide radical into one molecule of oxygen and one molecule of hydrogen peroxide’.

Lines164-165. It is worth pointing out that, based on inhibitor evidence, internal and external carbonic anhydrases as well as plasma membrane bicarbonate transporter are involved in inorganic carbon uptake in submersed thalli of Pyropia yezoensis (Li, Xu and He 2016 Journal of Applied Phycology 28: 491-497). It is therefore possible that the photosynthesis of emersed thalli of Pyropia could transport bicarbonate into the cells despite atmospheric carbon dioxide being inorganic form supplied from the medium.

Lines 200-202. Where does the carbon dioxide come from that generates the protons upon hydration in the proposed mechanism of calcium carbonate dissolution in a photosynthesing alga? F. Garcia-Pichel (2006 Plausible mechanisms for the boring on carbonates by microbial phototrophs. Sedimentary Geology 185: 205-213) points out “that the geochemical consequences of oxygenic photosynthesis should be carbonate precipitation, not dissolution”. Garcia-Pichel (2006) suggests spatial or temporal separation of photosynthesis and respiration as possible explanations, but concludes that neither of these is a likely mechanism of how phototrophs can bore into solid carbonates. Gracia-Pichel (2006) then points out that a then-unexplored mechanism of solid carbonate dissolution is the removal of calcium ions from the aqueous medium where dissolution is occurring, by uptake by the organism, and transport of the calcium inside the organism to the opposite end of the organism, followed by calcium efflux. This requires a calcium efflux pump, and a mechanism of transport along the organism. Subsequent work by Garcia-Pichel, Ramirez-Reinat and Gao (2010 Microbial excavation of solid carbonates powered by P-type ATPase mediated transcellular Ca2+ transport. Proceedings of the National Academy of Science USA 107: 5712-5717), Ramirez-Reinat and Garcia-Pichel (2010 Prevalance of Ca2+ ATPase-mediated carbonate dissolution among cyanobacterial endoliths. Applied and Environmental Microbiology 78: 7-13) and Guida and Garcia-Pichel (2016 Extreme cellular adaptations and cell differentiation required by a cyanobacterium for carbonate excavation. Proceedings of the National Academy of Science USA 113: 21749-21754) provide evidence consistent with the Ca2+ removal hypothesis for carbonate-boring cyanobacteria. While bangiophycean algae are not cyanobacteria(!), this possible mechanism should be mentioned in the context of Pyropia as an internally consistent possible mechanism of carbonate boring. This mechanism requires some adjustment to role of carbonic anhydrase.

Lines 222-232. The natural abundance 13C/12C meaurements of Guida, Bose and Garcia-Pichel (2017 Carbon fixation from mineral carbonates. Nature Communications 18: article 1025) also indicate inorganic carbon from solid carbonate is used in cyanobacterial photosynthesis.

Lines 331-335. Is there a carbon limitation signal in the genome and/or derived proteome of highly expressed proteins (Bragg et al. 2012 Molecular Ecology 21: 2480-2487)?

Supplementary text lines 64-65. A reference is needed, especially since a number of floridiophycean marine and freshwater red algae lack CCMs and rely on diffusive entry of carbon dioxide (Raven, Ball, Beardall, Giordano and Maberly 2005 Algae lacking carbon-concentrating mechanisms. Canadian Journal of Botany 83: 879-890); Stepien 2015 Impacts of geography, taxonomy and functional group of inorganic carbon use patterns in marine macrophytes. Journal of Ecology 103: 1372-1383).

Reviewer #4 (Remarks to the Author):

The manuscript " Nori genome reveals diverse mechanisms of carbon acquisition in the intertidal environment " by Wang et al, uses whole genome assembly of Pyropia yezoensis to infer the interplay between oxidative stress, inorganic carbon availability and life cycle evolution in this commercially important red algal genus.

Unfortunately, the authors often jump to conclusions too quickly without having solid functional understanding and the paper falls short of key descriptions for the methodology employed, especially for the stable isotopes experiment. These two major flaws critically prevent publication in a journal of such a high standard as Nature Communications. I have few major comments:
1. Lack of conclusive functional understanding.
With the intention to elucidate the interplay between Ci availability and life cycle evolution, the authors describe the expansion Carbonic anhydrase isoforms related to their subcellular localization and function but the analysis remains quite superficial (i) with low protein sequence identity reported to characterize different isoforms (line 173, page 7: I wonder how reliable identification of α-CA is based on 37% of pairwise value?? Further, γ-CA role in Ci assmilaiton remains enigmatic as in many cases it is involved in anaplerotic reactions with a related activity contributing to recycle CO2 in the context of photorespiration), (ii) subcellular localization only inferred from sequence analysis while it clearly needs to be done by immunolocalization or CA-GFP fusion to be conclusive; and (iii) lack of functional data such as protein quantification (western blot) or protein activity assay. Their suggestion that expansion of CA isoforms is likely an important factor driving this interplay is relevant but remains inconclusive/not demonstrated in the present version of this manuscript. This would clearly push this paper to the next level, which I believe is critically needed to be published in such a high standard journal.
If the authors can demonstrate this level of functional analysis, they should then clearly summarize it into a schematic diagram overlapping Ci assimilation, gene expression (especially for CA isoforms) and cellular structure of their system as the subcellular compartment/localization is key in the functioning of CCM and Ci assimilation.

The same comment is also true, although to a lesser extent (subcellular localization is less relevant here) for SOD genes, where a functional demonstration is lacking. The authors could at least inform the level of oxidative stress such as ROS under different level of osmotic stress to give more strength to their argument.
2. Description of methods and statistical analysis
While I am no expert in the bioinformatics methods and I am not going to discuss this part, it appears to me that a lot of important information is lacking regarding the osmotic stress and the two Conchocelis-dwelling shells experiments (detection of Ca and pH AND stable isotope labelling experiment)
Please indicate how the dehydration/osmotic stress experiment was run in sufficient details (for instance: field vs lab, number of tank replicates, measurement of dehydration…etc).
Similarly, please indicate with further details how the conchocelis-dwelling shell experiment with the CA inhibitor acetazolamide was run (field vs lab? Tanks volume and replicates? How was the acetazolamide solution prepared (generally needs to be dissolved in basic solution, which could have implications for the experiment)? Was the medium refreshed during the 15 days experiment to ensure acetazolamide concentration was stable? How was the Ca concentration measured?).
Same as above for the stable isotope experiment, please also indicate the level of enrichment of Ca13CO3 used for the incubation. Did you check the bulk level of enrichment (total 13C/12C) in addition to the compounds indicated in line 227-228 (related to this, given that both oxaloacetic acid and malic acid are intermediate, they are likely to have a very rapid turn-over in 13C)
Critically, statistical analyses are missing for both Figure 4C and D with Ca measurement AND 13C enrichment (not even presented with error bars). Please provide results for statistical analyses in a supplementary table.

Minor comments:
Line 44: change radiolabelled as 13C is a stable isotope

The language used in the whole Ci assimilation section is vague and would need to be re-written once subcellular localization/functional information are more conclusive for the different isoforms.

Figure 2C, Fig3B and Fig5C : please indicate the legend/units for x-axis (% of dehydration??)

Fig5a: x-axis: be consistent with the text for gam-specific and spo-specific genes

Nature Communications
May. 22, 2020

Dear Reviewers,

Thank you for reviewing our submission and giving us the opportunity to revise our manuscript. We appreciate the useful comments provided by the reviewers. Here we are submitting a revised version (all edits tracked), with item-by-item responses (regular bold font) to each reviewer comment (in *Italic* text) below.

Comments from reviewer 1:

The authors report the sequencing of the genome of the red alga Pyropia yezoensis. The quality of the genome assembly at chromosomal level seems to be better than published genomes of red alga. The inorganic carbon availability of the Pyropia is analyzed by the high-quality genome. They tested whether the conchocelis Pyropia utilize calcium carbonate of shells as a carbon source by the aid of extracellular carbonic anhydrase (CA). The important data by the isotope supports their hypothesis but the function of CA is not analyzed. Expanded gene families and HGT candidates are analyzed with differential gene expressions and seem to be related to the adaptation to dehydration stress. Those genes that were supported by reliable expressions are interesting and likely include novel findings. Are they clustered into the specific region of chromosomes such as subtelomeres or high GC content? The paper is well written but I have several concerns.

Response: Thank you for this comment. The CA genes are not clustered in specific genomic regions, but rather, are dispersed throughout the three chromosomes. We did a genome synteny analysis using *Pyropia yezonensis, Pyropia haitanensis, and Porphyra umbilicalis*. A high number of large syntenic regions (collinear blocks) were detected between *P. yezonensis* and *P. haitanensis.* A total of 16 out of 24 CA genes were localized in syntenic regions with their corresponding orthologs in *P. haitanensis*. In contrast, few, short regions of synteny were present in the *P. yezonensis* and *Porphyra umbilicalis* comparison. We describe these results in the main text L115-120 and L188-191 and Supplementary Text L63-86.

Detailed remarks:

Line 42: The inorganic carbon of shells is extracellular. What is extracellular release?
Response: We have changed the phrase to "release".

Line 43: CA is carbonic anhydrase?
Response: Yes. We changed CA to carbonic anhydrase to avoid confusion.

Line 43: I think that "This result" should be changed to "this hypothesis".
<u>Response</u>: Thank you. We changed it to "This hypothesis".

Line 49: South Korean?
<u>Response</u>: We changed to Korea.

Line 71: "nori" is used for "Pyropia". In other sentences, "nori" means Pyropia yezoensis.
<u>Response</u>: We agree that "nori" is the product, not the species. We added two words to broaden the definition of *nori* in L51. Now it reads as "The seaweed used most frequently to produce *nori* (sushi wrap), *Pyropia yezoensis*". **We also changed *nori* to *P. yezoensis* throughout the text.**

Line 78: ot?
<u>Response</u>: Sorry for that. We have corrected this word and other misspelled words in the revised text.

Line 95: Is the method for the removal of bacterial sequences indicated?
<u>Response</u>: We have submitted a supplementary methods section that provides detailed information about algal culture, genome sequencing and assembly, gene prediction and annotation, transcriptome data analysis, etc. Removal of bacterial sequences is described in L158-176.

Line 98: Do the long scaffolds have telomeric sequences?
<u>Response</u>: No, there are no telomeric sequences at the ends of three super-scaffolds. In red algae, telomere sequences have been experimentally identified only in the single-celled *Cyanidioschyzon merolae*, as a 9-bp repeat (AATGGGGGG). TTATT(T)AGGG was identified in *Galdieria sulphuraria* using sanger sequencing of Fosmid and BAC libraries. Both of the two red telomeric sequences are different from the typical sequence in plants (TxAyGz)n), implying its sequence divergence in red algae. We also did a genome-wide search of the PacBio data from *P. yezoensis* and other red algal genome assemblies, including *Chondrus* and *Porphyridium,* using the two red algal telomeric repeats as the query and did not find any significant hits. The telomere sequences in red algae may be too highly diverged to be identified using sequence similarity. It is also possible that telomeres are missed during genome sequencing. Future experimental work is needed to characterize the telomere repeats in *P. yezoensis*.

Line 182: Is the protein sequence with the four CA domains supported by transcriptome data?
<u>Response</u>: Yes. PyαCA15 was actively transcribed in both the gametophyte (avg. FPKM=27.2) and the sporophyte (avg. FPKM=25.3). Please see Fig. S16 for the relevant transcriptional heatmap. This gene is also transcribed under dehydration stress.

Line 200: Are the two CAs highly expressed in the chonchocelis?

<u>Response</u>: Yes, the two CA genes exhibited higher transcription in the conchocelis than in the thallus. To make this clear, we have changed the expression into, "Interestingly, we also found three conchocelis-specifically transcribed CAs and another three that exhibit higher expression in the conchocelis than in the thallus (Fig. S16). Two of them (αCA5 and αCA9) were experimentally validated to be secreted proteins (Fig. S13, Fig. S15).**"**

Line 237: How many gene families in the 51 genes?

<u>Response</u>: Among the 51 HGT candidates, seven form expanded gene families.

Line 239: What kinds of bacteria are likely to be related the HGT? Does it include cyanobacteria?

<u>Response</u>: Currently we are not able to infer the exact bacterial donor of HGT-derived genes. The top hit organisms in the NCBI database for each HGT gene are also diverse. Cyanobacterial donors were not considered here. We applied a straightforward filtering method for HGT identification (Nowack, E.C. *et al.*, *Proc Natl Acad Sci U S A*, 2016). *Pyropia* genes that are grouped with cyanobacterial homologs are usually interpreted as endosymbiotic gene transfer (EGT) candidates, thus are not included in the HGT list.

Nowack, E.C. *et al.* Gene transfers from diverse bacteria compensate for reductive genome evolution in the chromatophore of *Paulinella chromatophora*. *Proc Natl Acad Sci U S A* 113, 12214-12219 (2016)

Line 259: Are the eight genes marked in Fig. 5?

<u>Response</u>: Thank you for your suggestion. We indicated the eight genes in the revised Fig. 5 using blue squares.

Line 265: Are found CpG islands in the genome of the high GC content?

<u>Response</u>: A total of 1200 CpG islands were identified in the *P. yezoensis* genome. The GC content ranges from 55% to 82%.

Line 351: How were the samples de-contaminated?

<u>Response</u>: We have submitted a supplementary methods section including detailed information about source of tissues, genome sequencing and assembly, gene prediction and annotation, transcriptome data analysis, etc. Samples de-contamination was described in L18-21.

Line 400: Are the pH values shown?

<u>Response</u>: Yes, the pH value in the medium also increased significantly. The variation in pH values was shown in Fig. S18 and Table S7.

Fig. 4b: What does the red arrow indicate?

<u>Response</u>: The red arrow indicates the conchocelis in the borehole in the shell calcareous matrix. We added this sentence to the Fig. 4b legend.

Comments from reviewer 2:

The authors have sequenced, assembled and analyzed the genome of the red algae P. yezoensis, a member of Bangiophyceae. This species is an important cultivated marine crop, also named Nori, and is consumed worldwide. This alga presents several interesting evolutionary, physiological and ecological characteristics related to its life cycle that involves different types of morphology. These characteristics include tolerance to emersion stress. For example, the authors have investigated the acquisition of inorganic carbon from conchocelis that live in shells, an open and important question. They obtained suggestive evidence that the origin of carbon used in photosynthesis would be the shell through several reactions, the result of several adaptations including HGTs from bacteria, gene expansion of certain families, and a reduction of carbon in DNA and proteins. As mentioned by the authors, two related genomes, P. umbilicalis and P. haitanensis have been sequenced and published.

One of the main issues with this manuscript is that it is difficult to follow and understand certain parts of the work that has been done and the results, especially because of the figures. Since the manuscript contains results that are potentially important in genomics, evolutionary science and molecular ecology, I recommend that the authors get help in professional scientific illustration and writing especially for the figures and supplementary text.

<u>Response</u>: We apologize for these issues. In the revised manuscript, we have improved the text and figures. Missing information is added in the legends for both the main figures and those in the supplement. The supplementary text is also improved and appropriately cited in the main text. In addition, we submitted a supplementary method file to describe the approaches used in this study.

This manuscript presents a longterm effort, including many bioinformatic and physiological analyses. This large amount of work deserves a better presentation with clearer organization. Despite a reasonable number of pages, important data acquisition and analysis must be better explained, many points are too superficial and many important technical details are missing. For example, in Supplementary Information, most Supplementary Figures have very incomplete legends preventing a clear understanding of the analysis. In particular, in Fig S15 which seems to be important, there is no description of the experiment that has been done, no description of the three curves.

<u>Response</u>: In response, we have thoroughly revised the results section to make our message clear to readers. A supplementary method, including detailed information about source of tissues, genome sequencing and assembly, gene prediction and annotation, transcriptome

data analysis, etc. was submitted as an individual file. Figure legends were also revised.

Also, there is no reference to the supplementary text from the main text. Some chapters of the supplementary text look like entire chapters that were extracted from the main text which therefore looks unbalanced in some places. A clear connection between main and supplementary text is really missing.

Response: Some of the results in the Supplementary Text are not related to the main theme, although they comprise important genome structural data, such as the composition of repeat elements and synteny analysis using *P.yezoensis* and the other two available Bangiales genomes. In the revised main text, we cite them as follows: " Nearly one-half of the *P. yezoensis* genome (48.0%) is predicted to be repeat elements, of which long terminal repeat (LTR) retrotransposons are predominant (Supplementary Text, Table S4). The numbers of intact LTR is positively correlated with genome size in red algae (R^2 = 0.9437), explaining genome size growth in *Pyropia* (Fig. S5) and *Gracilariopsis* genome [12]." **in L106-111 and** " To elucidate the evolution of genome structure in Bangiophyceae, we compared gene synteny in *P. yezoensis*, *P. haitanensis* and *Po. umbilicalis*. Long syntenic regions were identified in the two *Pyropia* genomes whereas only a limited amount of synteny was found between *P. yezoensis* and *Po. umbilicalis* (Supplementary Text, Fig. S7). These results suggest that multiple genome rearrangements occurred after the divergence of these two genera." **in L115-120.**

Expanded gene families related to anti-oxidative function, including the lipoxygenase genes and tyrosinase genes, exhibit a similar pattern as SOD genes. These are transcribed in the gametophyte and up-regulated under osmotic stress. To avoid repetition, we have moved this part to supplementary text. To make a "clear connection", we appropriately cited them in this revised main text as L165-172: " In addition to SOD, several other oxidative stress related OGs, including lipoxygenase, catalase, and tyrosinase have expanded gene numbers in *Pyropia* species (Fig. 2a). Members of these gene families exhibit similar transcriptional patterns to SODs, with up-regulation in a specific life-history stage and high expression of gam-specifically transcribed genes under osmotic stress (Supplemental Text, Fig. S10-S11). The conserved response across these different gene families hints at the evolution of a regulatory mechanism in Bangiales for life in the intertidal zone."

Apparently, the authors have generated a high quality assembly at the chromosome level. This is a very important effort for the community, and I hope this assembly will be integrated into international bioinformatics platforms. It is a bit surprising that the authors did not profit from this important achievement for deeper evolutionary and comparative analysis of genome structures. Most, or even all, of the analyses presented in this manuscript are based on gene sequences and are not linked to the structure of the genome. Maybe leverage the genome structure could help to better illuminate some questions raised by the authors.

Response: Thank you for this making this recommendation. To study the evolution of genome structure in Bangiophyceae, we compared gene synteny from *Pyropia yezoensis*,

Pyropia haitanensis **(Cao M, et al., 2019) and *Porphyra umbilicalis* (Brawley SH, et al., 2017) using MCScanX (Wang Y et al., 2012). Large syntenic regions were identified between the two *Pyropia* species,a whereas only a few and short regions were found between *P. yezoensis* and *Porphyra umbilicalis*, as indicated in Fig. S7. This analysis was described in the supplementary Text L63-86. Moreover, we investigated the location of CA genes. These are dispersed in the three chromosomes in *P. yezoensis* and well conserved in terms of position and sequence similarity in *P. haitanensis* (Fig. S12).**

We will reserve a more comprehensive future study on the inter-species comparative analysis of genome structure and function.

Wang Y, Tang H, DeBarry JD, Tan X, Li J, Wang X, Lee T-h, Jin H, Marler B, Guo H *et al*: **MCScanX: a toolkit for detection and evolutionary analysis of gene synteny and collinearity**. *Nucleic Acids Research* 2012, **40**(7):e49-e49.

Cao M, Xu K, Yu X, Bi G, Liu Y, Kong F, Sun P, Tang X, Du G, Ge Y *et al*: **A chromosome-level genome assembly of *Pyropia haitanensis* (Bangiales, Rhodophyta)**. *Molecular Ecology Resources* 2020, **20**(1):216-227.

Brawley SH, Blouin NA, Ficko-Blean E, Wheeler GL, Lohr M, Goodson HV, Jenkins JW, Blaby-Haas CE, Helliwell KE, Chan CXJPotNAoSotUSoA: **Insights into the red algae and eukaryotic evolution from the genome of *Porphyra umbilicalis* (Bangiophyceae, Rhodophyta)**. 2017, **114**(31):E6361.

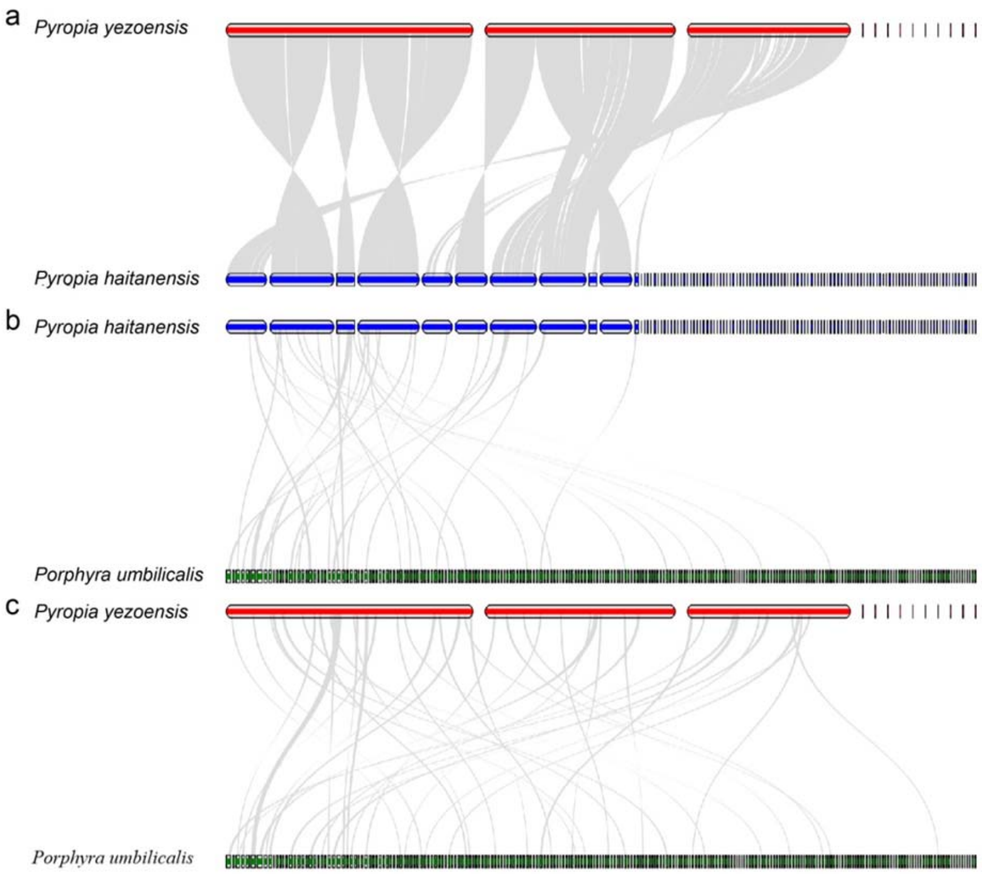

Fig. S7. Syntenic regions in Bangiales genomes. The wide horizontal lines in red, blue, and green represent the assembled scaffolds in *P. yezoensis*, *P. haitanensis* and *Po. umbilicalis.* Gray areas represent collinear blocks identified by MCScanX between any two genomes. (**a**) Collinear blocks between *P. yezoensis* and *P. haitanensis*. (**b**) Collinear blocks between *P. haitanensis* and *Po. umbilicalis.* (**c**) Collinear blocks between *P. yezoensis* and *Po. umbilicalis*.

The discussion about high GC as a relic of an ancient adaptation to carbon limitation in the Permo-Carboniferous era is interesting but not convincing. The rate of GC percent rapidly evolves in genomes, especially on third bases of course. For example, in other eukaryotes where recurrent HGT has been demonstrated, the GC% of more ancient HGT genes are closer to the GC% of the host genome compared to more recent HGTs.

Response: We agree that we should be cautious when discussing the link between high GC content and ancient carbon limitation because GC content variation can result from multiple factors. We have changed/added some text in L428-434: "The strong bias toward a reduced frequency of carbon-containing amino acids in Bangiales genomes is consistent with (but does not prove) the hypothesis of carbon-limitation driving the evolution of Bangiales. It is clear that other factors may impact GC content such as genome size increase driven by transposon expansion (e.g., in grasses[46]) and high levels of intra-genic recombination (i.e., GC-based gene conversion; gBGC) that can act independently from selection to create codon

usage bias [47]. ".

Overall, I am inclined to follow the authors in their conclusions but they need to significantly improve figures, methods and text. There is ample matter for a clear and logical presentation for this manuscript. The abstract must be reshaped to better connect results into clear messages.
Response: Thank you for these comments. As for the areas that were suggested to "need significant improvements", we have in the revised manuscript thoroughly revised/ addressed the potentially confusing points that were kindly raised by this reviewer. The abstract was also rephrased to clarify our message.

In summary, this is a potentially interesting contribution. My major criticism is that the presentation (text, figures, logical flow) is of insufficient quality and must be greatly improved before publication.
Response: We have done an extensive revision on the main texts and figures according to the constructive suggestions from you and the other reviewers, as highlighted in the text. We are greatly thankful to your constructive suggestions.

Detailed points:

• The subtitle "Orthogroup analysis" is too technical.
Response: We changed the subtitle into "Expanded orthogroups and their specific transcription in particular life stage" to present the clear message better.

• Genome Assembly: did authors estimate sequence quality at the nucleotide level as it is not clear if they used Illumina reads to correct errors?
Response: Yes, we generated a total of 10.4 Gb (~100X of depth) paired-end data and 5.4 Gb of mate-pair data with insert size of 5 Kbp using the Illumina platform (Table S1). After filtering out low quality reads and reads with adaptors, these data were used to correct potential InDel and base substitution errors in the draft assembly with the Quiver algorithm from SMRT Analysis v2.3.0 (Chin, 2013). This genome polishing process was described in the supplementary method which we submitted as an individual file in the revision.

• There is not enough information about gene calling methodology.
Response: Our apology for the absence of detailed information of methodology. We submitted a supplementary method as an individual file in this revision. The gene calling section was described in L179-187 and briefly cited in the main text (L459-461).

• Line 116: "significantly expanded": is there any statistics supporting this affirmation?
Response: Yes, there's statistical analysis in CAFE program when determining the expanded and contracted OGs. The *p*-values for CA and anti-oxidative related gene

families are now listed in fig. 3 and fig. 2 respectively.

• Line 129: the terms "spo-specific" and "gam-specific" are not appropriate since the corresponding genes are not specific of these life history stages.

Response: These genes exhibited phase-specific expressions. To avoid misunderstanding, we changed the two terms into "spo-specifically transcribed" and "gam-specifically transcribed".

• Line 137: ROS-induced-cell is not defined.

Response: Sorry for the confusion, we changed the phrase into " the amelioration of cell damage induced by ROS".

• Line 144: The 2 genes that are highly expressed in Sporophyte are not shown in figure 2b (py00127.t1, py01435.T1). This must be clarified.

Response: The two genes were included in the revised Fig. 2b.

• Line 180-181. References are needed.

Response: References are added.

• Line 185: The authors list 6 genes with high or specific expression in the thallus, but Fig S14 that is referred to in this context shows more than 6 genes (ex AlphaC13). Why? This is puzzling.

Response: Sorry for the misleading presentation of Fig S14. There is a total of 24 CA genes annotated in the *P. yezoensis* genome. We presented their transcription levels in the two life history stages in Fig S14. Among them, the six CA genes listed in L185 exhibited specific or higher transcription in gametophyte stage. To clarify this point, we added the following sentences in Fig S14 legend (Fig. S16 in this revision): "Fig. S16. The transcriptional patterns of CA genes in the two life history phases of *P. yezonensis*. The FPKM values of CA genes (n=4) in the gametophyte and sporophyte were used to generate the heat map after z-score normalization. CA genes exhibiting specific transcription in the gametophyte stage (defined as "gam-specifically-transcribed" genes in main text) are indicated by the red stars and spo-specifically-transcribed CAs are indicated by the green stars. Those genes with 2-fold higher of transcriptional level in one stage when compared to the other were indicated as blue stars."**.**

• Line 193 and Fig 3b: There is no explanation of the cellular compartment indicated in the figure and no methodology explained to predict "N terminal plastid targeting sequence".

Response: We added the following sentences to describe our method of predicting the sub-cellular localization of relevant proteins in L463-466: " Sub-cellular localization of *Pyropia* proteins were predicted using PredAlgo which is a multi-subcellular localization prediction tool designed for algae [56]. Trans-membrane helixes were predicted using TMHMM (v2.0) [57]available at http://www.cbs.dtu.dk/services/TMHMM/." **In the Fig. 3b legend, we inserted this sentence to explain the cellular compartments:** "The subcellular localization of

CA isoforms and HCO_3^- transporters are based on PredAlgo prediction and transient expression of GFP fusion proteins in tobacco. α-CA7 that are predicted to harbor transmembrane helixes by TMHMM are placed across the chloroplast membrane. Plastid-targeting CAs might function in either chloroplast stroma or thylakoid (green dashed square) where RuBisCO is localized.**".**

• Line 194- 195: As explained previously, the Fig S15 misses all information required to understand how it supports the sentence.

<u>Response</u>: Our apology and we added informative legends for all the supplementary figures in this revision. As for Fig S15 (Fig. S18), the legend reads as: "Fig. S18. PH values in the vicinity of conchocelis-dwelling shells w/o CA or P-type ATPase inhibited. X-axis stands for the duration of time (days) after shells were placed into the medium. Y-axis stands for the pH values. The 0-time value was 7.9. Control medium (with blank shell), growth medium (with conchocelis-dwelling shells), CA inhibitory medium (acetazolamide was added into growth medium) and transport inhibitory medium (vanadate was added into growth medium) are plotted in grey, red, blue and yellow. The error bars show the standard deviations (n = 3). P values were calculated as described in Table S7. The two asterisks indicating p<0.01 for CA inhibitory medium were placed below the blue plot, while the ones for transporter inhibitory medium were placed above the yellow plot.**".**

• Lines 205-212: this experiment is really interesting. But in Fig 4c, authors must at least use the same terms they used in the main text. Do not name the curves as "acetazolamide", "growing conchocelis" and "blank shell", this is not relevant. For example use "control" , "inhibitory"….

<u>Response</u>: As advised, we changed the terms in Fig 4c accordingly.

• Line 229: Fig 4d instead of 5d I suppose.

<u>Response</u>: Corrected accordingly.

• The HGT analysis is really interesting. Is it possible to estimate the number of different events of transfer and to date them?

<u>Response</u>: This would be an interesting analysis if we had a larger number of Bangiales genomes available to address HGT gain/loss scenarios. A recent paper in eLife regarding HGT in *Galdieria* species (eLife 8:e45017) better addresses this issue.

• Statistical tests are needed to support comparisons of frequencies of G/C to A/T.

<u>Response</u>: We performed a paired-samples t-test to support the comparisons. The *p* value was added in the sentence.

• The section about HGT requires a conclusion.

<u>Response</u>: This should suffice, we think. "Combining the identification of HGT-derived CA gene for CCM, this indicates the important role of HGT in allowing *Pyropia* to thrive in its

stressful habitat and overcome C_i limitation. This hypothesis is supported by the thallus specific gene expression patterns of HGT-derived genes."

How is the pattern of high GC related to the biology of the species here? Is it related to the adaptation of Ci acquisition adaptation?

Response: We looked at the average GC contents of functional groups as defined by the associated Gene Ontology Slim terms in biological process. Slight variations in average GC% were observed among the top 15 largest functional groups. The multicellular organization process and stress response process are the two highest groups (please see Fig. S19b below). Significant relationship between the high GC and the adaptation of Ci acquisition adaptation is not observed.

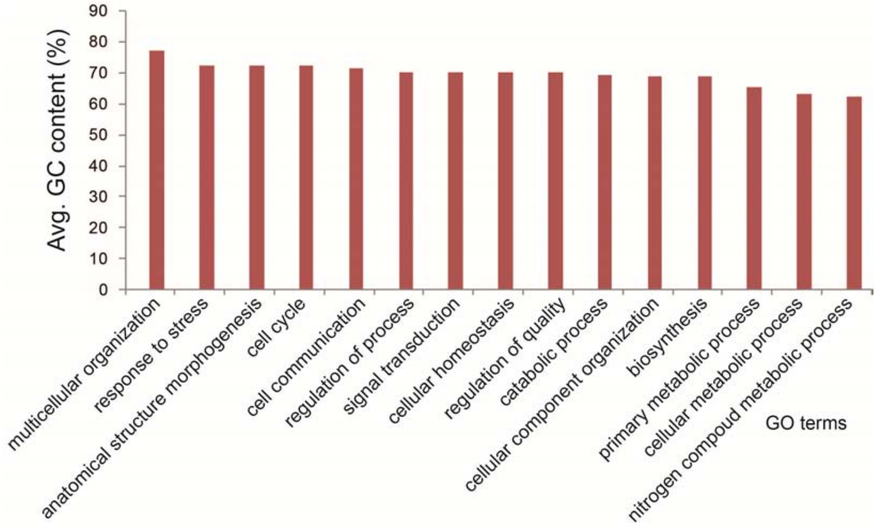

Fig. S19 (**b**) Average GC content of functional groups in the *P. yezoensis* genome. GC content of genes related to the corresponding Gene Ontology Slim terms in biological process are calculated. The top 15 functional groups (including the largest number of genes) are shown.

• Fig S4 indicates a physical map from Hi-C. How was it produced and used in the assembly? The authors must provide more technical information. The algorithm to integrate the optical map and Hi-C is not described.

Response: We submitted a supplementary method as an individual file including a detailed description of genome assembly. The generation of Hi-C data is described in L49-56:" Additionally, 4 µg purified high molecular weight genome DNA was digested by BspQI, then was labeled by fluorescence and counterstained for constructing Bionano librariy. For the Hi-C library, chromatin was fixed in placewith form aldehyde in the nucleus, and then the fixed DNA was extracted and digested with MboI. Sticky ends were biotinylated and proximity ligated to form chimeric junctions that were enriched for and then physically sheared to a size of 300-500 bp. Chimeric fragments representing the original cross-linked long-distance physical interactions were then processed into paired-end sequencing libraries.**". The use in genome assembly is described in L134-143: "** Scaffolding of the contigs with optical mapping was performed using

the Irys optical mapping technology (BioNano Genomics). Labeled single molecules above 150 kb in size were used to produce single-molecule maps and assemble maps into a genome map by the IrysView (BioNano Genomics, https://bionanogenomics.com/support/software-downloads/) software package. A proximity-guided assembly was further performed using Hi-C technology. The paired-end reads were uniquely mapped onto the draft assembly contigs. Three chromosome clusters were generated. Further scaffolding within chromosome cluster each was done using 3D-DNA software (editor_repeat_coverage=50).**".**

• Line 365: FPKM is not introduced and there is no reference for the cufflink program.
Response: The full name of FPKM and the reference for cufflink program are added.

• Line 367: Calibration of Augustus is not explained.
Response: In this revision, we submitted a supplementary method that provides a detailed description of methods used in genome sequencing, assembly, gene prediction etc. Methodology in gene prediction was described in L178-188.

• Fig S1, S2, S5 to S9, S13 to S16: Legends are too short. More explanation and description are needed.
Response: We added complete legends for all the supplementary figures.

• Line 395: define OG
Response: OG was defined as "orthogroup" in the first place where it's used in L125.

• Fig 1: It would be interesting to indicate ploidy of each state, where meiosis occurs.
Response: Thank you for your advice. The ploidy of each stage was added in Fig. 1 and explained in the legend.

• Fig 2: This figure is central to the main results but poorly explained, the important information is not underlined. Importantly, there is no statistics provided to support the figure. In panel a, authors should mention the origin of the tree, it would be interesting to annotate the nodes in the tree by genomic, biological or physiological information. Also, it would be useful to add in this panel the expression folding in addition to gene number expansion. Fig 2b is meaningless. The unit of the Y axis is not explained, it is not clear that the X axis represents genes. This figure does not show the expression over the life cycle as written in the title and in the legend since only 2 stages are shown: sporophyte conchocelis and gametophyte thallus.
Response: The dendrogram in Fig. 2a was generated based on the phylogenetic tree constructed using the 424 single-copy orthologous gene sets identified from *P. yezoensis* and 17 other published algal genomes. This description was added in legend. *P*-values for each expanded orthogroup was added at the bottom of this panel. The y-axis represents expression levels in individual life history phases (FPKM values). The expression of "over life cycle" in the title was changed into "in two life history phases".

• Fig 3: Stramenopile, not "Stramenophiles" I suppose. In panel b the dendrogram is not explained. In panel a, the number of alpha CA does appears higher in Pyropia and Porphyra than in most branches but not significantly higher than in rice for example.

Response: The dendrogram was explained in legend as:" The dendrogram was constructed as described in Fig. 2. **".**

We noticed that more CA genes were identified in land plants. The CA expansion in land plants was previously discussed in the reference below (DiMario, R.J. et al., 2018). We agree with their explanation that gene copies and their individual metabolic roles have likely changed during the independent evolution of land plants, possibly in order to adapt to the drop of atmospheric CO_2 level.

DiMario, R.J., Machingura, M.C., Waldrop, G.L. & Moroney, J.V. The many types of carbonic anhydrases in photosynthetic organisms. *Plant Science* **268**, 11-17 (2018)

• Fig 4: Panel c: The 3 curves must be explained in the legend, time is not mentioned (X axis). Panel d needs more explanations. How many replicates were done? Standard deviation is missing.

Response: We inserted the following sentences to explain the plots:" (**c**) The variation of Ca^{2+} release from conchocelis-dwelling shell w/o carbonic anhydrase inhibitors. Ca^{2+} concentrations in control medium, growth medium and inhibitory medium were plotted in grey, red and blue respectively.**". For the ^{13}C assay in panel d, we repeated this experiment with three more biological replicates (explained in L511-519 in method section) and revised this figure based on the updated results. Standard deviations were also added.**

• Fig 5: This figure shows the importance of HGT genes in the repertoire of gam-specific and spo-specific genes. Functions of these genes are indeed biased, but panel c is not demonstrative. In panel c, HGT genes display a range of types of expression for different stress conditions, but GAM specific genes do not seem to hold any peculiarity. Maybe a comparison of HGT versus non HGT genes would be more demonstrative.

Response: Thank you for this comment. We attempted to compare the temporal expression dynamics of HGT versus non-HGT genes. Because the number of non-HGT genes (> 8,000) are much higher than HGT, it is difficult to show the predominance of gam-specifically transcribed HGTs among those with up-regulated expression under osmotic stresses. Therefore, to make the information clearer in fig. 5c, we use a red square to mark HGT genes that were up-regulated under osmotic stress.

• More methodological information about HGT detection must be provided as it is an important analysis.

Response: We added the following sentences to explain the methodology of HGT detection in Supplementary method L223-249.

• Expansion of CA: did the authors investigate chromosomal locations of these copies in comparison to other species? Is there interesting information allowing an hypothesis about evolution of this function?

Response: Yes, we did this. CA genes exhibited a dispersed location in the three chromosomes of *P. yezoensis*. Interestingly, αCA8 and αCA9 are adjacent to each other in scaffold S2, with a 3.7kb interval. Their protein sequences are highly similar (Identity= 95%), except for the N-terminal 70 aa. Orthologs of the two CA genes are also found localized closely in the *P. haitanenesis* genome. *Po. umbilicalis* orthologs of αCA8 and αCA9 are localized at the ends of contig KV919297.1 and KV918987.1 respectively. It is possible that the two contigs were not well assembled. Moreover, αCA9 was transcriptionally active in *Pyropia* conchocelis, whereas transcripts for αCA8 were not detected. Thus, we postulate that αCA8 and αCA9 may originate from a gene duplication event that occurred in the ancestor of the two genera, followed by transcriptional silencing of αCA8 in *P. yezoensis* due to yet unknown reasons.

• The analysis about SOD expansion is very interesting however figures should be improved and more conclusive. It is curious that the authors do not comment at all in the main text their analysis of lipoxygenase and tyrosinases.

Response: We improved Fig. 2 according to your advice. Expanded gene families related to anti-oxidative function, including the lipoxygenase genes and tyrosinase genes, exhibit a similar pattern as SOD genes. These are transcribed in the gametophyte and up-regulated under osmotic stress. We moved this part to supplementary text to avoid repetition. We cited them in this revised main text as L165-172: " In addition to SOD, several other oxidative stress related OGs, including lipoxygenase, catalase, and tyrosinase have expanded gene numbers in *Pyropia* species (Fig. 2a). Members of these gene families exhibit similar transcriptional patterns to SODs, with up-regulation in a specific life-history stage and high expression of gam-specifically transcribed genes under osmotic stress (Supplemental Text, Fig. S10-S11). The conserved response across these different gene families hints at the evolution of a regulatory mechanism in Bangiales for life in the intertidal zone."

Comments from reviewer 3:

The manuscript uses a genomic approach to investigate mechanisms of carbon acquisition of an economically and ecologically important marine macroalga, with emphasis on the intriguing problem of how and why the diploid phase in the life cycle bores into calcium carbonate shells. The economic and environmental, as well as basic biological, significance of the work will make the manuscript of interest to a wide readership.

Several points need clarification.

Lines 134-135. 'alternately catalyses the dismutation or partitioning of the superoxide radical into either molecular oxygen or hydrogen peroxide'. I think it is more precise to say 'catalyses the dismutation of two molecules of the superoxide radical into one molecule of oxygen and one molecule of hydrogen peroxide'.

<u>Response</u>: Thank you, changed accordingly.

Lines164-165. It is worth pointing out that, based on inhibitor evidence, internal and external carbonic anhydrases as well as plasma membrane bicarbonate transporter are involved in inorganic carbon uptake in submersed thalli of Pyropia yezoensis (Li, Xu and He 2016 Journal of Applied Phycology 28: 491-497). It is therefore possible that the photosynthesis of emersed thalli of Pyropia could transport bicarbonate into the cells despite atmospheric carbon dioxide being inorganic form supplied from the medium.

<u>Response</u>: Thanks for this important point. The reference was added to the revised text.

Lines 200-202. Where does the carbon dioxide come from that generates the protons upon hydration in the proposed mechanism of calcium carbonate dissolution in a photosynthesing alga? F. Garcia-Pichel (2006 Plausible mechanisms for the boring on carbonates by microbial phototrophs. Sedimentary Geology 185: 205-213) points out "that the geochemical consequences of oxygenic photosynthesis should be carbonate precipitation, not dissolution". Garcia-Pichel (2006) suggests spatial or temporal separation of photosynthesis and respiration as possible explanations, but concludes that neither of these is a likely mechanism of how phototrophs can bore into solid carbonates. Gracia-Pichel (2006) then points out that a then-unexplored mechanism of solid carbonate dissolution is the removal of calcium ions from the aqueous medium where dissolution is occurring, by uptake by the organism, and transport of the calcium inside the organism to the opposite end of the organism, followed by calcium efflux. This requires a calcium efflux pump, and a mechanism of transport along the organism. Subsequent work by Garcia-Pichel, Ramirez-Reinat and Gao (2010 Microbial excavation of solid carbonates powered by P-type ATPase mediated transcellular Ca2+ transport. Proceedings of the National Academy of Science USA 107: 5712-5717), Ramirez-Reinat and Garcia-Pichel (2010 Prevalance of Ca2+ ATPase-mediated carbonate dissolution among cyanobacterial endoliths. Applied and Environmental Microbiology 78: 7-13) and Guida and Garcia-Pichel (2016 Extreme cellular adaptations and cell differentiation required by a cyanobacterium for carbonate excavation. Proceedings of the National Academy of Science USA 113: 21749-21754) provide evidence consistent with the Ca2+ removal hypothesis for carbonate-boring cyanobacteria. While bangiophycean algae are not cyanobacteria(!), this possible mechanism should be mentioned in the context of Pyropia as an internally consistent possible mechanism of carbonate boring. This mechanism requires some adjustment to role of carbonic anhydrase.

<u>Response</u>: Many thanks for your valuable recommendation of Garcia-Pichel's work in cyanobactria. It's exciting to see that most of our observations in the *Pyropia* conchocelis

are consistent with the model they have proposed, including the elevated Ca^{2+} concentration and pH value in external liquid medium, and the incorporation of carbon from $CaCO_3$. In this revision, we also added another result confirming the involvement of P-type Calcium ATPase, further supporting the model. This inhibitory experiment was previously done in parallel with the CA inhibitory experiment. This remarkable similarity between the two phototrophs implies that the mechanism underlying their excavation of mineral substrate may be evolutionarily conserved. Extensive studies on the specific nature of the calcium efflux pump, its cellular distribution and evolution, are needed to fully understand the evolutionary origin of this mechanism. In our study, we also proposed the involvement of extracellular CA in digesting $CaCO_3$. In the cyanobacterial model, Ca^{2+} originated from spontaneous ionization of $CaCO_3$ and was then passively diffused into the apical cell. We agree on this point. However, we also suggested another origin of Ca^{2+} from the proton attack on $CaCO_3$ and the proton generated from aquatic CO_2 hydration catalyzed by extracellular CA (there is free CO_2 in ocean water, although scarce in the microenvironment of boreholes). In addition, extracellular CA might also function in the combination of spontaneously ionized CO_3^- and a proton pumped out coupled with Ca^{2+} transportation. Therefore, we proposed a modified working model in this revision (Fig. 4e) with the inclusion of CA function.

Variation in Ca^{2+} concentration in the growth medium in the presence of vanadate was plotted in Fig 4c. In the revised main text, we described the inhibitory experiment of P-type ATPase in L263-270: "Previous studies in euendolithic cyanobacterium *Mastigocoleus testarum*[28] showed that the released Ca^{2+} from the carbocite passively diffused into the apical cell, moved thereafter from cell to cell, and was finally pumped into the liquid medium by the distal cell through a P-type calcium ATPase. In *Pyropia*, two genes encoding P-type calcium ATPase were identified and both of them exhibited high expression in the conchocelis. We added vanadate, the specific inhibitor of P-type cation-transporting ATPase, into the growth medium and found that Ca^{2+} extrusion was strongly inhibited, implying a pivotal role for the transporters in this process (Fig. 4c)." **Discussion of the working model in *Pyropia* conchocelis is shown in L389-408, as showed in Fig. 4e.**

Lines 222-232. The natural abundance 13C/12C meaurements of Guida, Bose and Garcia-Pichel (2017 Carbon fixation from mineral carbonates. Nature Communications 18: article 1025) also indicate inorganic carbon from solid carbonate is used in cyanobacterial photosynthesis.

<u>Response</u>: Thanks for this comment, this key reference was added.

Lines 331-335. Is there a carbon limitation signal in the genome and/or derived proteome of highly expressed proteins (Bragg et al. 2012 Molecular Ecology 21: 2480-2487)?

<u>Response</u>: We applied the method described in the cited reference to calculate the average number of carbon atoms in protein sequences of protein-coding genes. Gene with FPKM >100 in every condition included in this study were defined as highly expressed. The average number of carbon atoms of highly expressed proteins is 4.65, higher than the

remaining proteins (4.48), but not statistically significant (Please see the figure below).

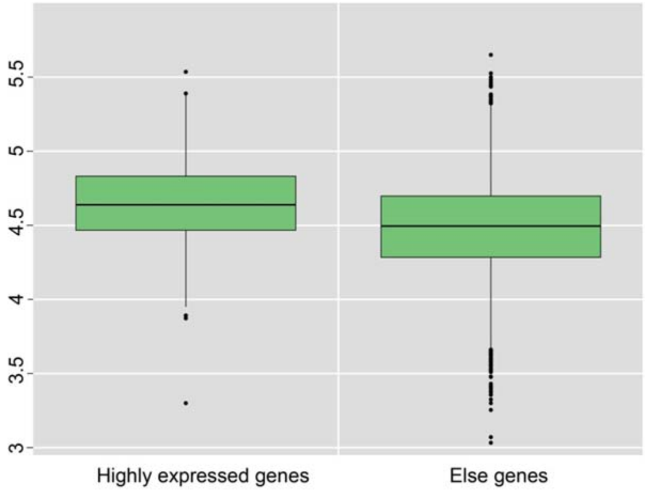

Supplementary text lines 64-65. A reference is needed, especially since a number of floridiophycean marine and freshwater red algae lack CCMs and rely on diffusive entry of carbon dioxide (Raven, Ball, Beardall, Giordano and Maberly 2005 Algae lacking carbon-concentrating mechanisms. Canadian Journal of Botany 83: 879-890); Stepien 2015 Impacts of geography, taxonomy and functional group of inorganic carbon use patterns in marine macrophytes. Journal of Ecology 103: 1372-1383).

Response: Thank you. The references were added.

Comments from reviewer 4:

The manuscript " Nori genome reveals diverse mechanisms of carbon acquisition in the intertidal environment " by Wang et al, uses whole genome assembly of Pyropia yezoensis to infer the interplay between oxidative stress, inorganic carbon availability and life cycle evolution in this commercially important red algal genus.

Unfortunately, the authors often jump to conclusions too quickly without having solid functional understanding and the paper falls short of key descriptions for the methodology employed, especially for the stable isotopes experiment. These two major flaws critically prevent publication in a journal of such a high standard as Nature Communications. I have few major comments:

Response: Thank you for raising these issues. In the revised manuscript, we submitted a supplementary method describing in detail the methods used in genome sequencing, read filtering, assembly, gene annotation, phylogenomic analysis, differential expression analysis, Ca^{2+} detection in the conchocelis, and the stable isotope labeling etc. For the stable isotope experiment that you are concerned about, we also added the following sentences in main text (L511-519) to make it clear: " For the ^{13}C labeling experiment, 0.3g free conchocelis was

cultured in 200ml artificial sea water with 0.3g ^{13}C-labeled $CaCO_3$ (99 atom%, Sigma-Aldrich), in 20 ± 1 °C under 50-60 μmol photons m^{-2} s^{-1} (12:12 light:dark cycle) with three rounds of gentle shaking every day. Control samples of equal weight were co-cultured with ^{12}C- $CaCO_3$. Three biological replicates were done with these experiments. After 20 days of cultivation, the abundance of ^{13}C atom in the organic acids related to the TCA cycle were quantitatively analyzed using GC-MS based on the retention time, EI mass spectrum of corresponding standards and matching to the NIST library, as described in Mulat et al. (2015) [66]"

For the lack of "solid functional understanding", we have made the following revisions: 1, We determined the sub-cellular localization of four CA isoforms through transient expression of GFP fusion proteins in tobacco, including three extracellular CA isoforms and one cytosolic CA. A schematic diagram of CCM components (Fig. 3b) was also constructed based on the predicted and experimentally validated localizations of CA isoforms. 2, We measured the enzyme activity assay for extracellular CA in conchocelis, further supporting its presence (L243 in main text). 3, To further understand the molecular mechanism underlying the excavation of shell matrix of conchocelis, we added the inhibitory experiment of P-type ATPase and confirmed its involvement in pumping Ca2+ into external liquid medium. A model modified from work done in cyanobacteria is proposed in Fig. 4e.
We agree that the physiological functions of each CA isoform remain to be determined through genetic manipulation, immunolocalization and protein quantification. We also attempted to prepare antibodies for transcriptionally active CA isoforms in this revision, but have not yet been successful.

1. Lack of conclusive functional understanding.
With the intention to elucidate the interplay between Ci availability and life cycle evolution, the authors describe the expansion Carbonic anhydrase isoforms related to their subcellular localization and function but the analysis remains quite superficial (i) with low protein sequence identity reported to characterize different isoforms (line 173, page 7: I wonder how reliable identification of • -CA is based on 37% of pairwise value??
<u>Response</u>: We identify CA genes via the following method: 1, Search *Pyropia* genome and predicted gene models using tblastn and blastp respectively, with published CA protein sequences of algae (*Chlamydomonas*, *Phaeodactylum*, etc.) and plants as query (e-value = 1e-05); 2, Candidate genes from step 1 are blasted against NR database to confirm their possible functions; 3, Blast against CDD database in NCBI to search for conserved CA domains and amino acids; 4, Annotate the gene families in Pfam database. Besides, relatively low sequence identities in αCA isoforms are also observed in other algae, eg. *Phaeodactylum* αCA-1 (Phatr3_J35370.p1) and αCA-3 (Phatr3_J55029.p1).

Further, -CA role in Ci assmilaiton remains enigmatic as in many cases it is involved in anaplerotic reactions with a related activity contributing to recycle CO2 in the context of

photorespiration),

__Response__: **In *Pyropia*, we also identified several CAs that are predicted to be localized in mitochondria. Four of them are expressed in both the thallus and the conchocelis (Fig. 3b). These enzymes are likely involved in photorespiration. Additional studies are needed to determine with certainty their specific physiological functions.**

(ii) subcellular localization only inferred from sequence analysis while it clearly needs to be done by immunolocalization or CA-GFP fusion to be conclusive;

__Response__: **We agree that determination of the sub-cellular localizations of CA isoforms is critical to understand their role in Ci assimilation. Due to the lack of genetic engineering tools in red algae (including *Pyropia*), we used transient gene expression in tobacco which has previously been shown to allow determination of the subcellular localization of *Pyropia* proteins. Following the experimental procedure described in Supplementary method L251-269, we cloned the cDNA sequences of seven CA genes (amplification of cDNA was difficult in *Pyropia* due to high GC content) to construct CA-GFP fusion expressing cassettes. The cassettes were then transformed into tobacco cells by agroinfection. Four of them (α-CA5, α-CA9, α-CA14 and β-CA4) were successfully expressed and displayed clear localization in extracellular matrix or cytoplasm as observed under fluorescence microscope (Fig. S15). The results were described in L208-211: "** The extracellular localization of αCA5 and αCA9, as well as the cytoplasmic localization of βCA4, were confirmed using transient expression of GFP fusion proteins in tobacco (Fig. S15). Although αCA14 was predicted to be a cytosolic proteins, it was clearly localized to the extracellular matrix in tobacco. **". We also attempted to prepare antibodies for transcriptionally active CA isoforms in this revision, but have not yet been successful.**

PredAlgo is a sub-cellular localization prediction tool designed for algae. Given that the same derivation from primary endosymbiosis of red and green algae, we employed PredAlgo in this study. Among the four CAs with localization determined by transient expression in tobacco, three (the extracellular α-CA5 and α-CA9, cytoplasmic β-CA4) had consistent results with the computational prediction by PredAlgo. This suggested that PredAlgo was a good protein localization predictor for red algae. In addition, PredAlgo was reported to give extremely high accuracy for plastid-targeting proteins (90%, Mackinder et al. 2017). Therefore, the localization of nine CA isoforms were determined with a high probability to be chloroplast, as predicted by PredAlgo.

Mackinder et al., A Spatial Interactome Reveals the Protein Organization of the Algal CO2 Concentrating Mechanism, Cell. 2017 September 21; 171(1): 133–147.e14. doi:10.1016/j.cell.2017.08.044.

Based on both the computational prediction by PredAlgo and the transient expression tobacco of CA isoforms, we drew a schematic diagram of CCM in Pyropia (Fig. 3b).

and (iii) lack of functional data such as protein quantification (western blot) or protein activity

assay. Their suggestion that expansion of CA isoforms is likely an important factor driving this interplay is relevant but remains inconclusive/not demonstrated in the present version of this manuscript. This would clearly push this paper to the next level, which I believe is critically needed to be published in such a high standard journal.

Response: Thank you for your suggestion. In this revision, we detected the extracellular CA activities in conchocelis, as described Result section L243 and Supplementary method section L271-281. Unfortunately, we were unable to generate monoclonal/polyclonal antibodies of any CA isoforms for protein quantification and immunolocalization in the current work.

If the authors can demonstrate this level of functional analysis, they should then clearly summarize it into a schematic diagram overlapping Ci assimilation, gene expression (especially for CA isoforms) and cellular structure of their system as the subcellular compartment/localization is key in the functioning of CCM and Ci assimilation.

Response: Thank you for your advice. We have made a schematic diagram of the CCM in *Pyropia* (Fig. 3b). The subcellular localization of CAs and bicarbonate transporters are based on computational prediction using PredAlgo and transient expression of GFP fusion proteins in tobacco. Transcriptional levels in the thallus and conchocelis, as well as the temporal transcriptional patterns under osmotic stress are presented in this diagram.

The same comment is also true, although to a lesser extent (subcellular localization is less relevant here) for SOD genes, where a functional demonstration is lacking. The authors could at least inform the level of oxidative stress such as ROS under different level of osmotic stress to give more strength to their argument.

Response: Accumulation of ROS and elevated SOD activity in *Pyropia* thalli during dehydration, hypersaline, high/low temperature etc. were previously reported (please see the references below). Given the extensive work we have done to revise the manuscript, we did not attempt these experiments, although we recognize their potential value.

Wenjun Wang *et al*. Effects of periodical drying and non-drying on nutrient content and desiccation tolerance of an intertidal Pyropia yezoensis strain subject to farming conditions. Journal of Applied Phycology (2019) 31:1897–1906

Wenlei Wang *et al.* Regulatory mechanisms underlying the maintenance of homeostasis in Pyropia haitanensis under hypersaline stress conditions, Science of the Total Environment 662 (2019) 168–179

2. Description of methods and statistical analysis

While I am no expert in the bioinformatics methods and I am not going to discuss this part, it appears to me that a lot of important information is lacking regarding the osmotic stress and the two Conchocelis-dwelling shells experiments (detection of Ca and pH AND stable isotope

labelling experiment)
Please indicate how the dehydration/osmotic stress experiment was run in sufficient details (for instance: field vs lab, number of tank replicates, measurement of dehydration…etc).
Response: We submitted a supplementary method as individual file in this revision. The osmotic stress treatments are described in detail in L106-121.

Similarly, please indicate with further details how the conchocelis-dwelling shell experiment with the CA inhibitor acetazolamide was run (field vs lab? Tanks volume and replicates? How was the acetazolamide solution prepared (generally needs to be dissolved in basic solution, which could have implications for the experiment)? Was the medium refreshed during the 15 days experiment to ensure acetazolamide concentration was stable? How was the Ca concentration measured?).
Response: The inhibitory experiments are described in method section in L497-509.

Same as above for the stable isotope experiment, please also indicate the level of enrichment of Ca13CO3 used for the incubation. Did you check the bulk level of enrichment (total 13C/12C) in addition to the compounds indicated in line 227-228 (related to this, given that both oxaloacetic acid and malic acid are intermediate, they are likely to have a very rapid turn-over in 13C)
Response: The isotope labeling experiment is described in method section in Lxx. Total 13C was measured and 39% of the total carbon was labeled with ^{13}C isotopes (L275-277). TAC is one of most important pathways of carbon flux. The detection of these ^{13}C-labeled organic acids strongly support the incorporation of ^{13}C in conchocelis. We didn't detect the 13C level of other metabolites in addition to the organic acids in TCA.

Critically, statistical analyses are missing for both Figure 4C and D with Ca measurement AND 13C enrichment (not even presented with error bars). Please provide results for statistical analyses in a supplementary table.
Response: The statistical analysis of Fig. 4c and Fig. 4d were submitted as Table S6 and Table S8 respectively.

Minor comments:
Line 44: change radiolabelled as 13C is a stable isotope
Response: Changed accordingly.

The language used in the whole Ci assimilation section is vague and would need to be re-written once subcellular localization/functional information are more conclusive for the different isoforms.
Response: We rewrote this part (L206-231). The sub-cellular localization and expression patterns of CA isoforms were described more clearly.

Figure 2C, Fig3B and Fig5C : please indicate the legend/units for x-axis (% of dehydration??)

Response: We added the legends (% water loss) to these figures.

Fig5a: x-axis: be consistent with the text for gam-specific and spo-specific genes
Response: changed accordingly.

Again, thank you for the tremendous efforts that were made in helping us to improve our manuscript. If you have any additional suggestions, please do not hesitate to contact us via telephone or email.

With warmest regards,

Yunxiang Mao and Debashish Bhattacharya

REVIEWERS' COMMENTS:

Reviewer #1 (Remarks to the Author):

The authors revised to comments properly and showed the detailed method as a supplemental file. The result from synteny analysis supports the high quality of the P. yezoensis genome and suggests duplications of CA genes might be ancient. I have a few minor comments on revised parts.

The illustration of the working model is good but Fig. 4e is not referred in main text.
Add the explanation for blue squares in Fig. 5 legend.

Reviewer #2 (Remarks to the Author):

This new version addresses the main points I have raised. I appreciated this manuscript, the model presented in Fig 4e is a great help. I noticed only two minor points:
- Regarding the gene prediction process, I recommend to clarify whether the repeats were masked before, during or after the gene prediction. If it is the latter case, the consequences on gene prediction should be clarified.
- Fig 2 and 5: It would be useful to specify in the legend what "T/C" means.

Reviewer #3 (Remarks to the Author):

Your responses to my comments (as reviewer 3) are appropriate; I have no further comments.
My only further comment is that 'plasmid' should be 'plastid' on line 217.

Reviewer #4 (Remarks to the Author):

The authors have addressed most my major comments in the revised version of this manuscript, notably with significant additional work regarding functional aspects, well summarized in a conceptual diagram in Fig 3b. Although immunolocalization is an important piece of the puzzle that is still missing, I feel the revised version of the manuscript can now be recommended for publication in Nature communication.

Mathieu Pernice

Nature Communications
Jul. 02, 2020

Dear Reviewers,

Thank you for reviewing our revised MS. Here we are submitting a revised version (all edits tracked), with item-by-item responses (regular bold font) to each reviewer comment (in *Italic* text) below.

Reviewer #1 (Remarks to the Author):

The authors revised to comments properly and showed the detailed method as a supplemental file. The result from synteny analysis supports the high quality of the P. yezoensis genome and suggests duplications of CA genes might be ancient. I have a few minor comments on revised parts.
Response: Thank you.

The illustration of the working model is good but Fig. 4e is not referred in main text. Add the explanation for blue squares in Fig. 5 legend.
Response: We cited Fig.4e in L408. Blue squares in Fig.5 were explained in L911.

Reviewer #2 (Remarks to the Author):
This new version addresses the main points I have raised. I appreciated this manuscript, the model presented in Fig 4e is a great help. I noticed only two minor points:
- Regarding the gene prediction process, I recommend to clarify whether the repeats were masked before, during or after the gene prediction. If it is the latter case, the consequences on gene prediction should be clarified.
Response: Repeats were masked before gene prediction. We clarified this points in L471 in the Methods section.

- Fig 2 and 5: It would be useful to specify in the legend what "T/C" means.
Response: We changed this term into fold-change which was explained in the legend.

Reviewer #3 (Remarks to the Author):
Your responses to my comments (as reviewer 3) are appropriate; I have no further comments.
My only further comment is that 'plasmid' should be 'plastid' on line 217.
Response: Thank you. Changed accordingly.

Reviewer #4 (Remarks to the Author):

The authors have addressed most my major comments in the revised version of this manuscript, notably with significant additional work regarding functional aspects, well summarized in a conceptual diagram in Fig 3b. Although immunolocalization is an important piece of the puzzle that is still missing, I feel the revised version of the manuscript can now be recommended for publication in Nature communication.
Mathieu Pernice.
Response: Thank you. We greatly appreciate your contribution.

Again, thank you for the tremendous efforts that were made in helping us to improve our manuscript. If you have any additional suggestions, please do not hesitate to contact us via telephone or email.

With warmest regards,

Yunxiang Mao and Debashish Bhattacharya
